# Differences of the Chest Images Between Coronavirus Disease 2019 (COVID-19) Patients and Influenza Patients: A Systematic Review and Meta-analysis

**DOI:** 10.7150/ijms.98194

**Published:** 2025-01-13

**Authors:** Yingying Han, Zhijia Wang, Xingzhao Li, Zhuan Zhong

**Affiliations:** 1 Department of Neurology, China-Japan Union Hospital of Jilin University, Changchun, Jilin Province, China, 130000, ORCID: 0000-0002-3583-0448.; 2 Department of Radiology, China-Japan Union Hospital of Jilin University, Changchun, Jilin Province, China, 130000.; 3 Department of Ultrasound, China-Japan Union Hospital of Jilin University, Changchun, Jilin Province, China, 130000.; 4 Department of Orthopaedics, The Second Hospital of Jilin University, Changchun, Jilin Province, China, 130000.

**Keywords:** Computed tomography, X-ray, COVID-19, influenza.

## Abstract

**Background:** Coronavirus disease 2019 (COVID-19) and influenza are two infectious diseases that can pose a great threat to human health. We aimed to compare the differences in chest images between patients with COVID-19 and influenza to deepen the understanding of these two diseases.

**Methods:** We searched PubMed, Embase and Web of Science for articles published before December 25, 2023, and performed a meta-analysis using Stata 14.0 with a random-effects model. The study was conducted in accordance with the Preferred Reporting Items for Systematic Reviews and Meta-Analyses (PRISMA) guidelines.

**Results:** Twenty-six articles with 2,159 COVID-19 patients and 1,568 influenza patients were included in the meta-analysis. By comparing chest computed tomography (CT) and chest X-ray, we found that COVID-19 patients had more peripheral lung lesions (OR=3.66, 95% CI: 1.84-7.31). Although COVID-19 patients had more bilateral lung involvement (OR=1.74, 95% CI: 0.90-3.38) and less unilateral lung involvement (OR=0.67, 95% CI: 0.44-1.02), these two results were not statistically significant. Patients with COVID-19 showed more ground-glass opacities (OR=2.83, 95% CI: 1.85-4.32), reverse halo signs (OR=3.47, 95% CI: 2.37-5.08), interlobular septal thickening (OR=2.16, 95% CI: 1.55-3.01), vascular enlargement (OR=5.00, 95% CI: 1.80-13.85) and crazy-paving patterns (OR=2.63, 95% CI: 1.57-4.41) on chest images than patients with influenza. We also found that compared with influenza patients, pleural effusion was rare in COVID-19 patients (OR=0.15, 95% CI: 0.07-0.31).

**Conclusions:** There are some differences in the manifestations and distributions of lesions between patients with COVID-19 and influenza on chest images, which is helpful to distinguish these two infectious diseases.

## Introduction

In December 2019, a group of patients were unfortunately infected with severe acute respiratory syndrome coronavirus 2 (SARS-CoV-2), and most of them had been exposed to the Huanan Seafood Market in Wuhan, China 1. Subsequently, the coronavirus disease 2019 (COVID-19) epidemic swept the world. On January 30, 2020, the WHO listed COVID-19 as a public health emergency of international concern and then listed it as a pandemic on March 11, 2020 2. Influenza virus is an RNA virus that causes influenza in humans and animals and belongs to the orthomyxoviridae family. The family has four genera; however, only genera A and B are clinically relevant to humans 3. Prior to the current COVID-19 pandemic, there were multiple global outbreaks of acute respiratory disease caused by influenza viruses. As early as 1918, the H1N1 influenza virus rapidly erupted and spread in Europe, North America and Asia, infecting 500 million people and causing more than 50 million deaths 4. The most recent influenza pandemic was in 2009, which spread to 214 countries between March 2009 and August 2010, resulting in 18,449 laboratory-confirmed deaths worldwide 5. Although the influenza pandemic is over, H1N1 and other influenza virus strains have been with us as seasonal viruses, leading to annual seasonal influenza epidemics 6. It is also estimated that COVID-19 will not go away and will eventually show the same seasonal peak similar to influenza 7. COVID-19 and influenza have many similarities, especially in the early stage of the COVID-19 epidemic, and patients may be misdiagnosed with influenza 8.

CT and X-ray can be used to evaluate many chest disorders, including viral chest infections. Chest radiography has been shown to be helpful in determining the prognosis of patients in previous influenza outbreaks 9. In the early stage of the COVID-19 pandemic, some countries used abnormal chest images as the sole diagnostic criteria for COVID-19 before antigen or antibody tests were widely available 10-12. With the wide application of artificial intelligence with the topics of machine learning, artificial neural network and deep learning in medicine 13, X-ray images combined with artificial intelligence in the diagnosis of COVID-19 has received more attention 14. Chest CT data can also be used in modeling with other clinical data to assess unfavorable outcomes in patients with COVID-19 15. We aimed to compare the differences in chest images between patients with COVID-19 and influenza to deepen the understanding of these two diseases and provide some guidance for clinicians to make differential diagnoses.

## Materials and methods

### Eligibility criteria

Articles that met the following requirements were included in this meta-analysis: 1) the chest image characteristics, including distributions and manifestations of lesions displayed by the patients, were recorded in the sample, 2) patients were divided into an experimental group and a control group, which were COVID-19 patients and influenza patients, respectively and 3) there was no restriction on the language of the article text.

The exclusion criteria: 1) nonhuman studies, 2) case reports, 3) reviews, comments or abstracts, 4) focused on children, 5) data duplication, and 6) the sample size of the experimental group or control group was less than 10.

### Information sources

We searched PubMed, Embase and Web of Science for articles published before December 25, 2023. To collect as much data as possible, we did not restrict the language of the articles and searched for topics in both titles and abstracts.

### Search strategy

The search strategy in PubMed was as follows: (((((Flu[Title/Abstract]) OR (influenza[Title/Abstract])) OR (Influenzas[Title/Abstract])) AND ((((COVID-19[Title/Abstract]) OR (2019-nCoV[Title/Abstract])) OR (Coronavirus Disease 2019[Title/Abstract])) OR (SARS-CoV-2[Title/Abstract]))) AND (((((((Chest Images[Title/Abstract]) OR (Chest Image[Title/Abstract])) OR (CT[Title/Abstract])) OR (Computed Tomography[Title/Abstract)) OR (X-ray[Title/Abstract])) OR (Radiology[Title/Abstract])) OR (Radiological[Title/Abstract])). The search strategies used for Embase and Web of Science databases are listed in **File S1**.

### Study selection process

All the articles retrieved from the databases were imported into NoteExpress software, and duplicate articles were removed by matching titles. We then conducted a preliminary screening of articles by reading the titles or abstracts. For the articles that passed the initial screening, we conducted further screening by reading the full text and finally determined which articles could be used for this meta-analysis.

### Data selection process and items

Data extraction was performed by three authors to ensure accuracy. The first two authors screened the data independently, and disagreements were adjudicated by the third author. The items recorded were mainly distributions and manifestations of lesions displayed by the patients on chest images at the time of admission.

### Study risk of bias assessment

The Newcastle-Ottawa quality assessment scale was used to assess the quality and risk of bias of the included articles. Each article had a perfect score of nine, and a total score of seven or more meant that the article had a low risk of bias and high quality.

### Reporting bias assessment

We used funnel plots and Egger's test to evaluate reporting bias assessment, and a p value<0.05 indicated that there was no reporting bias.

### Statistical analysis

This meta-analysis was in alignment with the preferred reporting items for systematic review and meta-analysis (PRISMA) guidelines. Since only dichotomous variables were included in our results, odds ratios (ORs) were used for data analysis and evaluation, and the confidence interval (CI) was set at 95%. The I^2^ statistic was used to quantify heterogeneity, and subgroup analysis was used to explore the source of heterogeneity: I^2^≤50% indicated low heterogeneity, 50<I^2^≤75% indicated moderate heterogeneity, and I^2^>75% indicated high heterogeneity 16. The statistical software was Stata 14.0, and we used a random-effects model to estimate the effect value. A p value of z test<0.05 was considered statistically significant.

## Results

### Study selection

**Figure 1:**
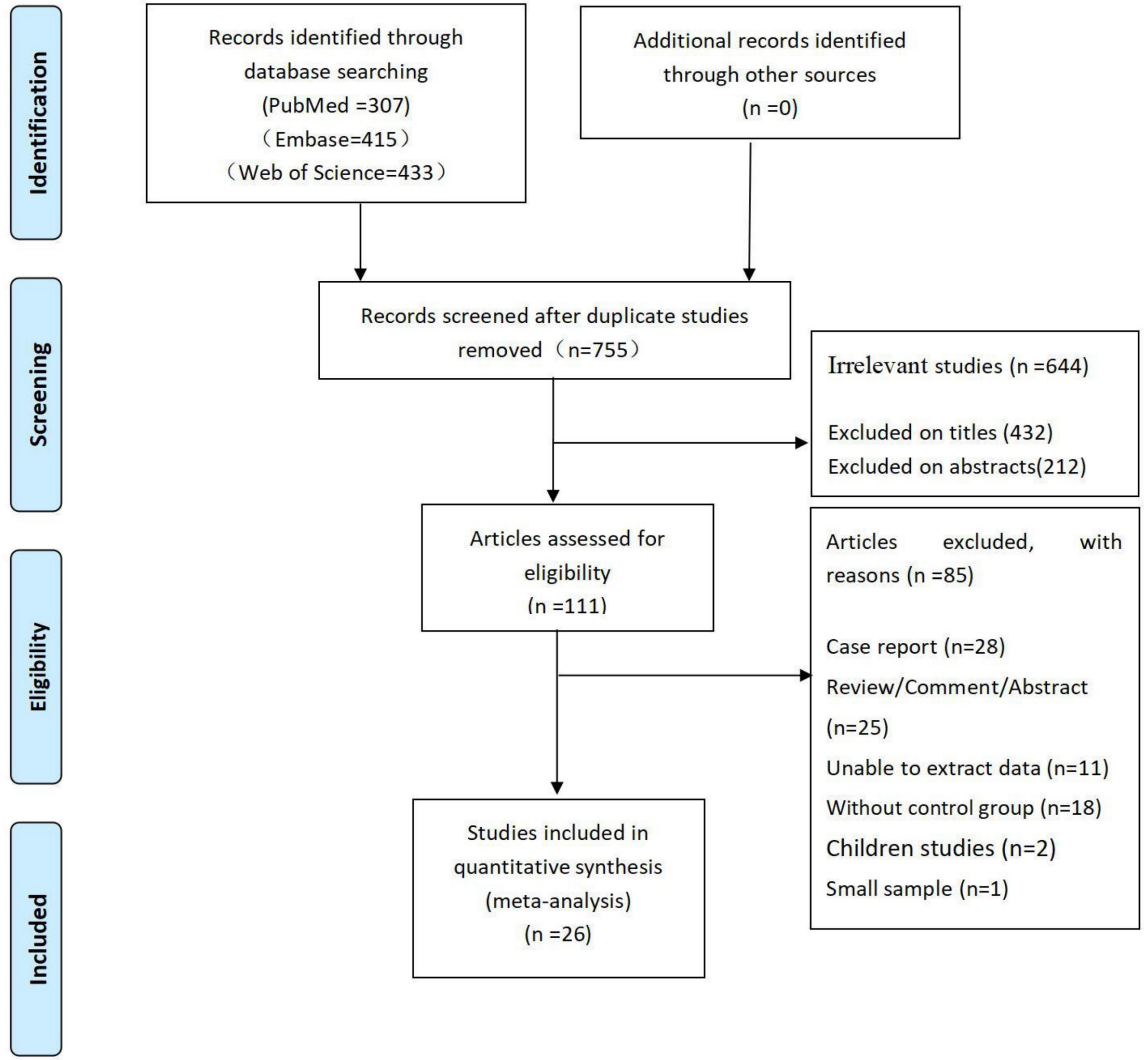
The flow diagram of the article selection process.

A total of 1,155 articles were retrieved: 307 from PubMed, 415 from Embase, and 433 from Web of Science. A total of 400 duplicate articles were removed through the duplicate identification function of NoteExpress software. Next, we removed 432 and 212 unrelated articles by reading the titles and abstracts, respectively. Among the remaining 111 articles, we further removed 85 articles by reading the full text. The detailed screening procedure is shown in **Figure 1**.

### Risk of bias in studies

The Newcastle-Ottawa quality assessment scale is listed in **Table S1**. We found that all of the studies included were of high quality and had a low risk of bias.

### Characteristics and results of individual studies

A total of 2,159 patients with COVID-19 and 1,568 patients with influenza were included in this study. The data were collected from 26 articles, covering 12 countries or regions. Except for two articles that did not describe the type of study design, the remaining articles were retrospective studies. The data on influenza patients first came from 2009, with nine articles in which patients were infected with type A and 11 articles in which patients were infected with type A/B; six articles did not describe the influenza subtype; and no articles studied patients with influenza B separately. All data on patients with COVID-19 were obtained after the COVID-19 pandemic. Most of the chest images of the patients were obtained from CT, and a few were obtained from X-ray. Most articles described the number of radiologists who reviewed the images, with a minimum of one and a maximum of six. Detailed information is provided in **Table 1**.

## Results of syntheses

### Distributions of lesions

**Figure 2:**
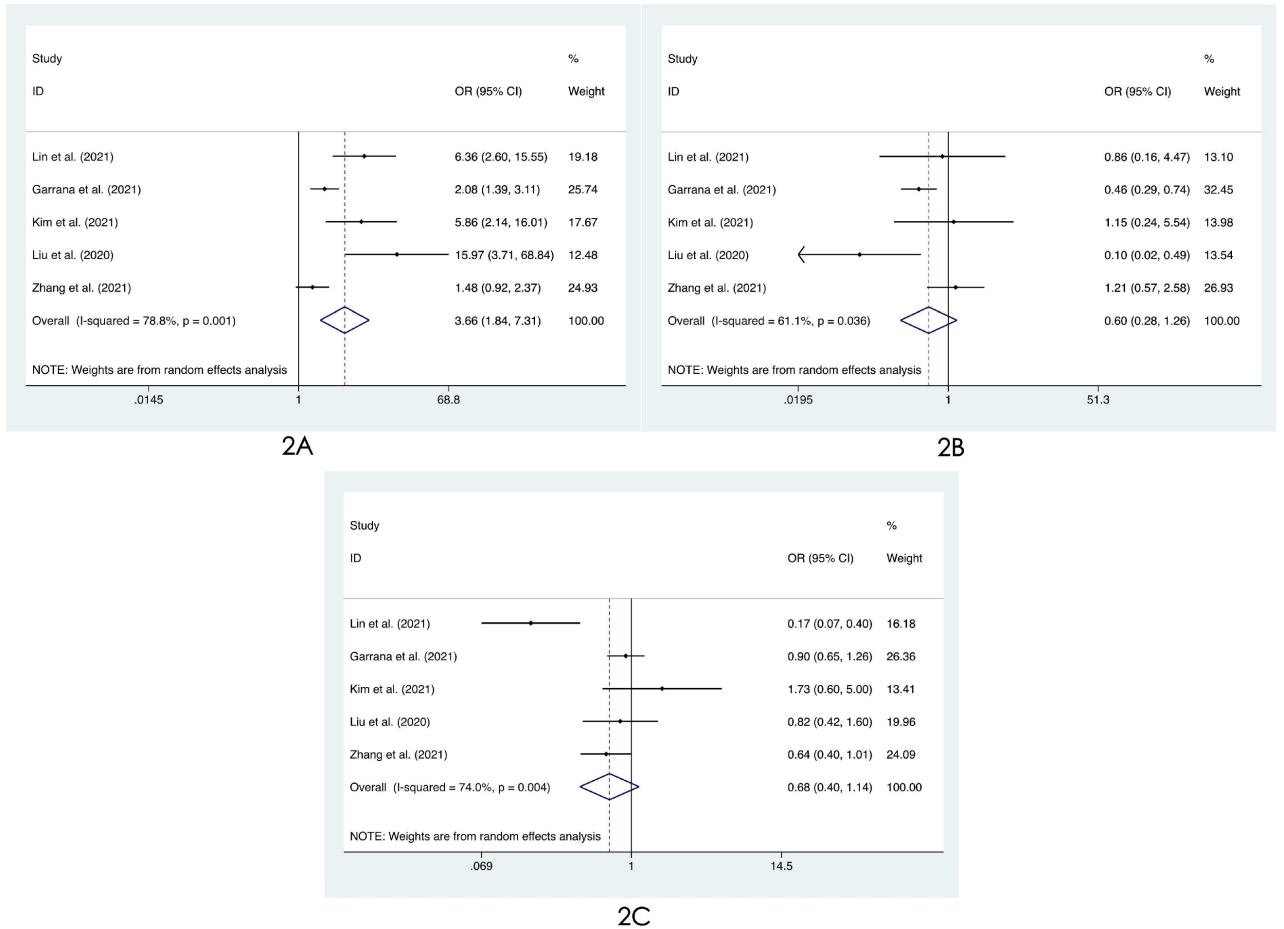
Forest plot of differences in the distribution of lung lesions on chest images between COVID-19 patients and influenza patients: **(2A)** only peripheral, **(2B)** only central, and **(2C)** both peripheral and central.

**Figure 3:**
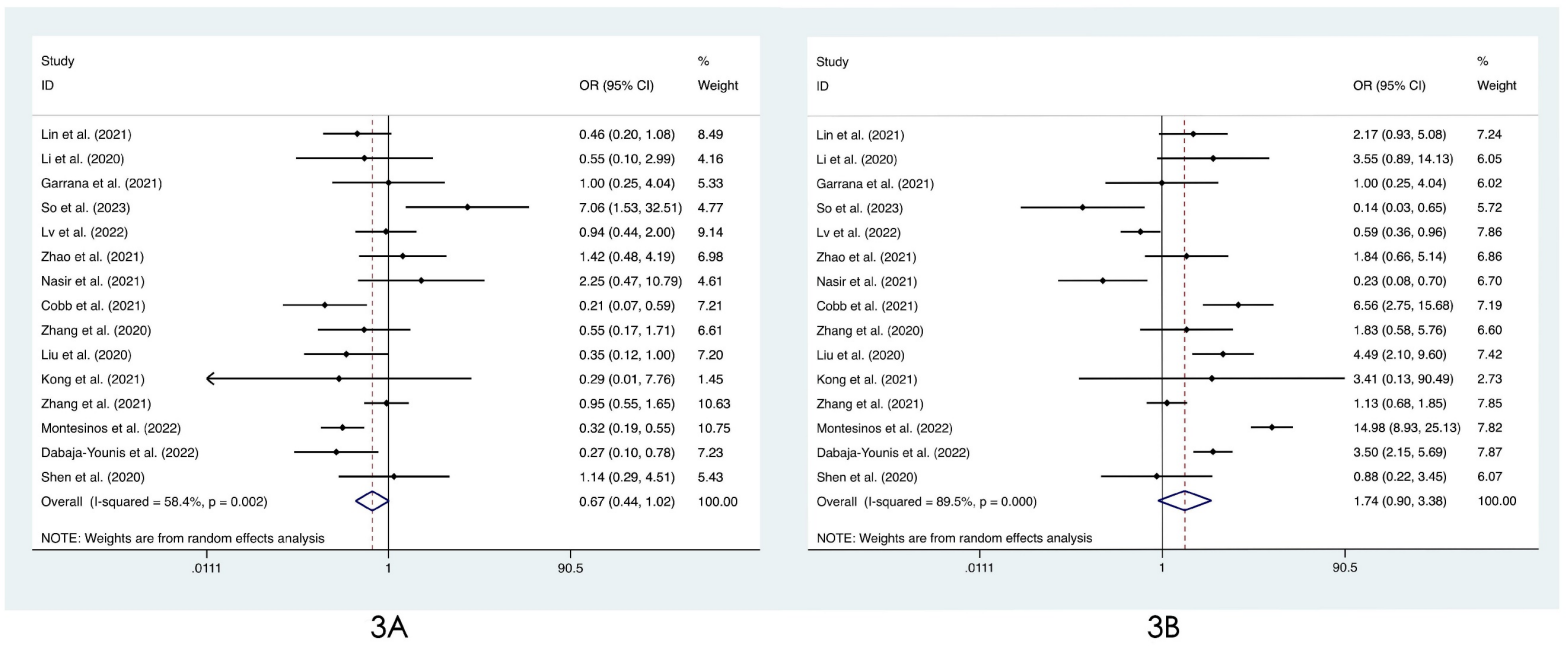
Forest plot of differences in the distribution of lung lesions on chest images between COVID-19 patients and influenza patients: **(3A)** unilateral lung and **(3B)** bilateral lung.

**Figure 4:**
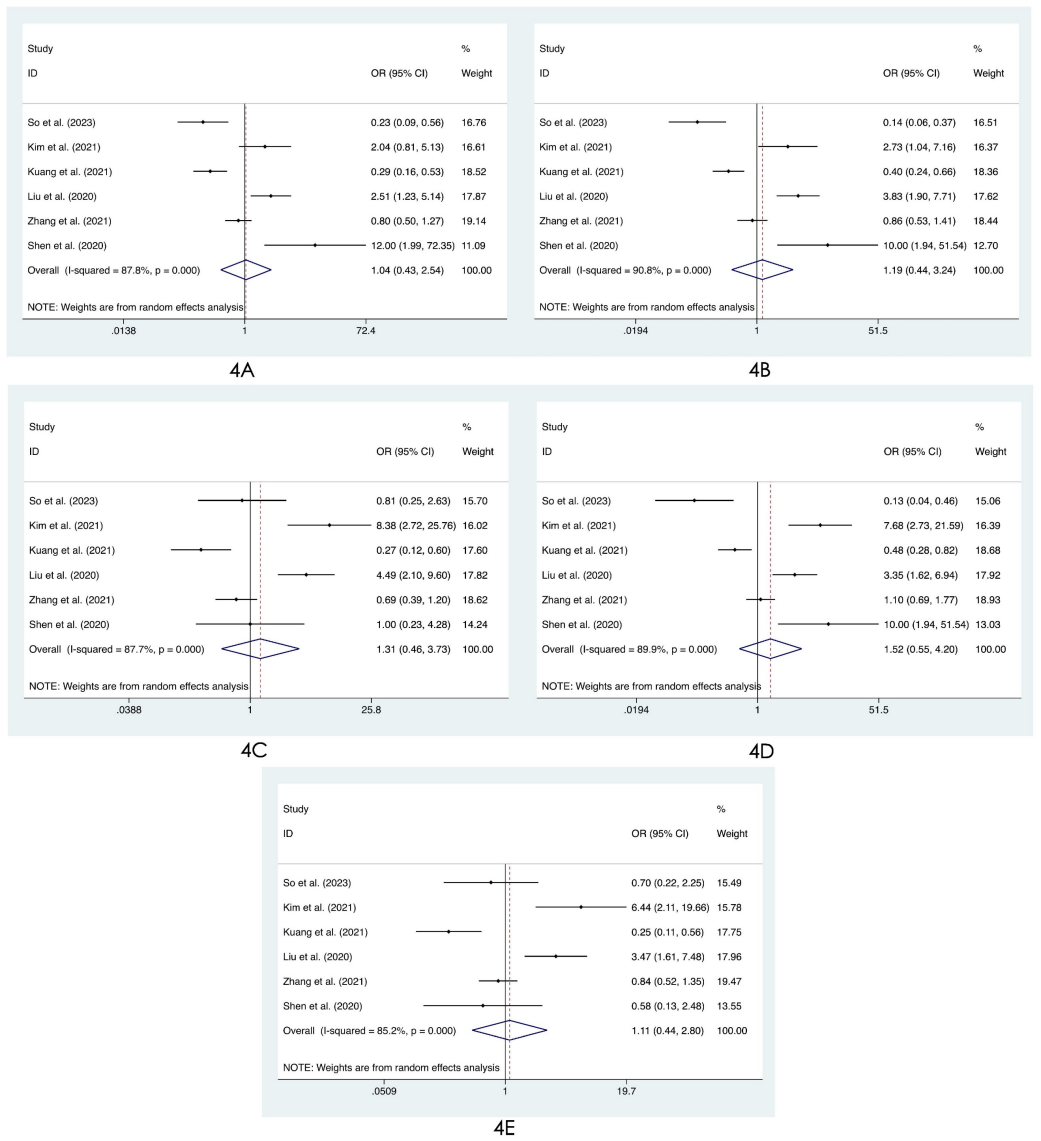
Forest plot of differences in the distribution of lung lesions on chest images between COVID-19 patients and influenza patients: **(4A)** right upper lobe, **(4B)** right middle lobe, **(4C)** right lower lobe, **(4D)** left upper lobe, and **(4E)** left lower lobe.

**Figure 5:**
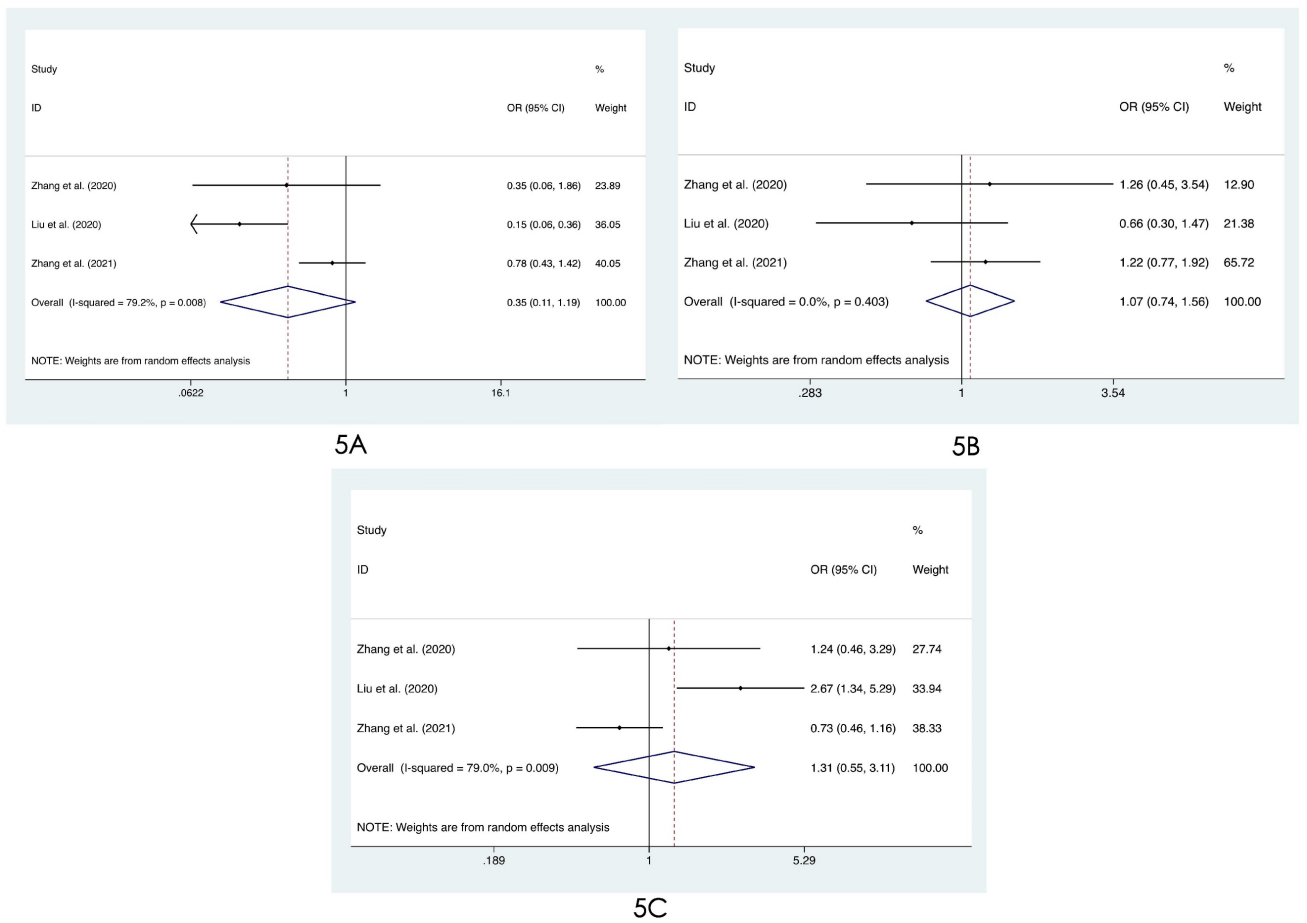
Forest plot of differences in the distribution of lung lesions on chest images between COVID-19 patients and influenza patients: **(5A)** 0-1 lobes involved, **(5B)** 2-3 lobes involved, and **(5C)** 4-5 lobes involved.

We compared the distribution of lung lesions on chest images between COVID-19 patients and influenza patients in four ways (**Tables S2-S3**). 1) We classified the location of lesions as only peripheral, only central, and both peripheral and central. We found that COVID-19 patients had more peripheral lesions (OR=3.66, 95% CI: 1.84-7.31:, I^2^=78.8%, p<0.001**, Figure 2A**), but there was no obvious difference in the distribution in the other two categories between patients with COVID-19 and influenza ((OR=0.60, 95% CI: 0.28-1.26, I^2^=61.1%, p=0.175**, Figure 2B**) and (OR=0.68, 95% CI: 0.40-1.14, I^2^=74.0%, p=0.145**, Figure 2C**)). 2) Compared with patients with influenza, COVID-19 patients had less unilateral lung involvement (OR=0.67, 95% CI: 0.44-1.02, I^2^=58.4%, p=0.059**, Figure 3A**) and more bilateral lung involvement (OR=1.74, 95% CI: 0.90-3.38:, I^2^=89.5%, p=0.100**, Figure 3B**). Although these two results were not statistically significant, both p values were near the critical value, and the results were likely to show significance when new articles were included in a future study. 3) We compared the involvement of each lobe between COVID-19 patients and influenza patients, and there were no significant differences in any of the five lobes (right upper lobe (OR=1.04, 95% CI: 0.43-2.52, I^2^=87.8%, p=0.927**, Figure 4A**), right middle lobe (OR=1.19, 95% CI: 0.44-3.24, I^2^=90.8%, p=0.729**, Figure 4B**), right lower lobe (OR=1.31, 95% CI: 0.46-3.73, I^2^=87.7%, p=0.609**, Figure 4C**), left upper lobe (OR=1.52, 95% CI: 0.55-4.20, I^2^=89.9%, p=0.417**, Figure 4D**), and left lower lobe (OR=1.11, 95% CI: 0.44-2.80, I^2^=85.2%, p=0.820**, Figure 4E**). 4) According to the number of involved lobes, we divided patients with COVID-19 and patients with influenza into three categories: 0-1 lobes involved (OR=0.35, 95% CI: 0.11-1.19, I^2^=79.2%, p=0.093**, Figure 5A**), 2-3 lobes involved (OR=1.07, 95% CI: 0.74-1.56, I^2^=0.0%, p=0.704**, Figure 5B**) and 4-5 lobes involved (OR=1.31, 95% CI: 0.55-3.11, I^2^=79.0%, p=0.541**, Figure 5C**), and no significant difference was found.

### Manifestations of lesions (Tables S4-S6, Figures S1-S18)

Patients with COVID-19 showed more ground-glass opacities (GGO) (OR=2.83, 95% CI: 1.85-4.32, I^2^=69.7%, p<0.001) and crazy-paving patterns (OR=2.63, 95% CI: 1.57-4.41, I^2^=68.5%, p<0.001) on chest images than patients with influenza. However, there was no significant difference in the proportion of consolidation between the two groups (OR=0.78, 95% CI: 0.55-1.10, I^2^=66.9%, p=0.156). We divided the patients' pulmonary nodules into two categories, namely, nodules with non-tree-in-bud (OR=0.71, 95% CI: 0.34-1.49, I^2^=76.3%, p=0.369) and nodules with tree-in-bud (OR=0.42, 95% CI: 0.13-1.37, I^2^=88.5%, p=0.152). The results showed that patients with COVID-19 and patients with influenza had approximately the same probability of having these two types of nodules. In terms of effusion, we found that pleural effusion was rare in COVID-19 patients (OR=0.15, 95% CI: 0.07-0.31, I^2^=83.4%, p<0.001), while no significant difference was shown between the two types of patients regarding pericardial effusion (OR=0.25, 95% CI: 0.57-1.26, I^2^=0.0%, p=0.164). We also found that reverse halo signs (OR=3.47, 95% CI:2.37-5.08, I^2^=0.0%, p<0.001), interlobular septal thickening (OR=2.16, 95% CI: 1.55-3.01, I^2^=0.0%, p<0.001) and vascular enlargement (OR=5.00, 95% CI:1.80-13.85, I^2^=72.7%, p<0.001) were more common on chest images of COVID-19 patients, while compared with influenza patients, there was no significant difference in the characteristics of halo signs (OR=1.14, 95% CI: 0.80-1.63, I^2^=0.0%, p=0.479), linear opacities (OR=2.08, 95% CI:0.75-5.77, I^2^=92.7%, p=0.161), cavitation (OR=0.71, 95% CI: 0.22-2.30, I^2^=48.4%, p=0.573), lymphadenopathy (OR=0.79, 95% CI: 0.55-1.14, I^2^=5.0%, p=0.211), air bronchogram (OR=1.39, 95% CI:0.85-2.24, I^2^=74.8%, p=0.186), bronchiectasis (OR=0.33, 95% CI: 0.02-5.67, I^2^=87.7%, p=0.445), bronchial wall thickening (OR=1.21, 95% CI: 0.64-2.29, I^2^=66.8%, p=0.568) and pleural thickening (OR=1.27, 95% CI: 0.54-3.00, I^2^=61.0%, p=0.588).

### Reporting biases

Funnel plots and Egger's test were used for reporting bias analysis, and most of the results were not found to have reporting bias (**Figures S19-S80**).

### Heterogeneity

In our study, a few results showed high heterogeneity, and we tried to perform a subgroup analysis by using regions or influenza subtypes as the basis for classification. However, unfortunately, we did not find the exact source of heterogeneity.

## Discussion

To the best of our knowledge, this is the first systematic review and meta-analysis to compare the differences in chest images between patients with COVID-19 and those with influenza based on case‒control studies. Pormohammad *et al.* 42 and Altmayer *et al.* 43 conducted similar studies, but their data were all from nonrandomized controlled trials, observational studies, and case series, which meant a lack of control groups. The data retrieved in both articles were up to April 2020, and related case‒control studies were lacking because the COVID-19 epidemic had just broken out at that time. Pormohammad *et al.* did not focus on chest images; they made only a simple comparison of patients' abnormal chest radiology and mainly studied the clinical characteristics and laboratory findings of the patients. Altmayer *et al.* compared adenovirus, rhinovirus, parainfluenza virus, respiratory syncytial virus and influenza virus as "other viruses" with SARS-CoV-2 and did not specifically study differences between patients with COVID-19 and those with influenza.

These two viruses primarily affect the respiratory system; SARS-CoV-2 can easily reach the periphery of the lung and, as does SARS, bind to angiotensin-converting enzyme 2 receptors in alveoli, bronchioles, and terminal bronchioles, which explains why the lesions associated with COVID-19 are mainly in the periphery of the lung. In contrast, the α2,6-linked sialic acid-bearing receptors, to which influenza viruses preferentially bind, are abundant in the human upper and lower respiratory tract, particularly in the tracheobronchial epithelium and type I alveolar cells. Thus, the pulmonary lesions of influenza are not distributed mainly in the peripheral lung but in the central or whole lung 25. GGO are hazy areas with increased lung density that do not obscure bronchial and vascular markings. The pathological features of GGO can usually be attributed to the partial displacement of air from partial filling of air spaces, thickening of interstitial tissues from fluid or cells, partial alveolar collapse, or increased capillary blood volume 44. Interlobular septal thickening is a common sign on chest CT and is visible in interstitial fluid, cellular infiltration, or fibrosis. This sign can be found in a variety of viral pneumonias 17. Our study showed that compared with influenza patients, COVID-19 patients showed more GGO and interlobular septal thickening on chest images. Therefore, it is not surprising that our other finding, that is, the proportion of crazy-paving patterns in COVID-19 patients, is higher. The crazy-paving pattern is defined as interlobular septal thickening with superimposed GGO, which is one of the worsening lesions of GGOs 22. Pleural effusion means that the pleural space is filled with fluid, which may be transudative-normal pleural fluid or exudative fluid from infection 45. Pleural effusion may indicate bacterial superinfection, which is a serious complication of COVID-19 46. Chen *et al.* noted that in COVID-19 patients, pleural effusion showed an even higher odds ratio for severe course and mortality than pulmonary consolidation (3.31 versus 2.46) 47. Our results showed that patients with influenza were more prone to pleural effusion than patients with COVID-19, which may be due to the tendency of influenza virus to affect large and small airways and lung parenchyma, leading to excessive mucus production 48. Vascular enlargement is a common imaging finding in patients with COVID-19. In the study of Ghayda *et al.*, the probability of vascular enlargement in the chest of COVID-19 patients was even higher than that of GGO (84.8% versus 60.1%) 49. The reverse halo sign is defined as a focal, rounded area of ground-glass attenuation surrounded by a more or less complete consolidation ring 50. Although the reverse halo sign is not as common as vascular enlargement in patients with COVID-19, we found that it still showed a significant difference compared with influenza patients and can be used as an imaging index to distinguish the two virus infections.

The health status after undergoing COVID-19 should also be paid enough attention. Infection with SARS-CoV-2 may have a long-term consequences on patients. Kozlik *et al.* pointed out that chronic kidney disease, diabetes mellitus, sex and vaccination could affect the quality of life after COVID-19 disease 51. The chest images of patients with COVID-19 and influenza may change with disease progression 7. Because most of the included articles were retrospective studies, we could not determine the stage of disease progression in all patients at the time of admission for imaging examination, but the patients in the experimental group and the control group were roughly at the same stage of disease at the time of screening in each included article. Therefore, the results of our study can reflect the differences in chest images of the two virus infections, which is of great significance for the diagnosis of other coronavirus diseases that may appear in the future. Accurate diagnosis of new infectious diseases during the first time can help local governments take corresponding measures to prevent the spread of the virus more quickly and control the epidemic in local areas, which can prevent the formation of a worldwide pandemic, such as COVID-19.

### Limitations

This meta-analysis has the following limitations. 1) In some of the included studies, it was not clear whether the study used blinding correctly, that is, whether the radiologist was aware of the results of the RT‒PCR test or the laboratory results, which might lead to subjective interpretation of the obtained chest image results. 2) The data came from different medical institutions, and the scanning parameters and image quality of their equipment were different, which might affect the interpretation of certain imaging details.

## Conclusions

There are some differences in the manifestations and distributions of lesions between patients with COVID-19 and influenza on chest images, which is helpful to distinguish these two infectious diseases.

## Supplementary Material

Supplementary figures and tables.

## Availability of data and materials

All data relevant to the study are included in the article/supplementary material, further inquiries can be directed to the corresponding author.

## Figures and Tables

**Table 1:** Characteristics of individual studies.

Author	Publication year	Region	Study design	Influenza subtype	Chest images	Radiologists	Study period of COVID-19	Study period of influenza
Fischer *et al.*^7^	2022	Switzerland	retrospective	NA	CT	2	2020.3-2021.3	2017-2018 2019-2020
Lin *et al.*^17^	2021	China	retrospective	A/B	CT	2	2020.1.17-2020.2.13	2018.2.20-2020.2.9
Li *et al.*^18^	2020	China	retrospective	A/B	CT	2	2020.1.19-2020.2.24	2020.1.19-2020.2.24
Garrana *et al.*^19^	2021	USA	retrospective	A/B	CT	6	2020.3.3-2020.5.1	2011.1.1-2019.12.31
So *et al.*^20^	2023	Hong Kong	retrospective	A/B	CT	2	2020.1.24-2020.4.16	2018.2.20-2020.1.30
Yang *et al.*^21^	2022	China	retrospective	NA	CT	2	2020.1.1-2020.2.15	2015.1.1-2019.9.30
Yin *et al.*^22^	2020	China	retrospective	A	CT	2	2020.2.7-2020.2.14	2018.12-2019.2
Lv *et al.*^23^	2022	China	retrospective	A	NA	NA	2020.1.17-2020.3.10	2017.11.1-2018.3.31
Kim *et al.*^24^	2021	Korea	retrospective	NA	CT	NA	2020.2.25-2020.4.1	2016.1-2020.3
Zhao *et al.*^25^	2021	China	retrospective	A/B	CT	2	2020.1.21-2020.2.9	2020.1.21-2020.2.9
Kuang *et al.*^26^	2021	China	retrospective	A	CT	2	2020.1.21-2020.2.20	2017.1.1-2020.2.29
Nasir *et al.*^27^	2021	Pakistan	retrospective	A	CT or X-ray	NA	2020	2017-2019
Cobb *et al.*^28^	2021	USA	retrospective	A/B	NA	NA	-2020.4.5	2019.1.1-2020.4.5
Zhang *et al.*^29^	2020	China	NA	A	CT	2	NA	NA
Liu *et al.*^30^	2020	China	retrospective	A/B	CT	2	2020.1-2020.2	2015.1-2020.2
Yildirim *et al.*^31^	2022	Turkey	retrospective	NA	CT	NA	2020.3.20-2020.8.1	2015.1.1-2020.2.1
Kong *et al.*^32^	2021	China	retrospective	A	CT	NA	2020.1.10-2020.3.1	2009.11.27-2009.12.312013.4.3-2013.4.30
Zhang *et al.*^33^	2021	China	NA	A/B	CT	NA	2020.1-2020.4	2018.10-2020.3
Montesinos *et al.*^34^	2022	Spain	retrospective	A/B	X-ray	NA	2020.3.1-2020.5.1	2017.1.1-2019.12.1
Faury *et al.*^35^	2021	France	retrospective	NA	CT	NA	2020.1.1-2020.3.25	2020.1.1-2020.3.25
Wang *et al.*^36^	2020	China	retrospective	A/B	CT	2	2020.1.16-2020.2.25	2019.1.1-2020.2.25
Dabaja-Younis *et al.*^37^	2022	Israel	retrospective	NA	X-ray	NA	2020.6.1-2020.8.31	2019.11.1-2020.8.31
Zarei *et al.*^36^	2021	Iran	retrospective	A	CT	3	2020.3.1-2020.7.20	2020.3.1-2020.7.20
Marcoux *et al.*^39^	2022	Belgium	retrospective	A/B	CT	NA	-2020.3.13	2015.1.1-2020.4.20
Shen *et al.*^40^	2020	China	retrospective	A	CT	1	2020.1.22-2020.2.20	2018-2019
Gu *et al.*^41^	2022	China	retrospective	A	CT	NA	2020.1-2020.3	2014-2016

COVID-19: coronavirus disease 2019, NA: not applicable.

## Abbreviations

CI: confidence interval
COVID-19: coronavirus disease 2019
CT: computed tomography
GGO: ground-glass opacities
OR: odds ratio
PRISMA: Preferred Reporting Items for Systematic Reviews and Meta-Analyses
RT‒PCR: reverse-transcriptase polymerase chain reaction
SARS-CoV-2: severe acute respiratory syndrome coronavirus 2
