# Supplementary material for: Differences of the Chest Images Between Coronavirus Disease 2019 (COVID-19) Patients and Influenza Patients: A Systematic Review and Meta-analysis
Source: Int J Med Sci. 2025 Jan 13;22(3):641–50. doi: 10.7150/ijms.98194 (PMC11783069; doi:10.7150/ijms.98194)
Supplement: Supplementary file 1 — Supplementary figures and tables. [file ijmsv22p0641s1.pdf]

Differences of the chest images between coronavirus disease 2019 (COVID-19) patients and influenza patients: A systematic review and meta-analysis

Supplementary materials

File S1: The search strategies used for Embase and Web of Science databases

Embase:

#18 #4 AND #9 AND #17

#17 #10 OR #11 OR #12 OR #13 OR #14 OR #15 OR #16

#16 'radiological':ti,ab

#15 'radiology':ti,ab

#14 'x-ray':ti,ab

#13 'computed tomography':ti,ab

#12 'ct':ti,ab

#11 'chest image':ti,ab

#10 'chest images':ti,ab

#9 #5 OR #6 OR #7 OR #8

#8 'sars-cov-2':ti,ab

#7 'coronavirus disease 2019':ti,ab

#6 '2019-ncov':ti,ab

#5 'covid-19':ti,ab

#4 #1 OR #2 OR #3

#3 'influenzas':ti,ab

#2 'influenza':ti,ab

#1 'flu':ti,ab

Web of Science:

#4 ((#1) AND #2) AND #3

#3 ((((((TS=(Chest Images)) OR TS=(Chest Image)) OR TS=(CT)) OR TS=(Computed Tomography)) OR TS=(X-ray)) OR TS=(Radiology)) OR TS=(Radiology)

#2 (((TS=(COVID-19)) OR TS=(COVID-19)) OR TS=(Coronavirus Disease 2019)) OR TS=(SARS-CoV-2)

#1 ((TS=(Flu)) OR TS=(influenza)) OR TS=(Influenzas)

Table S1: Newcastle–Ottawa quality assessment scale.

Study	Publication year	Selection	Comparability	Outcome	Quality assessment
Fischer et al.[7]	2022	4	2	3	good
Lin et al.[17]	2021	4	2	3	good
Li et al.[18]	2020	4	2	2	good
Garrana et al.[19]	2021	4	2	3	good
So et al.[20]	2023	4	2	3	good
Yang et al.[21]	2022	4	2	2	good
Yin et al.[22]	2020	4	2	3	good
Lv et al.[23]	2022	4	1	3	good
Kim et al.[24]	2021	4	2	3	good
Zhao et al.[25]	2021	4	2	3	good
Kuang et al.[26]	2021	4	2	3	good
Nasir et al.[27]	2021	4	2	2	good
Cobb et al.[28]	2021	4	1	3	good
Zhang et al.[29]	2020	4	2	3	good
Liu et al.[30]	2020	4	2	3	good
Yildirim et al.[31]	2022	4	2	3	good
Kong et al.[32]	2021	4	2	3	good
Zhang et al.[33]	2021	4	2	3	good
Montesinos et al.[34]	2022	4	2	3	good
Faury et al.[35]	2021	4	1	2	good
Wang et al.[36]	2020	4	2	3	good
Dabaja–Younis et al.[37]	2022	4	2	3	good
Zarei et al.[38]	2021	4	2	3	good
Marcoux et al.[39]	2022	4	2	3	good
Shen et al.[40]	2020	4	2	3	good
Gu et al.[41]	2022	4	2	2	good

Table S2: Distributions of lesions on chest images of COVID-19 patients and influenza patients.

Author	COVID-19						Influenza					
	Number	Peripheral	Central	Central and peripheral	Unilateral	Bilateral	Number	Peripheral	Central	Central and peripheral	Unilateral	Bilateral
Lin et al.[17]	52	35	3	14	14	38	45	11	3	31	20	25
Li et al.[18]	22	NA	NA	NA	2	19	39	NA	NA	NA	6	25
Garrana et al.[19]	300	82	31	187	4	296	300	46	60	194	4	296
So et al.[20]	66	NA	NA	NA	15	51	50	NA	NA	NA	2	48
Lv et al.[23]	222	NA	NA	NA	24	110	96	NA	NA	NA	11	60
Kim et al.[24]	31	19	3	9	NA	NA	47	10	4	9	NA	NA
Zhao et al.[25]	24	NA	NA	NA	6	18	79	NA	NA	NA	15	49
Nasir et al.[27]	115	NA	NA	NA	9	86	55	NA	NA	NA	2	51
Cobb et al.[28]	65	NA	NA	NA	5	57	73	NA	NA	NA	21	38
Zhang et al.[29]	30	NA	NA	NA	6	24	35	NA	NA	NA	11	24
Liu et al.[30]	122	50	2	60	8	104	48	2	7	26	8	27
Kong et al.[32]	16	NA	NA	NA	0	16	15	NA	NA	NA	1	14
Zhang et al.[33]	211	91	24	83	44	152	115	39	11	58	25	80
Montesinos et al.[34]	187	NA	NA	NA	23	160	187	NA	NA	NA	57	53
Dabaja–Younis et al.[37]	152	NA	NA	NA	5	104	136	NA	NA	NA	15	52
Shen et al.[40]	15	NA	NA	NA	8	7	18	NA	NA	NA	9	9

COVID-19: coronavirus disease 2019, NA: not applicable.

Table S3: Distributions of lesions on chest images of COVID-19 patients and influenza patients.

Author	COVID-19									Influenza								
	Number	Lobar involvement					Numbers of lobes affected			Number	Lobar involvement					Numbers of lobes affected		
		Right upper lobe	Right middle lobe	Right lower lobe	Left upper lobe	Left lower lobe	0–1	2–3	4–5		Right upper lobe	Right middle lobe	Right lower lobe	Left upper lobe	Left lower lobe	0–1	2–3	4–5
So et al.[20]	66	36	31	58	44	57	NA	NA	NA	50	42	43	45	47	45	NA	NA	NA
Kim et al.[24]	31	18	15	26	20	26	NA	NA	NA	47	19	12	18	9	21	NA	NA	NA
Kuang et al.[26]	405	224	185	296	235	290	NA	NA	NA	78	63	53	71	58	71	NA	NA	NA
Zhang et al.[29]	30	NA	NA	NA	NA	NA	2	11	17	35	NA	NA	NA	NA	NA	6	11	18
Liu et al.[30]	122	95	85	104	99	104	10	22	80	48	28	18	27	27	30	18	12	20
Zhang et al.[33]	211	75	140	157	80	131	33	104	74	115	47	80	93	41	76	22	51	49
Shen et al.[40]	15	9	10	10	10	9	NA	NA	NA	18	2	3	12	3	13	NA	NA	NA

COVID-19: coronavirus disease 2019, NA: not applicable.

Table S4: Manifestations of lesions on chest images of COVID-19 patients and influenza patients.

Author	COVID-19							Influenza						
	Number	Ground-glass opacities	Crazy-paving pattern	Consolidation	Nodules (non–tree-in-bud)	Nodules (tree-in-bud)	Pleural effusion	Number	Ground-glass opacities	Crazy-paving pattern	Consolidation	Nodules (non–tree-in-bud)	Nodules (tree-in-bud)	Pleural effusion
Fischer et al.[7]	52	37	21	16	2	3	NA	44	17	14	22	8	18	NA
Lin et al.[17]	52	44	7	28	NA	NA	0	45	32	6	26	NA	NA	10
Li et al.[18]	22	21	19	15	NA	NA	3	39	26	4	5	NA	NA	3
Garrana et al.[19]	300	285	NA	208	97	18	68	300	280	NA	228	121	107	145
So et al.[20]	66	56	11	53	NA	NA	2	50	37	11	43	NA	NA	25
Yang et al.[21]	73	57	38	17	NA	9	0	48	28	7	27	NA	6	10
Yin et al.[22]	30	27	18	29	NA	NA	4	30	22	9	26	NA	NA	16
Kim et al.[24]	31	31	NA	13	NA	NA	0	47	15	NA	14	NA	NA	4
Zhao et al.[25]	24	20	3	4	NA	NA	0	79	36	1	25	NA	NA	12
Kuang et al.[26]	405	256	185	50	NA	NA	7	78	41	19	27	NA	NA	46
Cobb et al.[28]	65	NA	NA	NA	NA	NA	7（*63）	73	NA	NA	NA	NA	NA	10
Liu et al.[30]	122	97	24	72	31	10	7	48	29	6	28	25	14	11
Yildirim et al.[31]	54	48	NA	2	NA	NA	NA	55	23	NA	6	NA	NA	NA
Kong et al.[32]	16	16	NA	10	NA	NA	1	15	10	NA	11	NA	NA	9
Zhang et al.[33]	211	141	47	109	34	29	33	115	82	11	63	11	6	19
Montesinos et al.[34]	187	NA	NA	NA	NA	NA	2	187	NA	NA	NA	NA	NA	12
Faury et al.[35]	34	29	NA	16	NA	NA	NA	22	13	NA	11	NA	NA	NA
Wang et al.[36]	13	12	NA	9	NA	0	0	92	82	NA	57	NA	22	5
Zarei et al.[38]	43	37	6	23	NA	NA	2	41	26	6	26	NA	NA	13
Marcoux et al.[39]	48	46	NA	28	NA	NA	NA	27	15	NA	19	NA	NA	NA
Shen et al.[40]	15	14	NA	NA	5	6	NA	18	18	NA	NA	3	4	NA
Gu et al.[41]	27	12	8	25	NA	NA	4	43	23	4	37	NA	NA	29

COVID-19: coronavirus disease 2019, NA: not applicable, *: corresponding sample.

Table S5: Manifestations of lesions on chest images of COVID-19 patients and influenza patients.

Author	COVID-19							Influenza						
	Number	Pericardial effusion	Reverse halo signs	Interlobular septal thickening	Vascular enlargement	Halo signs	Linear opacities	Number	Pericardial effusion	Reverse halo signs	Interlobular septal thickening	Vascular enlargement	Halo signs	Linear opacities
Fischer et al.[7]	52	NA	NA	34	10	7	NA	44	NA	NA	20	5	6	NA
Lin et al.[17]	52	NA	NA	14	NA	NA	NA	45	NA	NA	10	NA	NA	NA
Garrana et al.[19]	300	NA	102	NA	NA	40	NA	300	NA	37	NA	NA	35	NA
So et al.[20]	66	0	NA	NA	NA	NA	NA	50	4	NA	NA	NA	NA	NA
Yang et al.[21]	73	NA	NA	40	NA	NA	41	48	NA	NA	17	NA	NA	32
Yin et al.[22]	30	3	NA	NA	28	NA	27	30	6	NA	NA	20	NA	15
Zhao et al.[25]	24	0	NA	NA	16	2	11	79	7	NA	NA	7	3	42
Kuang et al.[26]	405	NA	65	NA	175	28	NA	78	NA	4	NA	16	6	NA
Liu et al.[30]	122	2	NA	74	NA	3	72	48	0	NA	15	NA	3	25
Zhang et al.[33]	211	8	NA	51	NA	17	128	115	5	NA	16	NA	5	59
Montesinos et al.[34]	187	NA	NA	NA	NA	NA	146	187	NA	NA	NA	NA	NA	45
Wang et al.[36]	13	NA	NA	0	NA	NA	NA	92	NA	NA	6	NA	NA	NA
Zarei et al.[38]	43	NA	2	NA	NA	NA	NA	41	NA	2	NA	NA	NA	NA

COVID-19: coronavirus disease 2019, NA: not applicable.

Table S6: Manifestations of lesions on chest images of COVID-19 patients and influenza patients.

Author	COVID-19							Influenza						
	Number	Cavitation	Lympha-denopathy	Air bronch-ogram	Bronch-iectasis	Bronchial wall thickening	Pleural thickening	Number	Cavitation	Lympha-denopathy	Air bronch-ogram	Bronch-iectasis	Bronchial wall thickening	Pleural thickening
Fischer et al.[7]	52	6	NA	33	10	14	5	44	1	NA	17	2	12	0
Lin et al.[17]	52	NA	NA	18	NA	6	NA	45	NA	NA	17	NA	7	NA
Li et al.[18]	22	NA	2	16	NA	NA	10	39	NA	5	20	NA	NA	17
Garrana et al.[19]	300	3	151	NA	NA	NA	NA	300	5	157	NA	NA	NA	NA
So et al.[20]	66	1	NA	31	2	NA	NA	50	8	NA	37	14	NA	NA
Yang et al.[21]	73	NA	0	41	NA	42	34	48	NA	4	23	NA	26	30
Yin et al.[22]	30	NA	3	15	1	3	27	30	NA	9	19	9	6	19
Kim et al.[24]	31	NA	5	NA	NA	NA	NA	47	NA	9	NA	NA	NA	NA
Zhao et al.[25]	24	0	2	15	NA	NA	2	79	1	10	20	NA	NA	15
Kuang et al.[26]	405	NA	NA	164	NA	NA	NA	78	NA	NA	35	NA	NA	NA
Liu et al.[30]	122	NA	2	22	NA	55	NA	48	NA	0	13	NA	12	NA
Zhang et al.[33]	211	6	NA	63	NA	71	NA	115	4	NA	15	NA	15	NA
Wang et al.[36]	13	NA	NA	6	NA	0	NA	92	NA	NA	25	NA	30	NA
Shen et al.[40]	15	NA	NA	7	NA	NA	7	18	NA	NA	3	NA	NA	6

COVID-19: coronavirus disease 2019, NA: not applicable.

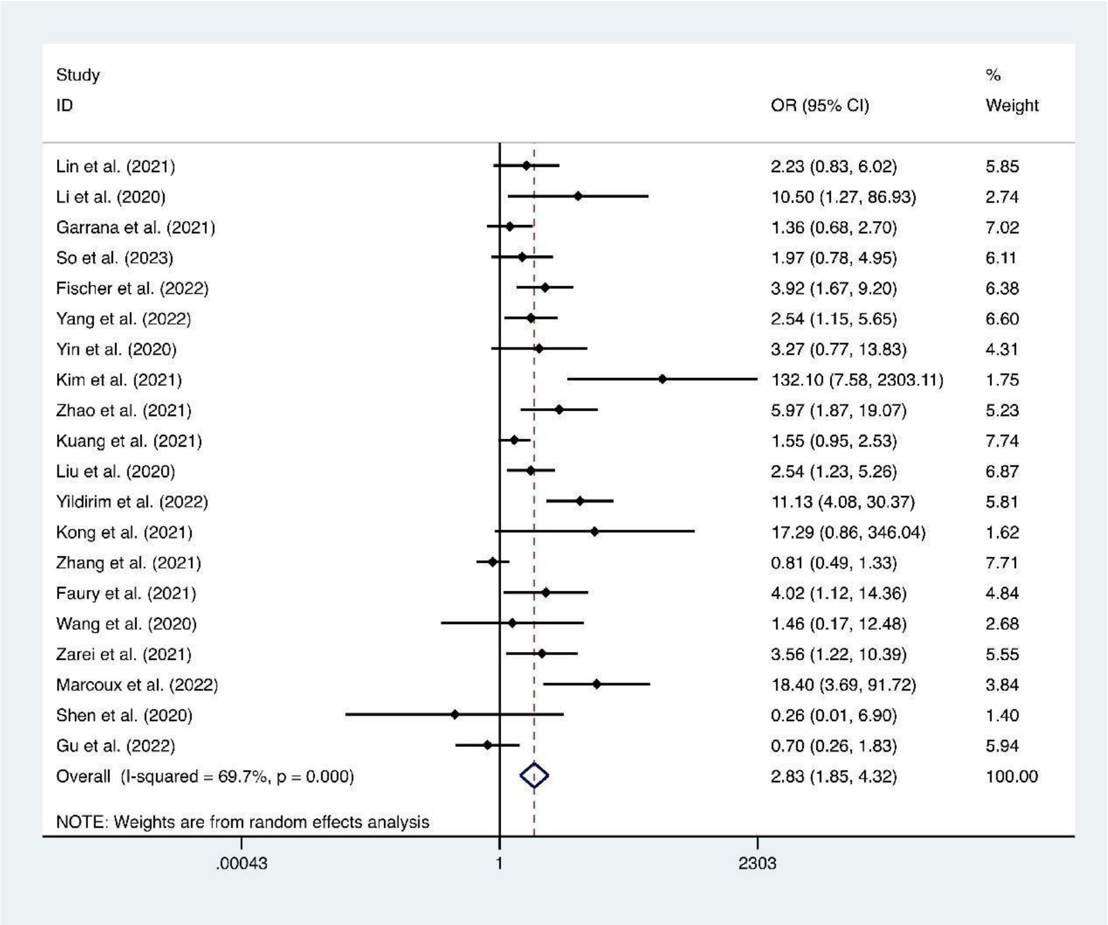

Figure S1: Forest plot of differences in the manifestation (ground-glass opacities) of lung lesions on chest images between COVID-19 patients and influenza patients

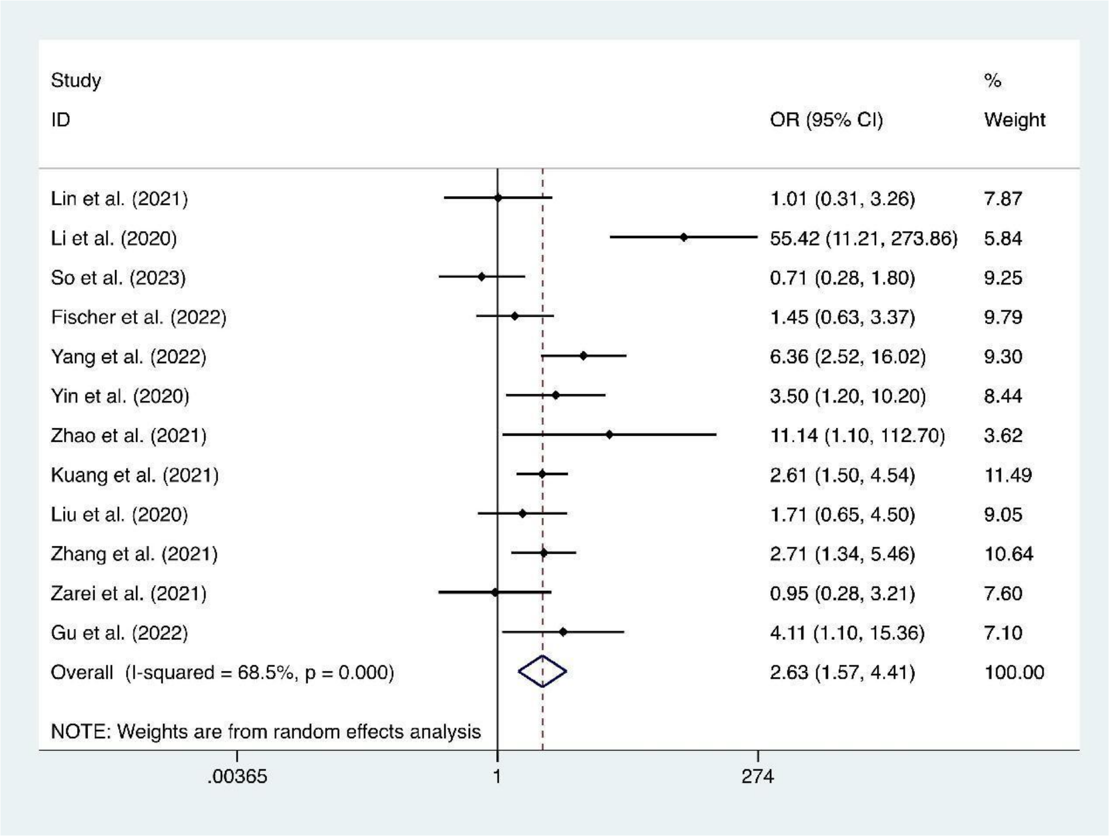

Figure S2: Forest plot of differences in the manifestation (crazy-paving patterns) of lung lesions on chest images between COVID-19 patients and influenza patients

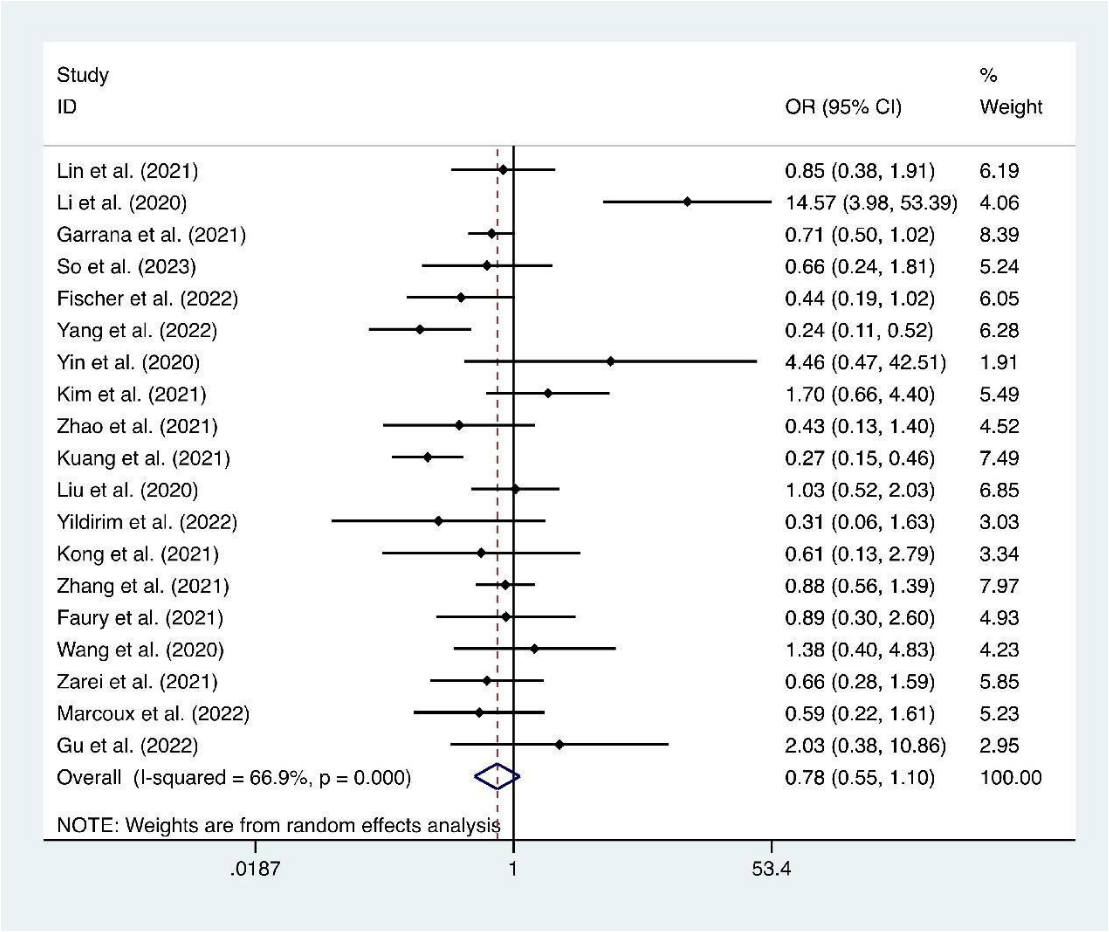

Figure S3: Forest plot of differences in the manifestation (consolidation) of lung lesions on chest images between COVID-19 patients and influenza patients

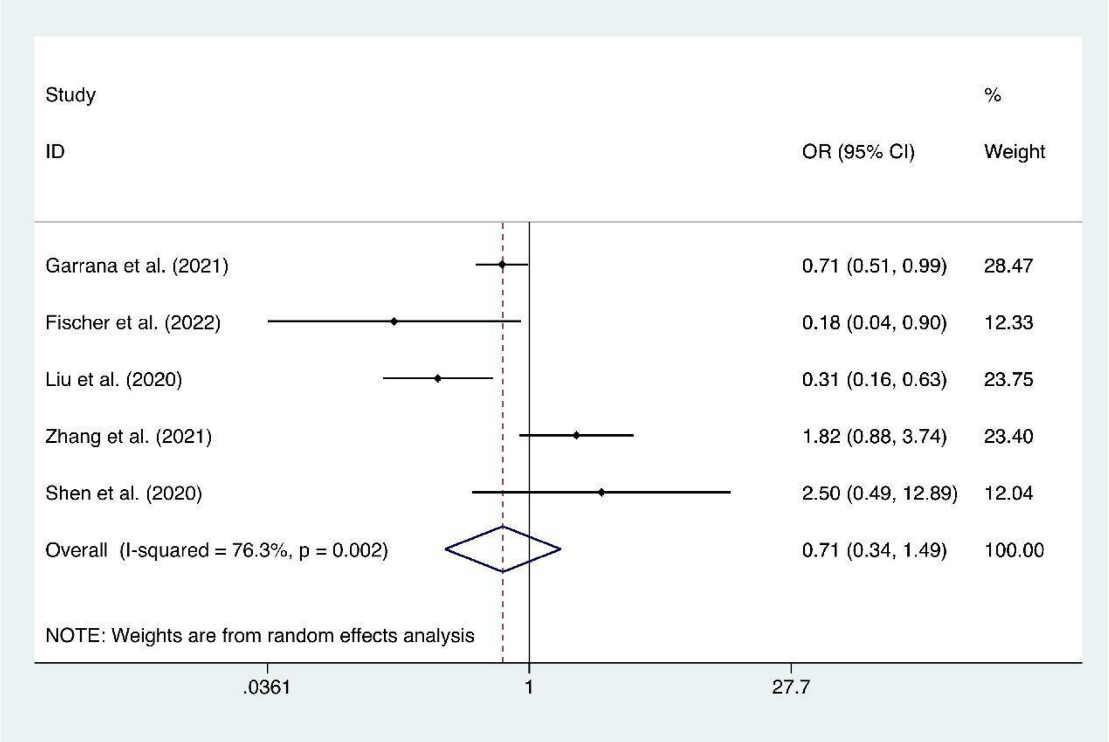

Figure S4: Forest plot of differences in the manifestation (nodules with non-tree-in-bud) of lung lesions on chest images between COVID-19 patients and influenza patients

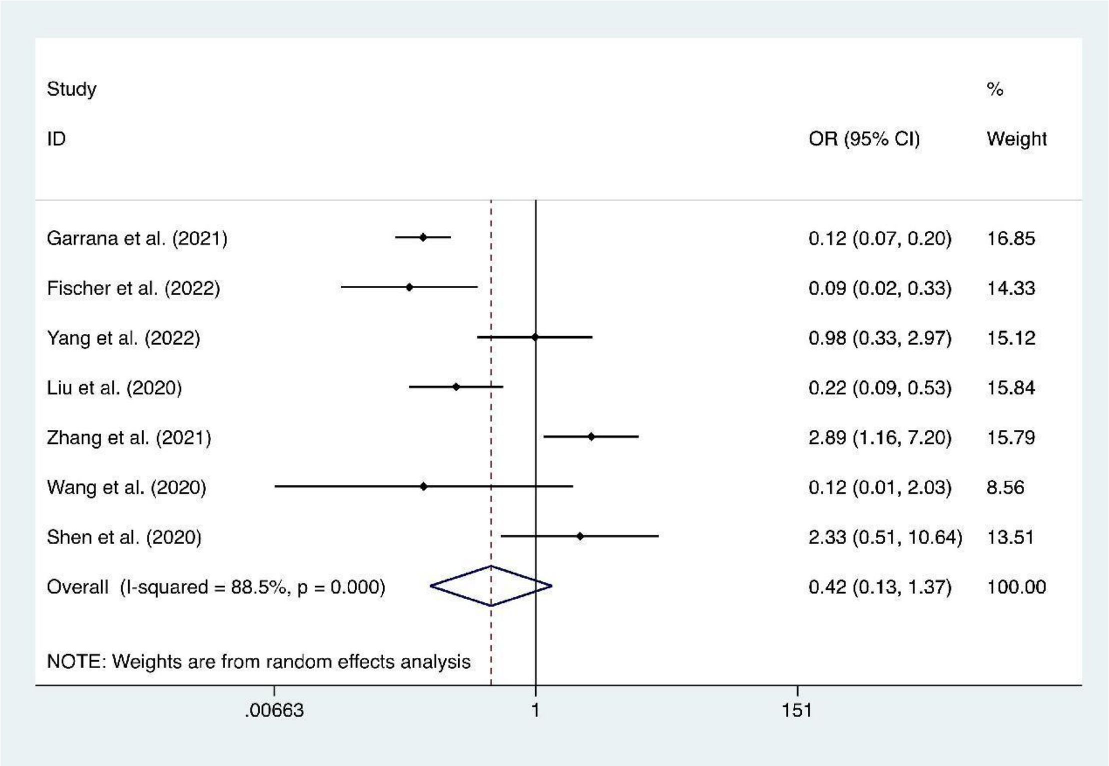

Figure S5: Forest plot of differences in the manifestation (nodules with tree-in-bud) of lung lesions on chest images between COVID-19 patients and influenza patients

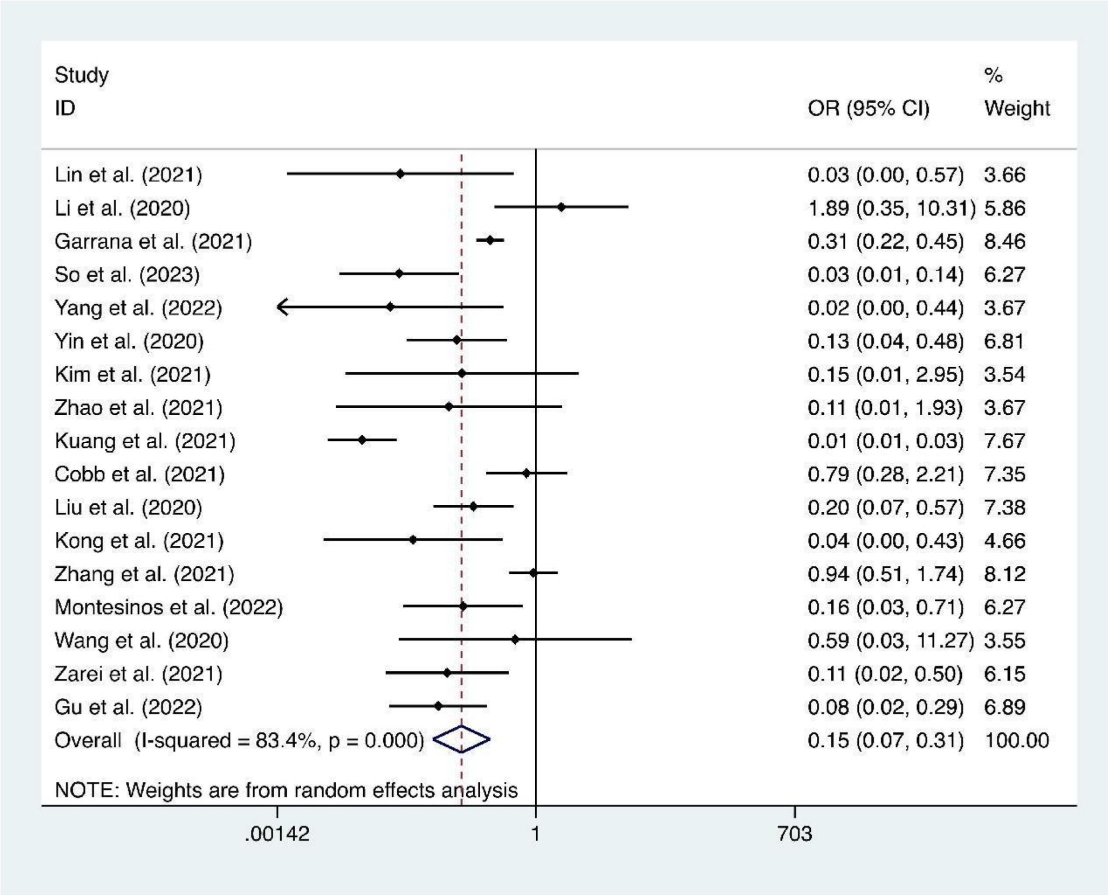

Figure S6: Forest plot of differences in the manifestation (pleural effusion) of lung lesions on chest images between COVID-19 patients and influenza patients

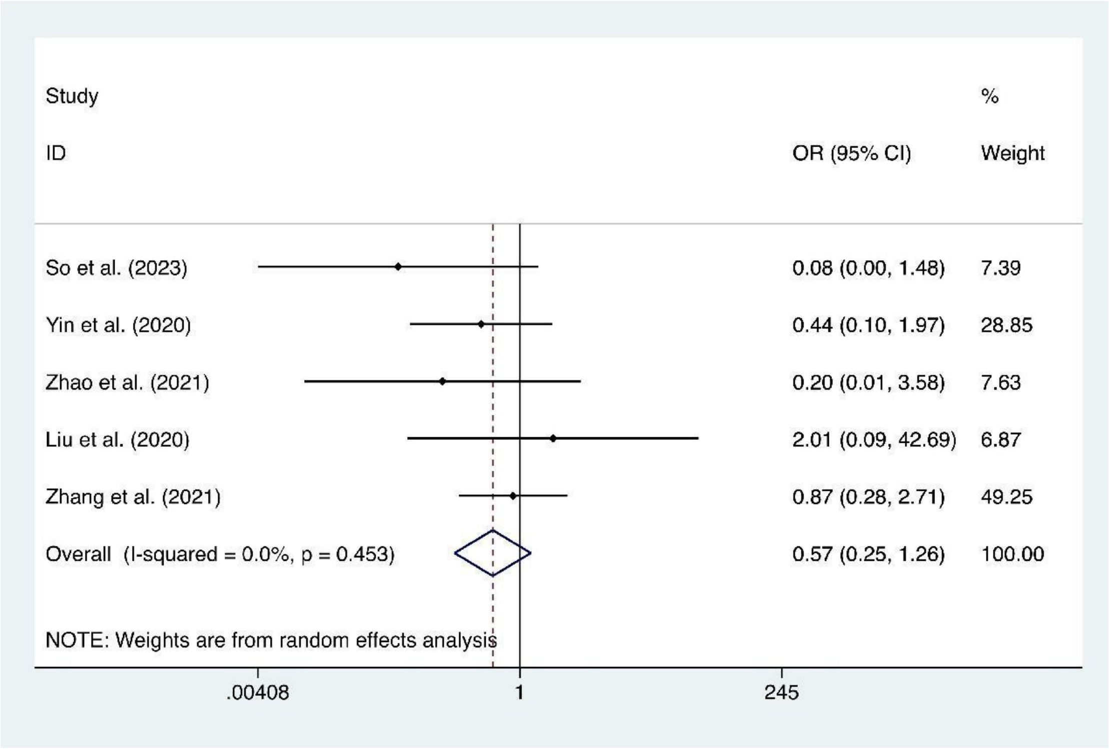

Figure S7: Forest plot of differences in the manifestation (pericardial effusion) of lung lesions on chest images between COVID-19 patients and influenza patients

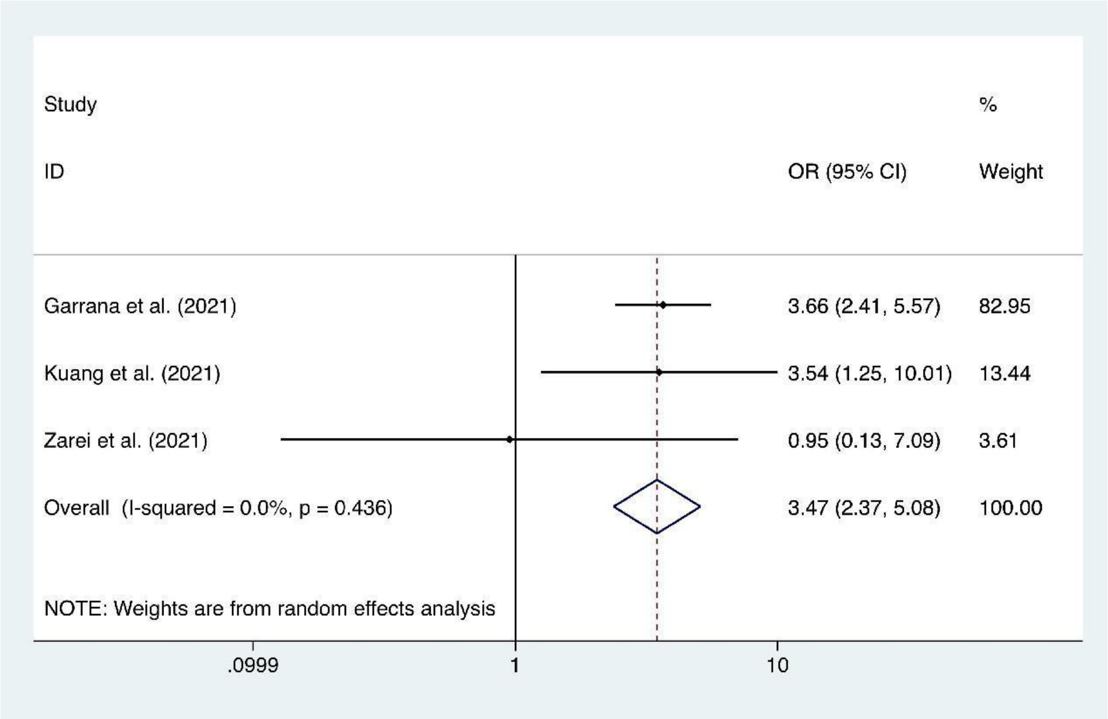

Figure S8: Forest plot of differences in the manifestation (reverse halo signs) of lung lesions on chest images between COVID-19 patients and influenza patients

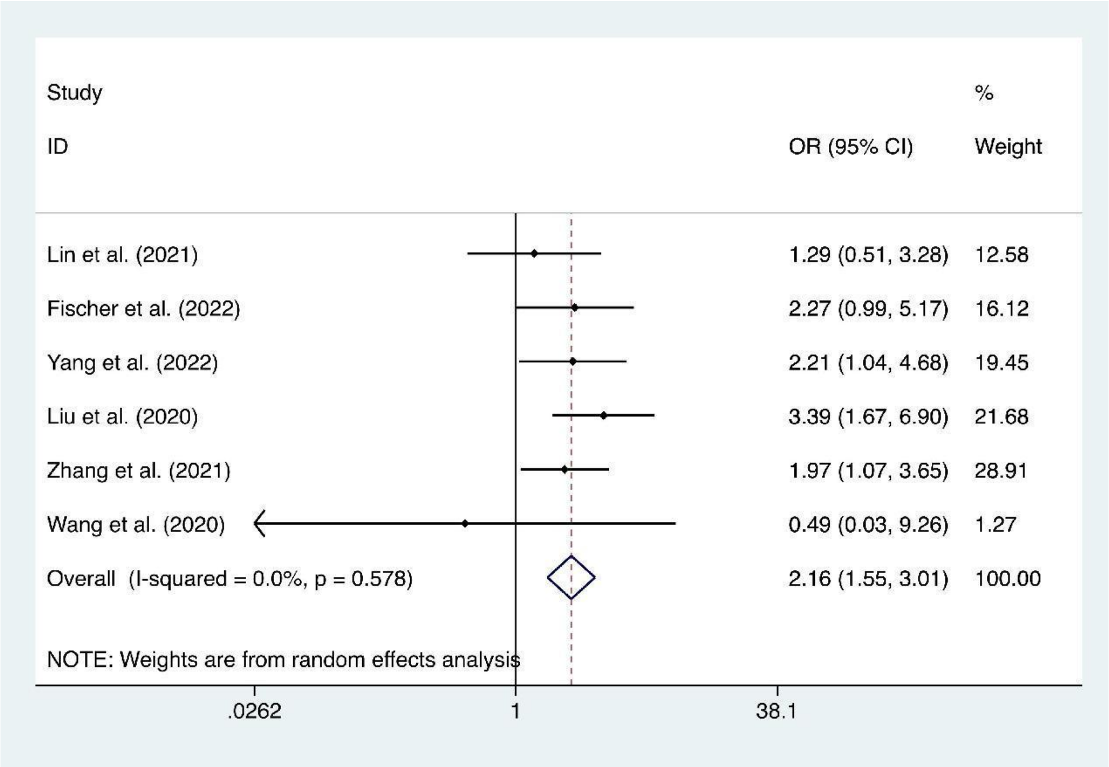

Figure S9: Forest plot of differences in the manifestation (interlobular septal thickening) of lung lesions on chest images between COVID-19 patients and influenza patients

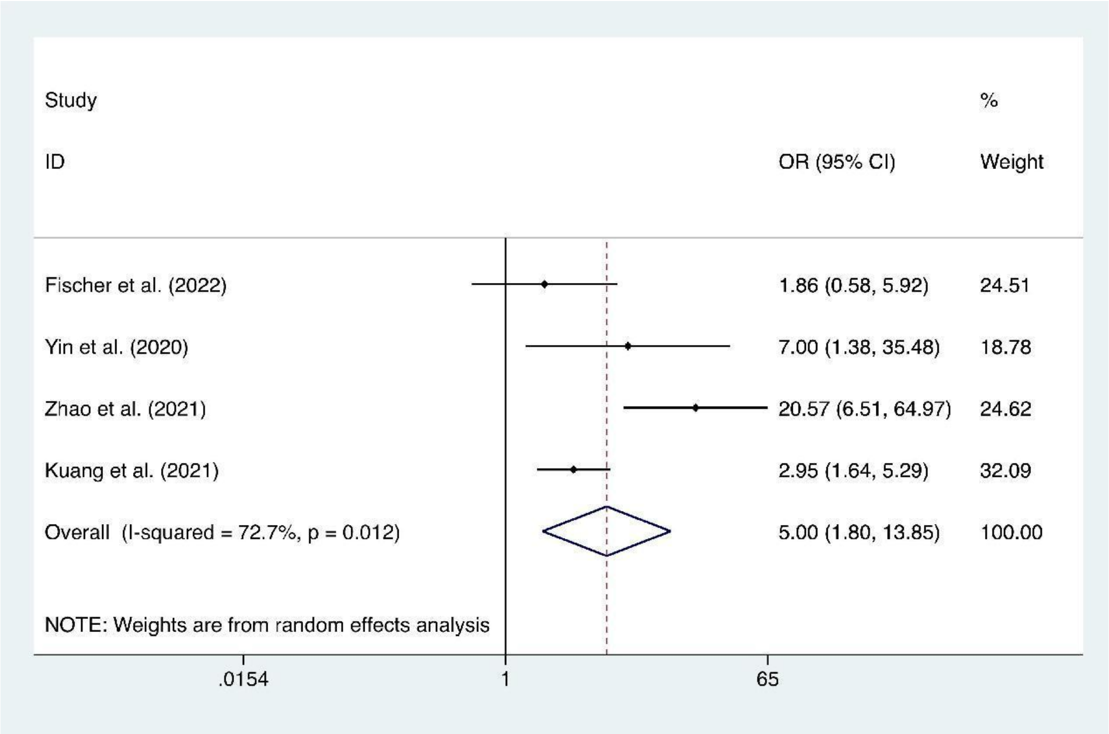

Figure S10: Forest plot of differences in the manifestation (vascular enlargement) of lung lesions on chest images between COVID-19 patients and influenza patients

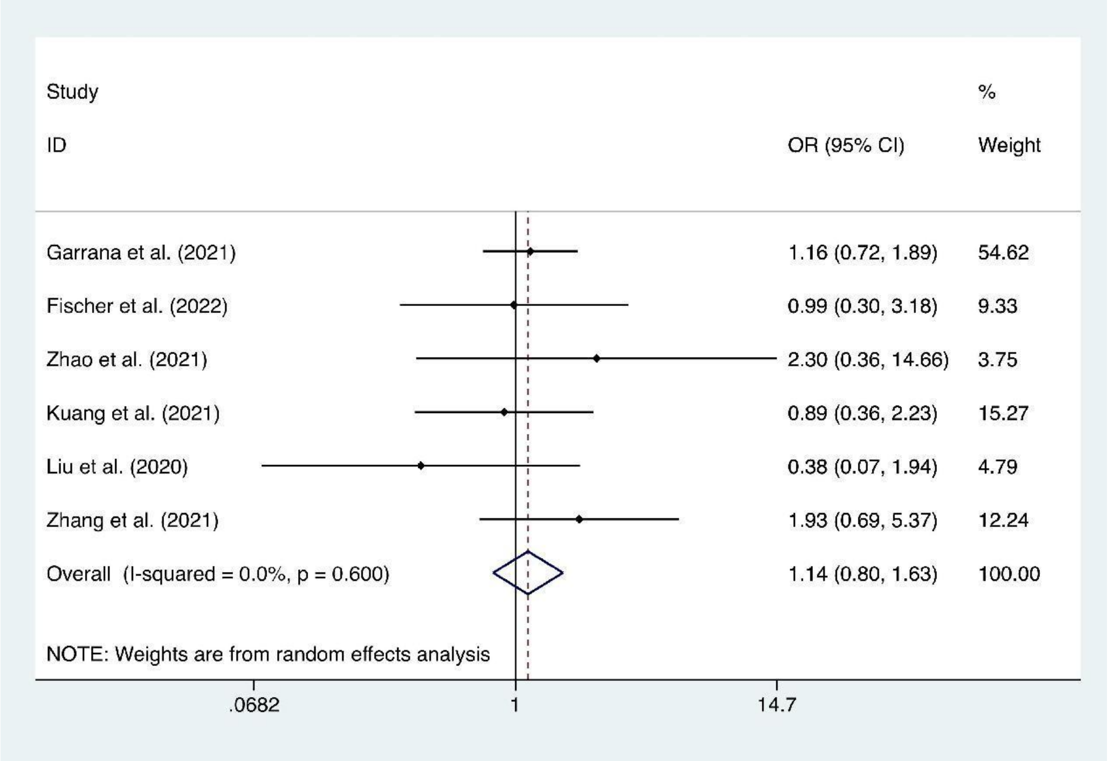

Figure S11: Forest plot of differences in the manifestation (halo signs) of lung lesions on chest images between COVID-19 patients and influenza patients

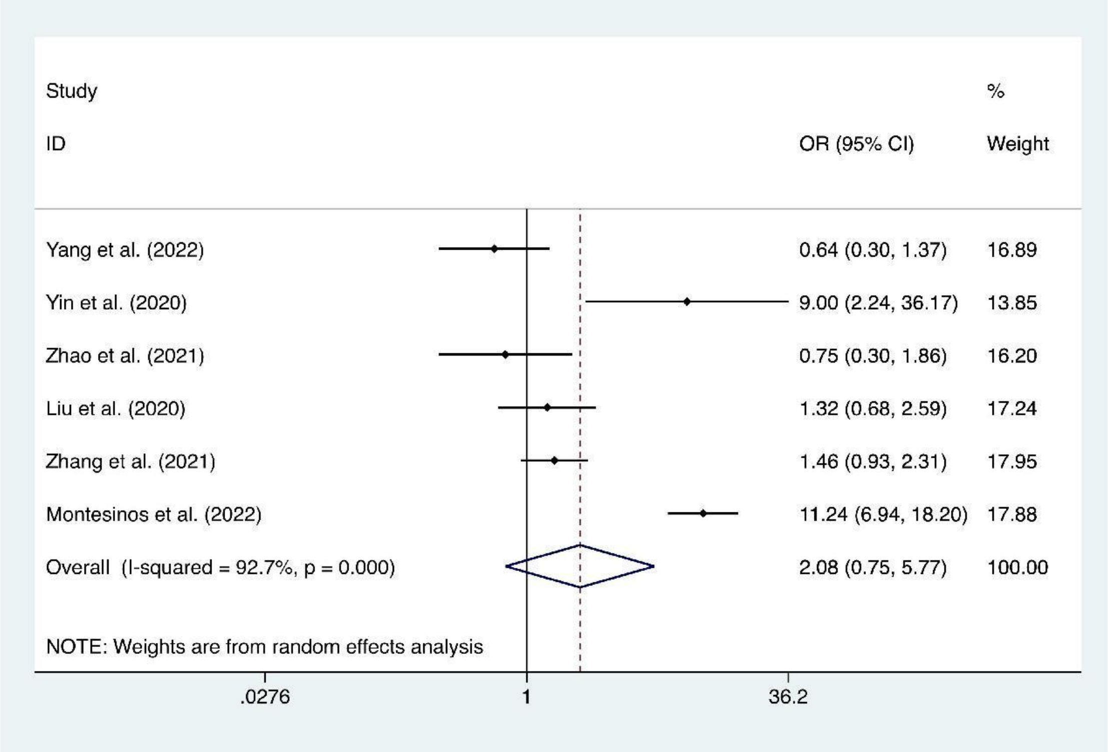

Figure S12: Forest plot of differences in the manifestation (linear opacities) of lung lesions on chest images between COVID-19 patients and influenza patients

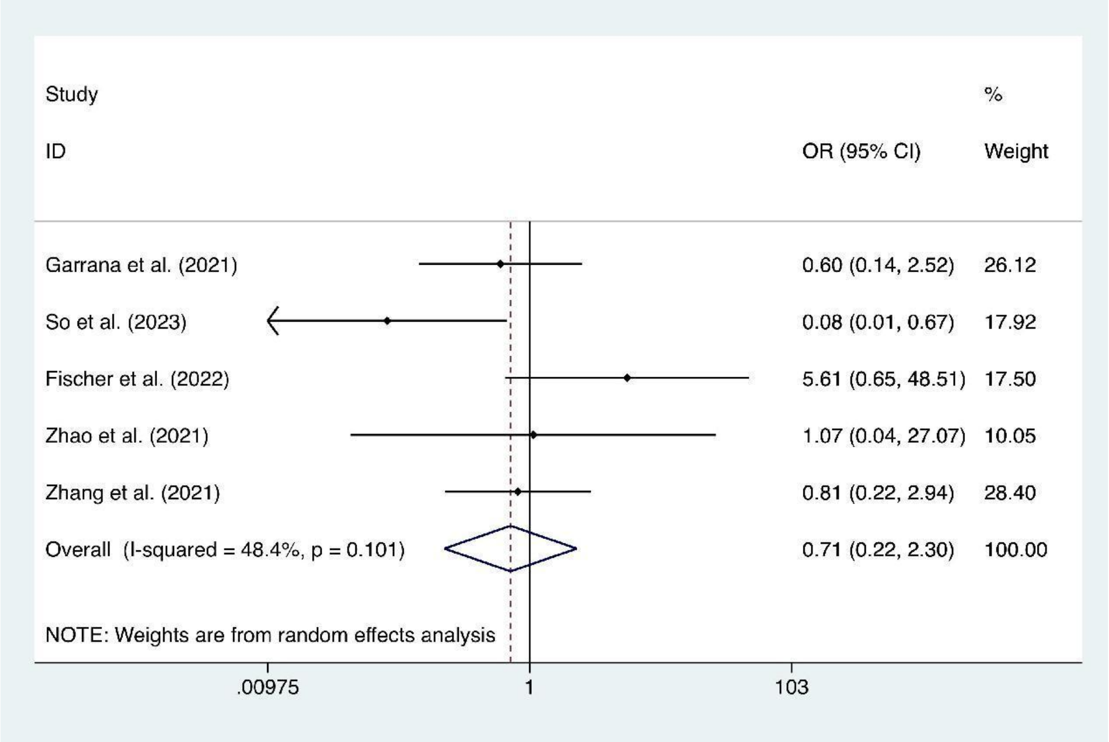

Figure S13: Forest plot of differences in the manifestation (cavitation) of lung lesions on chest images between COVID-19 patients and influenza patients

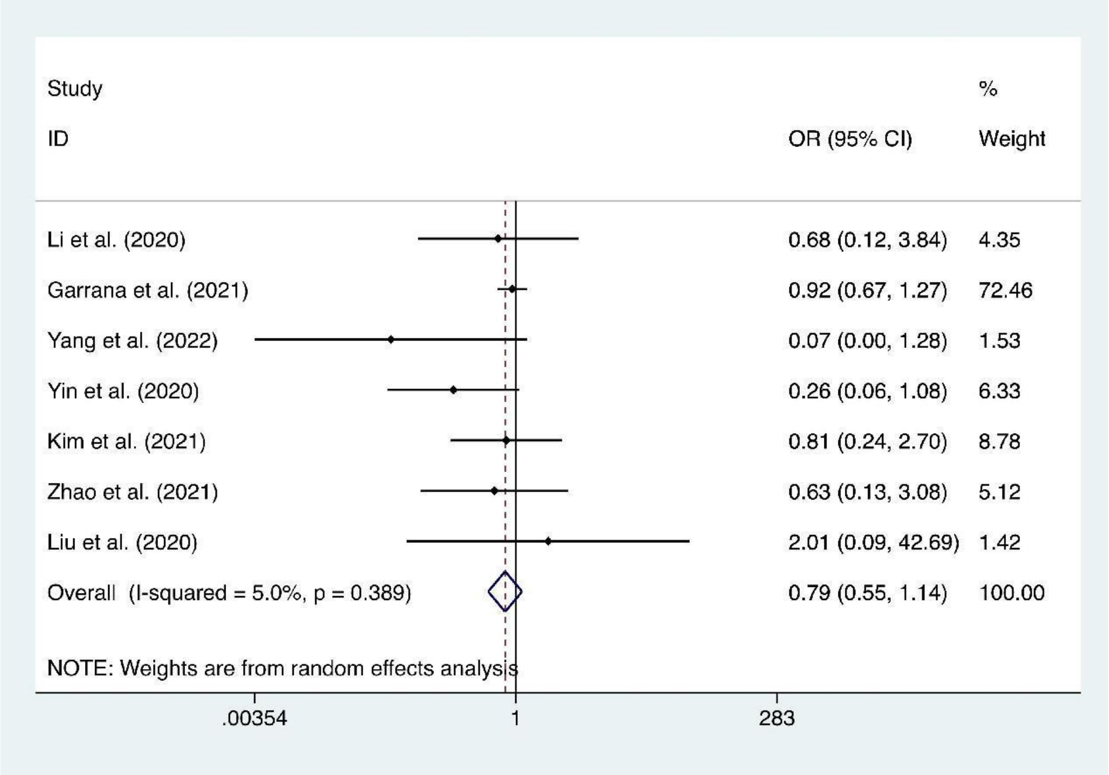

Figure S14: Forest plot of differences in the manifestation (lymphadenopathy) of lung lesions on chest images between COVID-19 patients and influenza patients

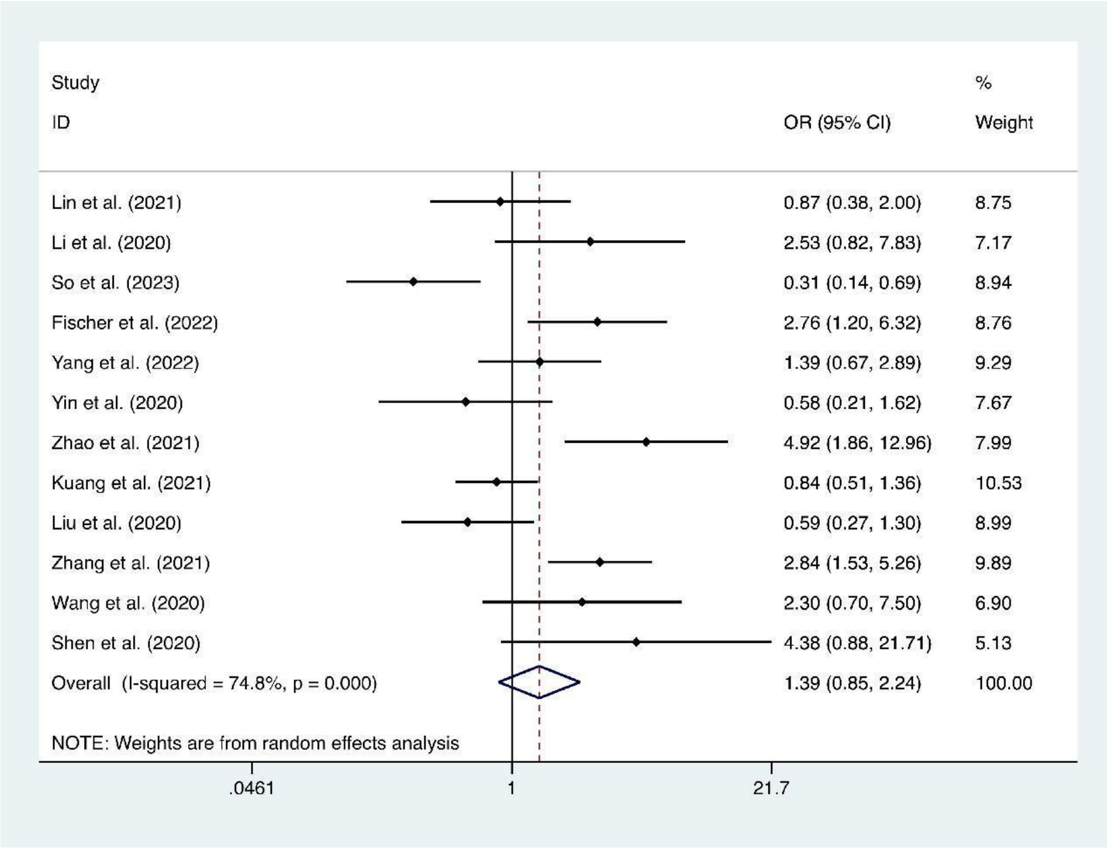

Figure S15: Forest plot of differences in the manifestation (air bronchogram) of lung lesions on chest images between COVID-19 patients and influenza patients

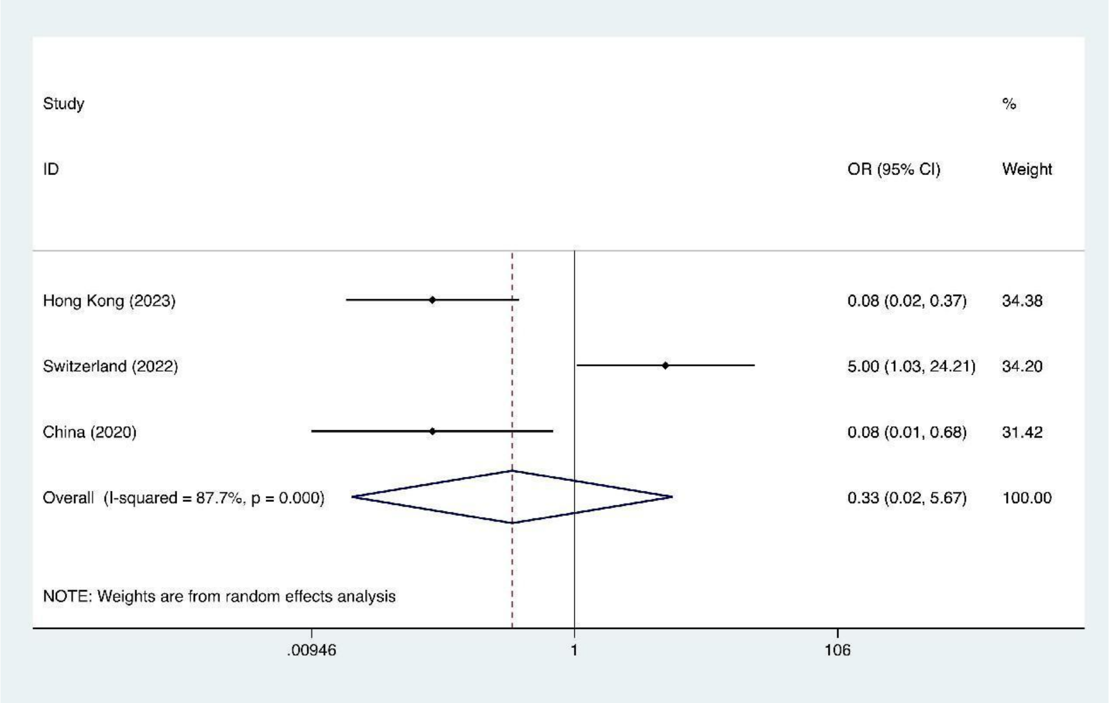

Figure S16: Forest plot of differences in the manifestation (bronchiectasis) of lung lesions on chest images between COVID-19 patients and influenza patients

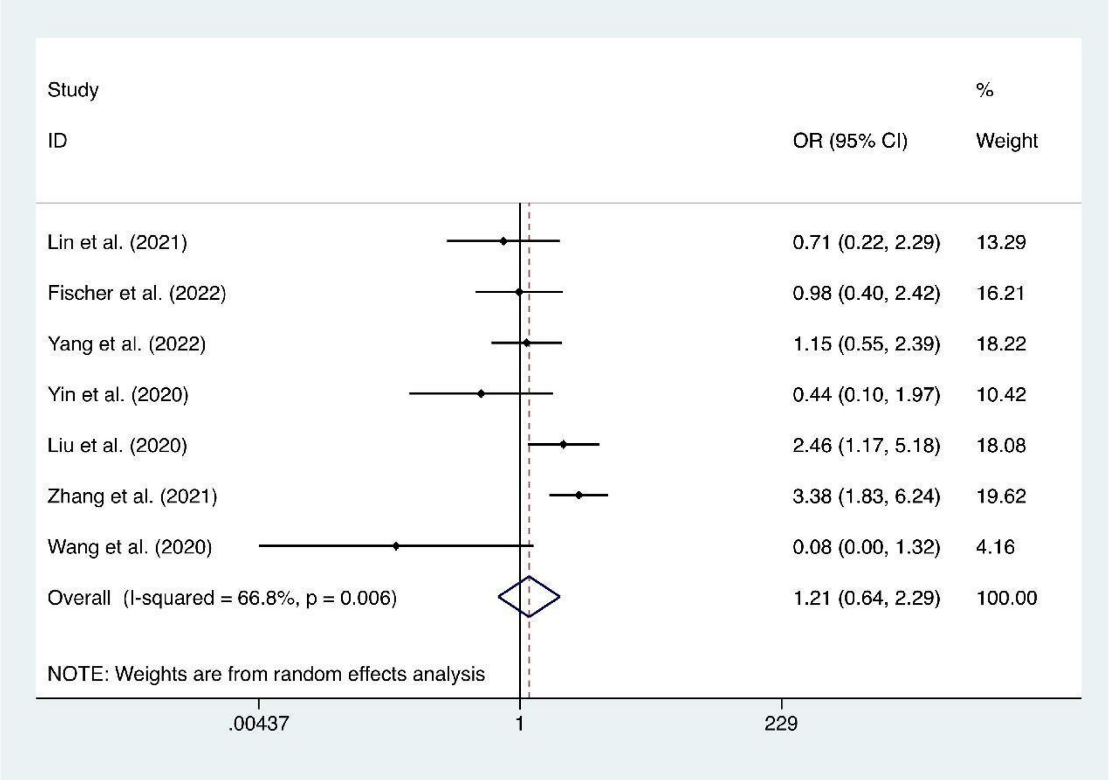

Figure S17: Forest plot of differences in the manifestation (bronchial wall thickening) of lung lesions on chest images between COVID-19 patients and influenza patients

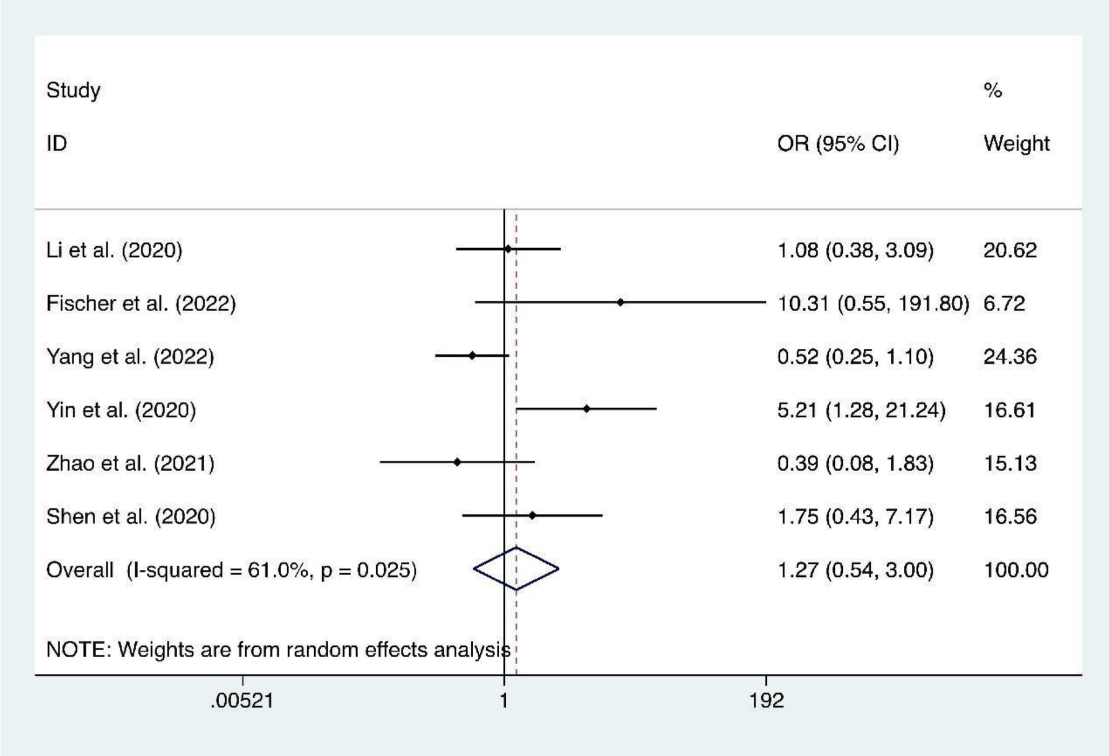

Figure S18: Forest plot of differences in the manifestation (pleural thickening) of lung lesions on chest images between COVID-19 patients and influenza patients

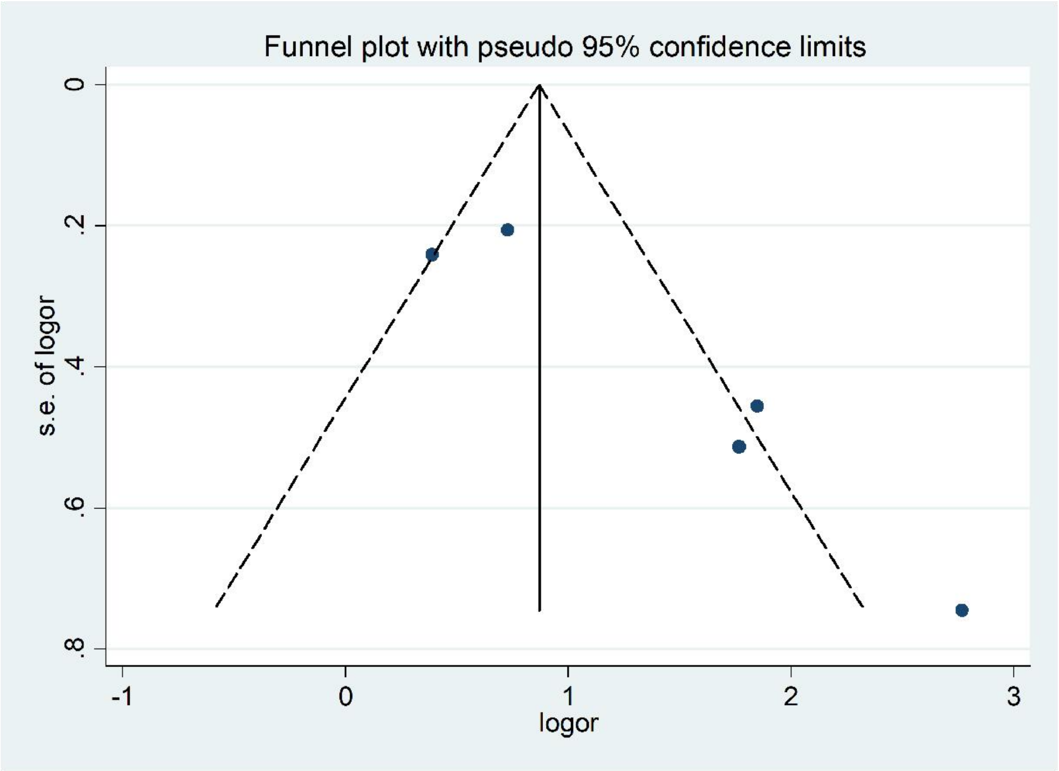

Figure S19: Funnel plot of differences in the distribution (only peripheral) of lung lesions on chest images between COVID-19 patients and influenza patients

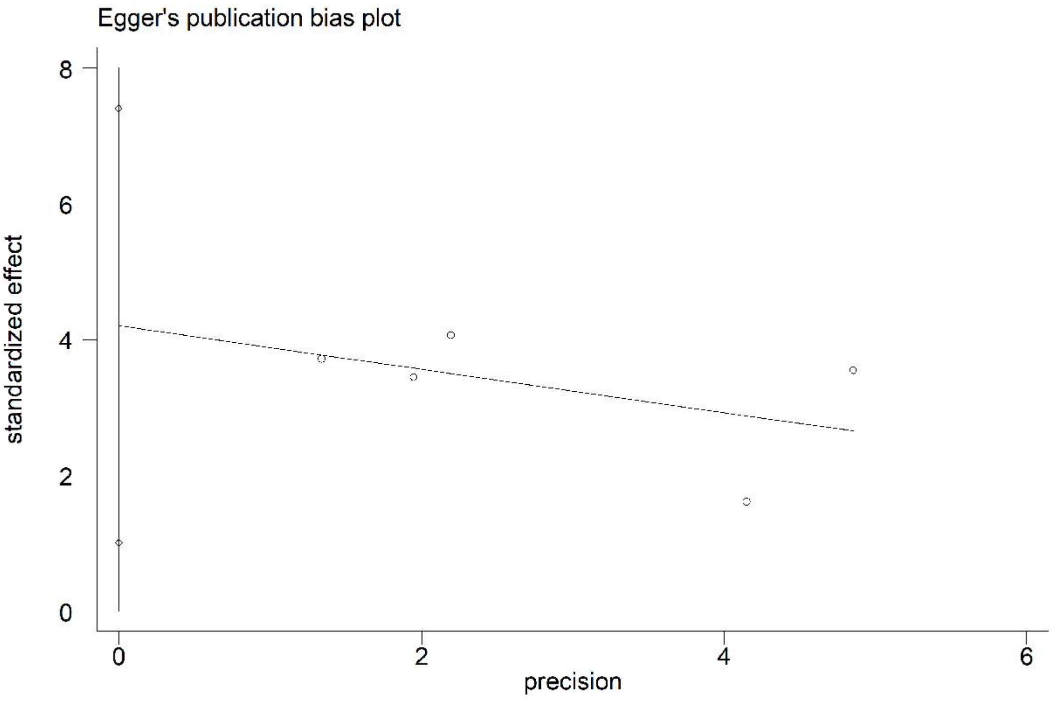

Figure S20: Egger’s publication bias plot of differences in the distribution (only peripheral) of lung lesions on chest images between COVID-19 patients and influenza patients (p=0.025)

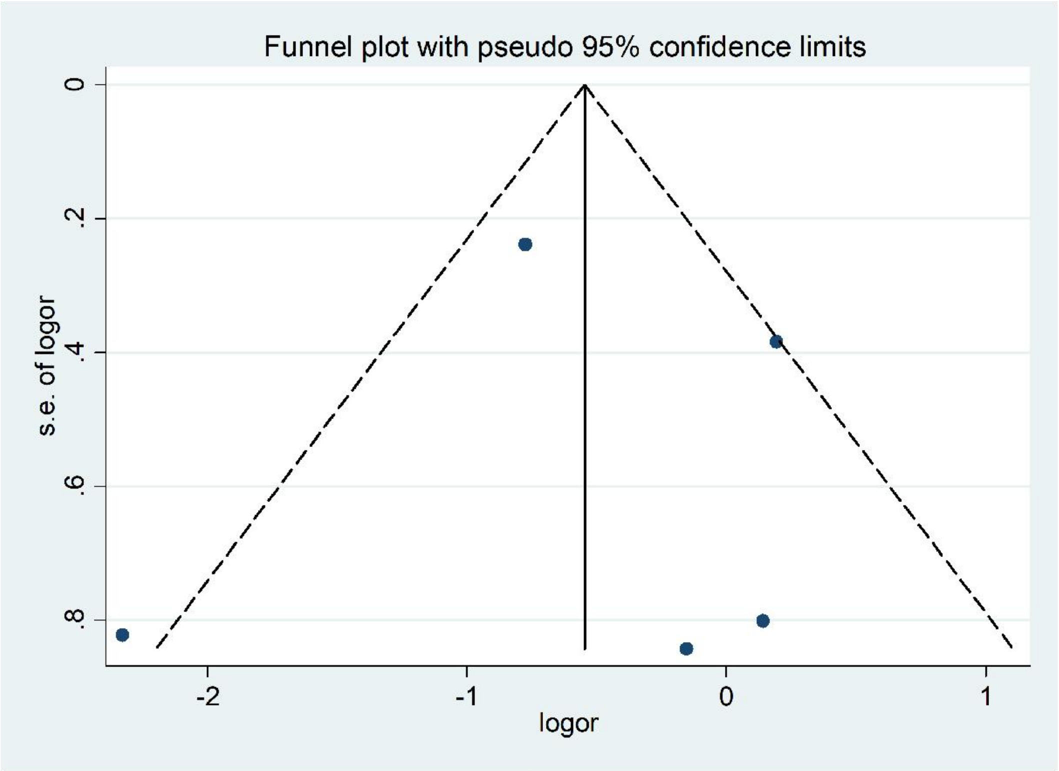

Figure S21: Funnel plot of differences in the distribution (only central) of lung lesions on chest images between COVID-19 patients and influenza patients

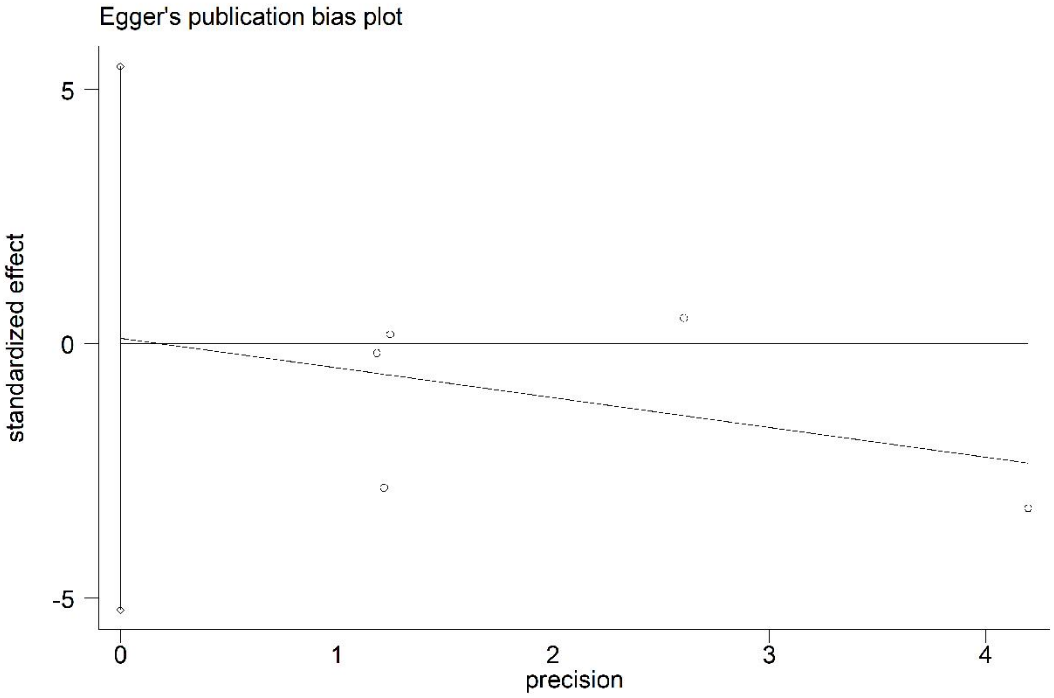

Figure S22: Egger' s publication bias plot of differences in the distribution (only central) of lung lesions on chest images between COVID-19 patients and influenza patients (p=0.953)

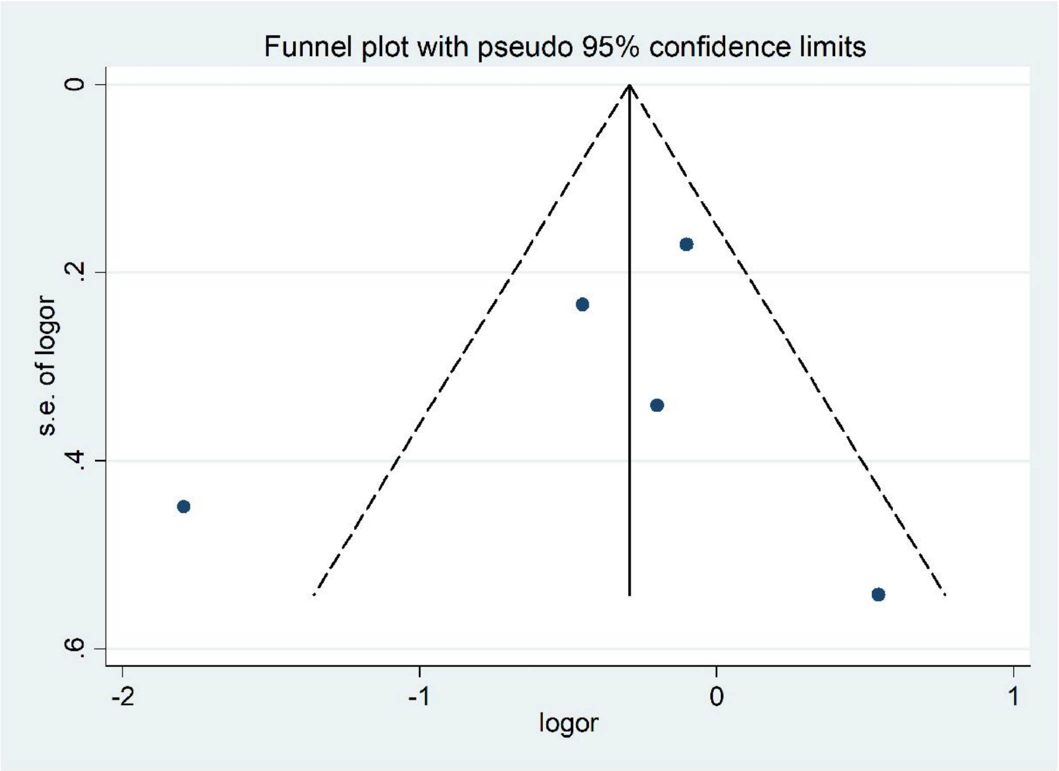

Figure S23: Funnel plot of differences in the distribution (both peripheral and central) of lung lesions on chest images between COVID-19 patients and influenza patients

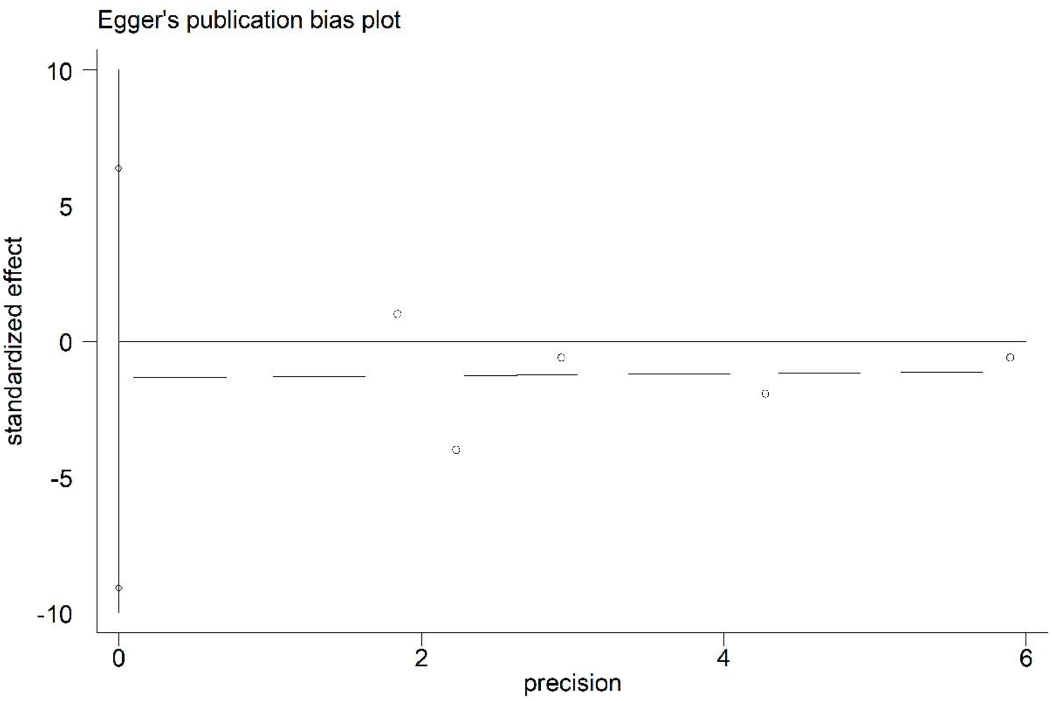

Figure S24: Egger's publication bias plot of differences in the distribution (both peripheral and central) of lung lesions on chest images between COVID-19 patients and influenza patients (p=0.617)

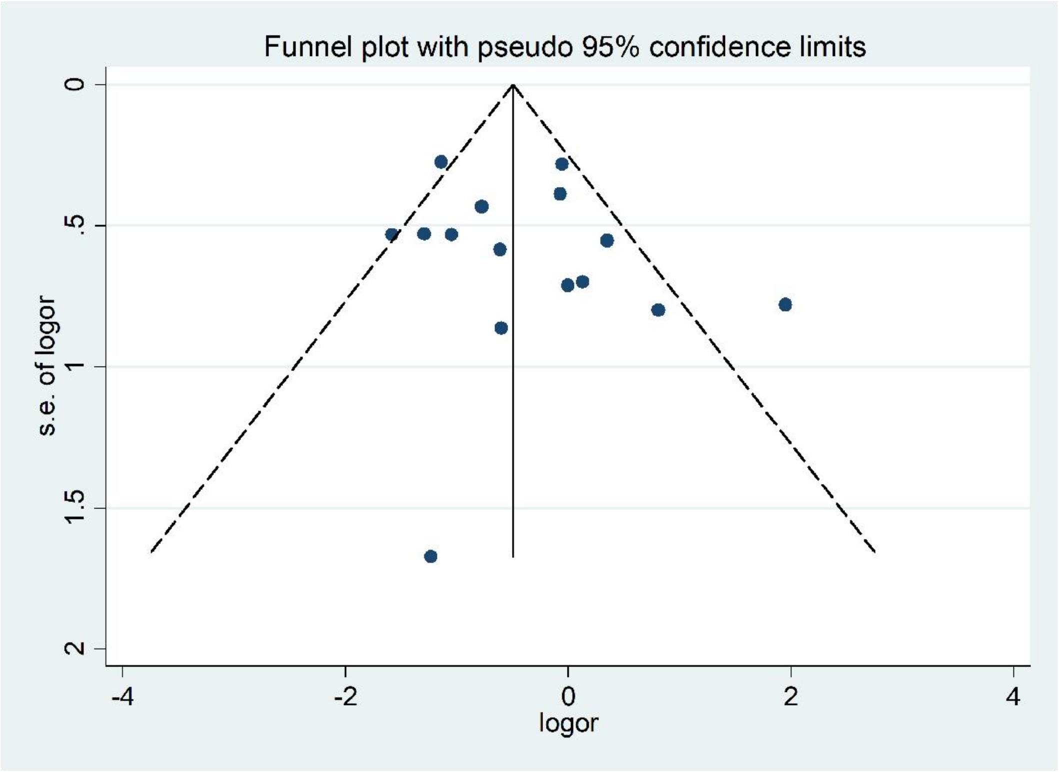

Figure S25: Funnel plot of differences in the distribution (unilateral lung) of lung lesions on chest images between COVID-19 patients and influenza patients

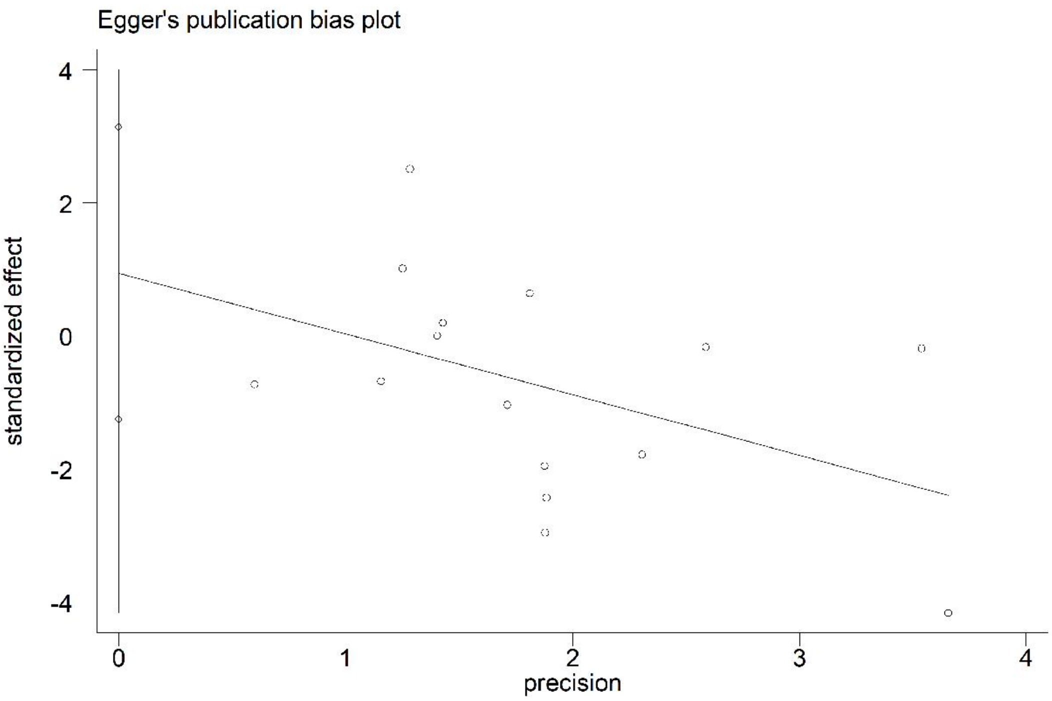

Figure S26: Egger’ s publication bias plot of differences in the distribution (unilateral lung) of lung lesions on chest images between COVID-19 patients and influenza patients (p=0.371)

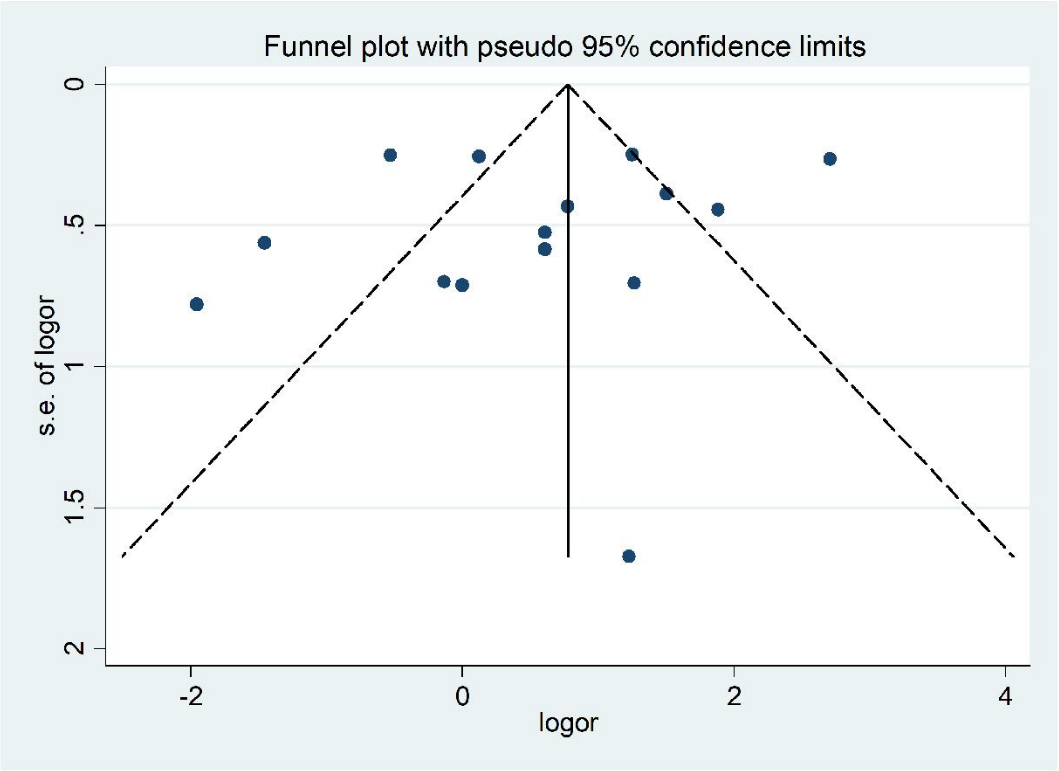

Figure S27: Funnel plot of differences in the distribution (bilateral lung) of lung lesions on chest images between COVID-19 patients and influenza patients

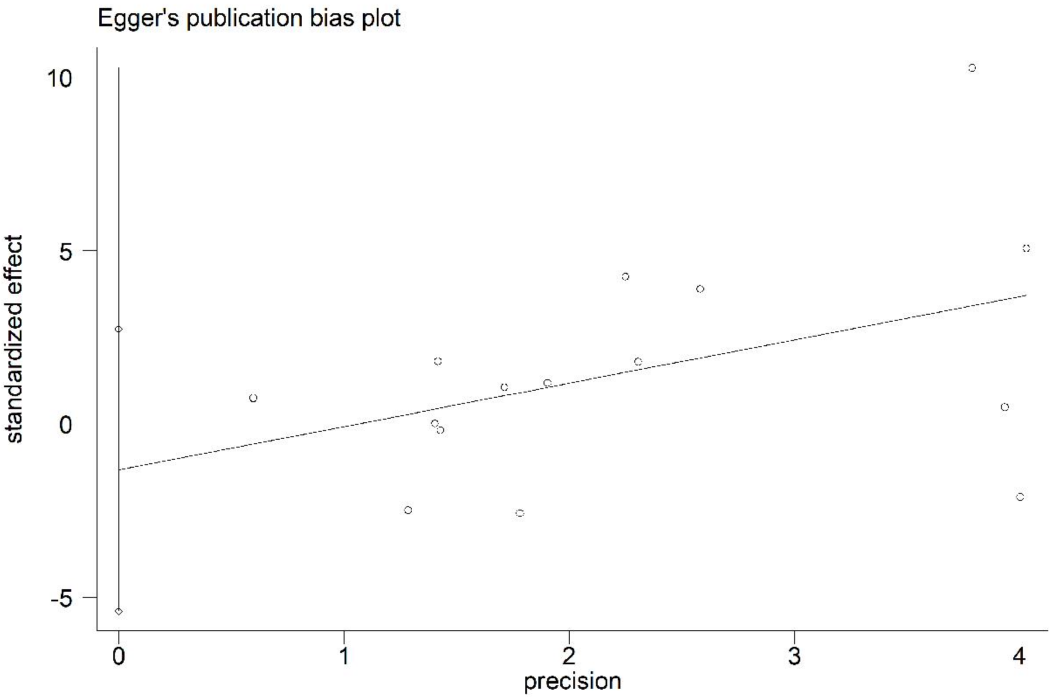

Figure S28: Egger' s publication bias plot of differences in the distribution (bilateral lung) of lung lesions on chest images between COVID-19 patients and influenza patients (p=0.491)

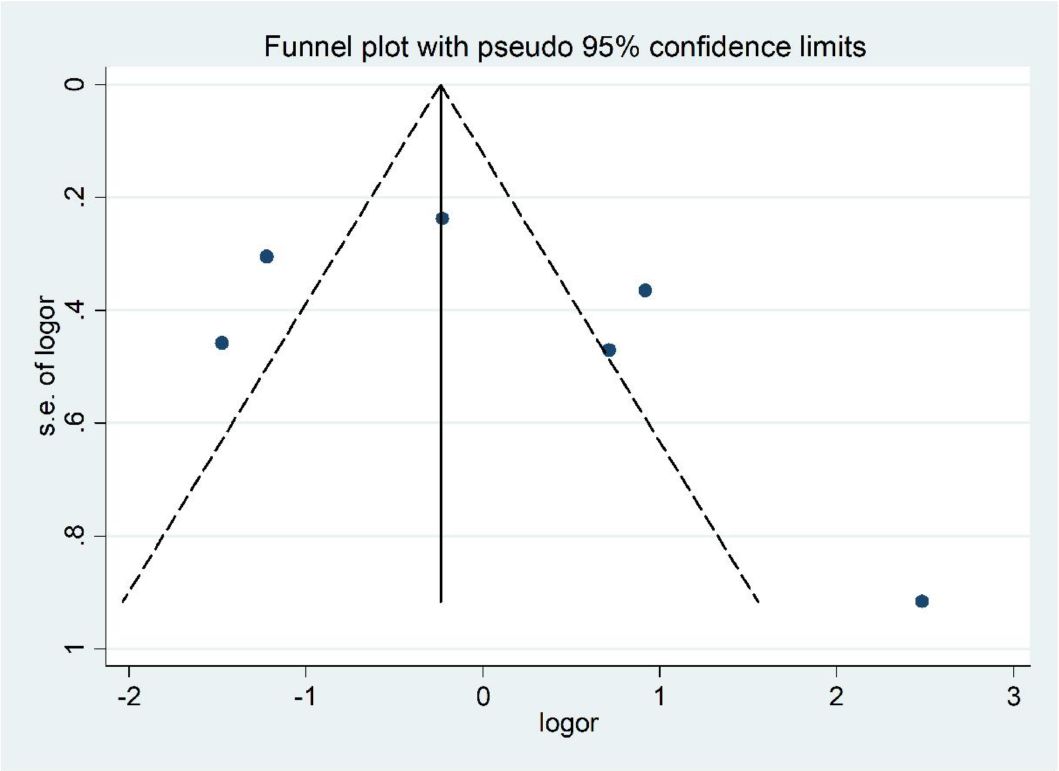

Figure S29: Funnel plot of differences in the distribution (right upper lobe) of lung lesions on chest images between COVID-19 patients and influenza patients

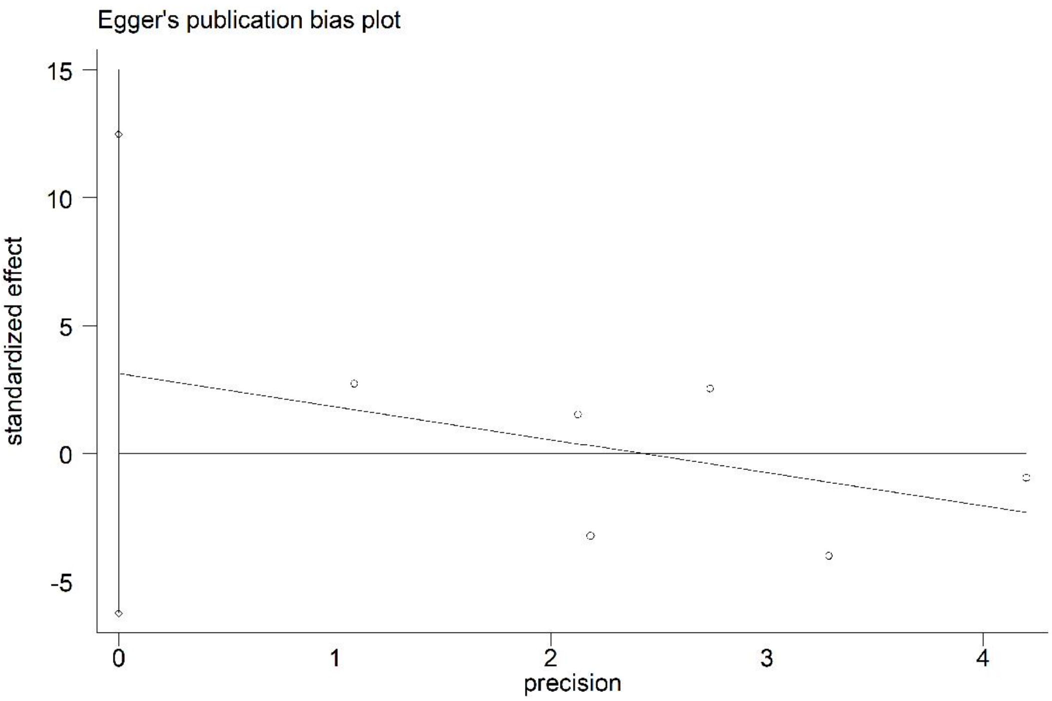

Figure S30: Egger’s publication bias plot of differences in the distribution (right upper lobe) of lung lesions on chest images between COVID-19 patients and influenza patients (p=0.407)

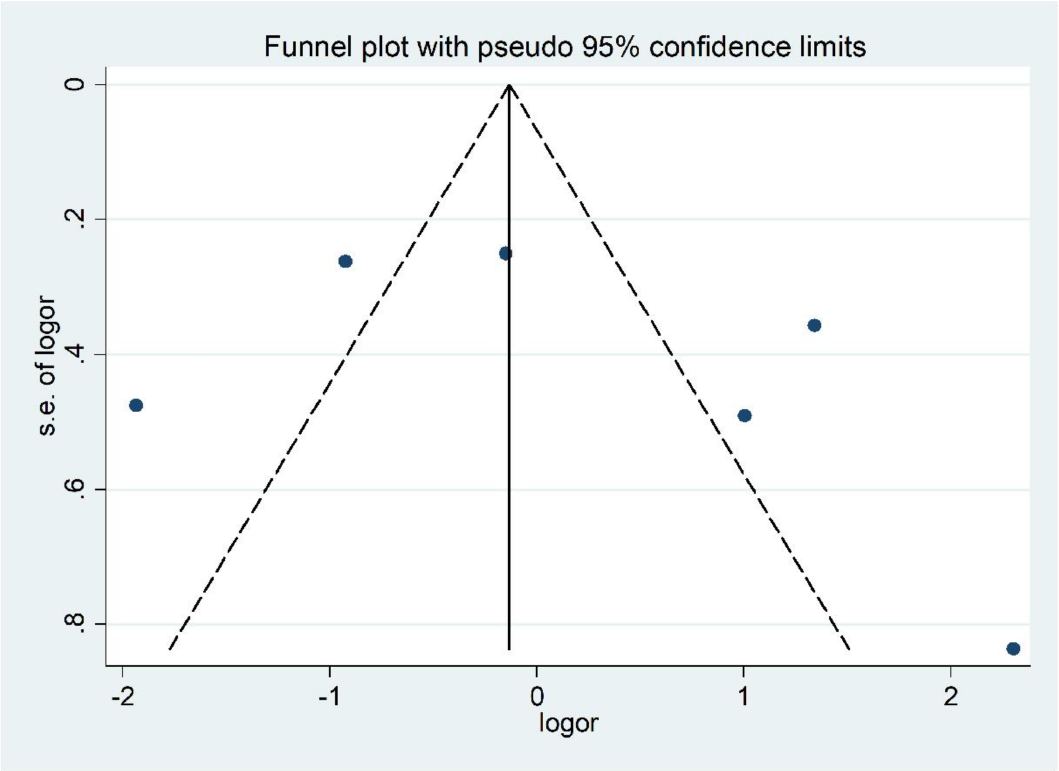

Figure S31: Funnel plot of differences in the distribution (right middle lobe) of lung lesions on chest images between COVID-19 patients and influenza patients

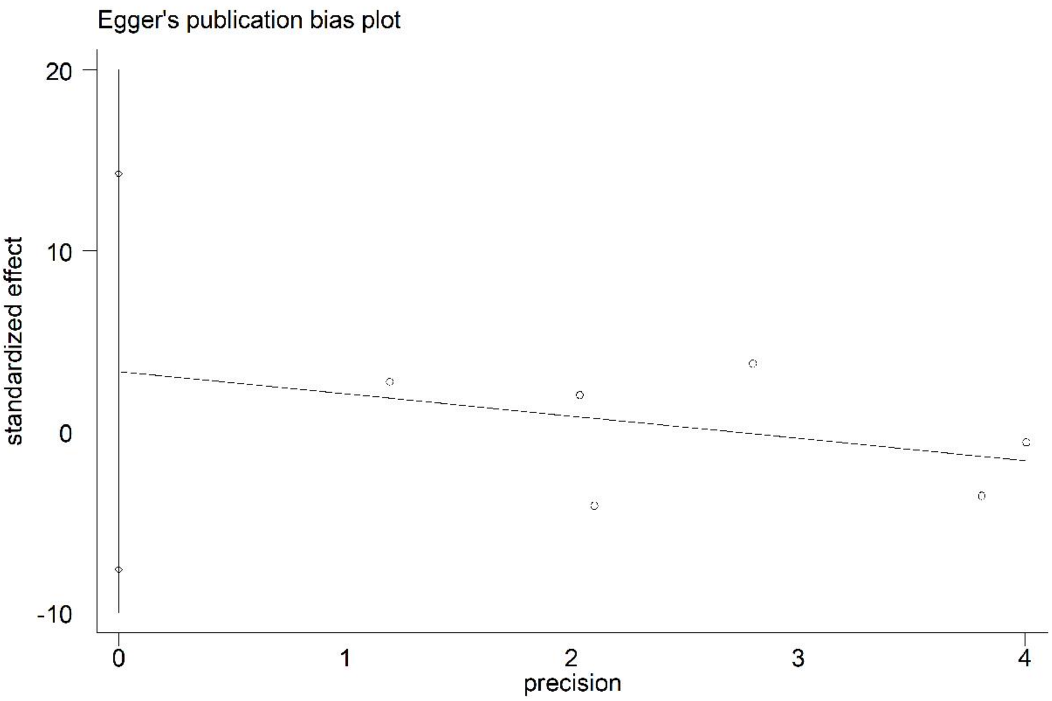

Figure S32: Egger's publication bias plot of differences in the distribution (right middle lobe) of lung lesions on chest images between COVID-19 patients and influenza patients (p=0.446)

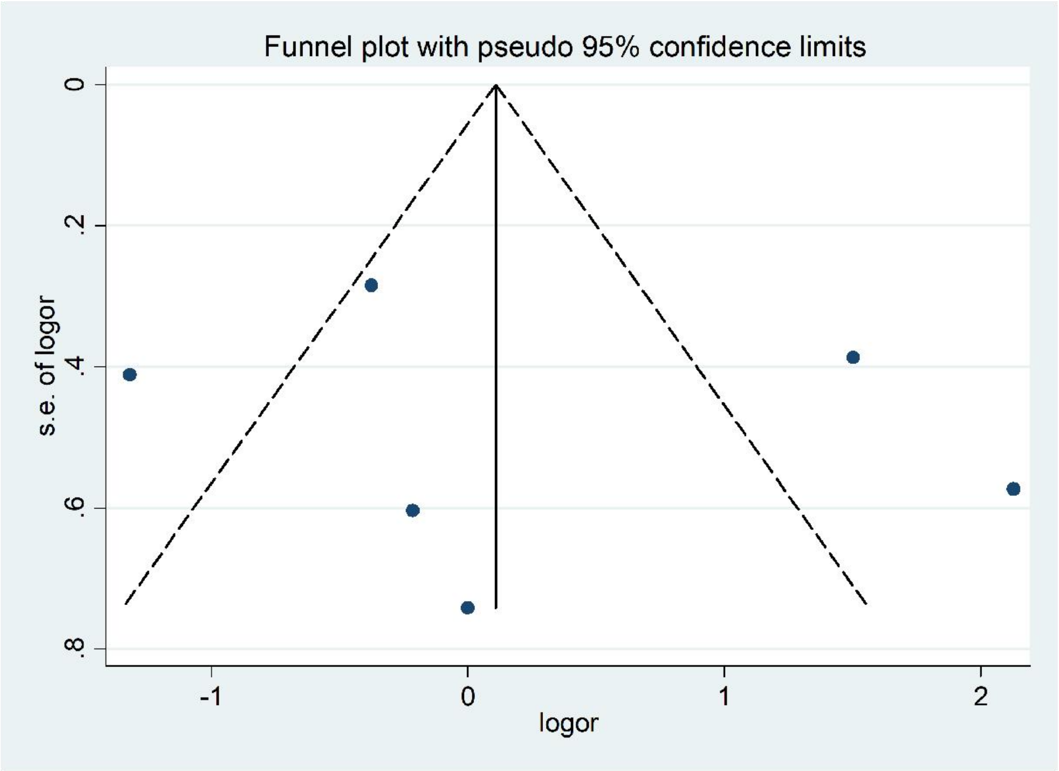

Figure S33: Funnel plot of differences in the distribution (right lower lobe) of lung lesions on chest images between COVID-19 patients and influenza patients

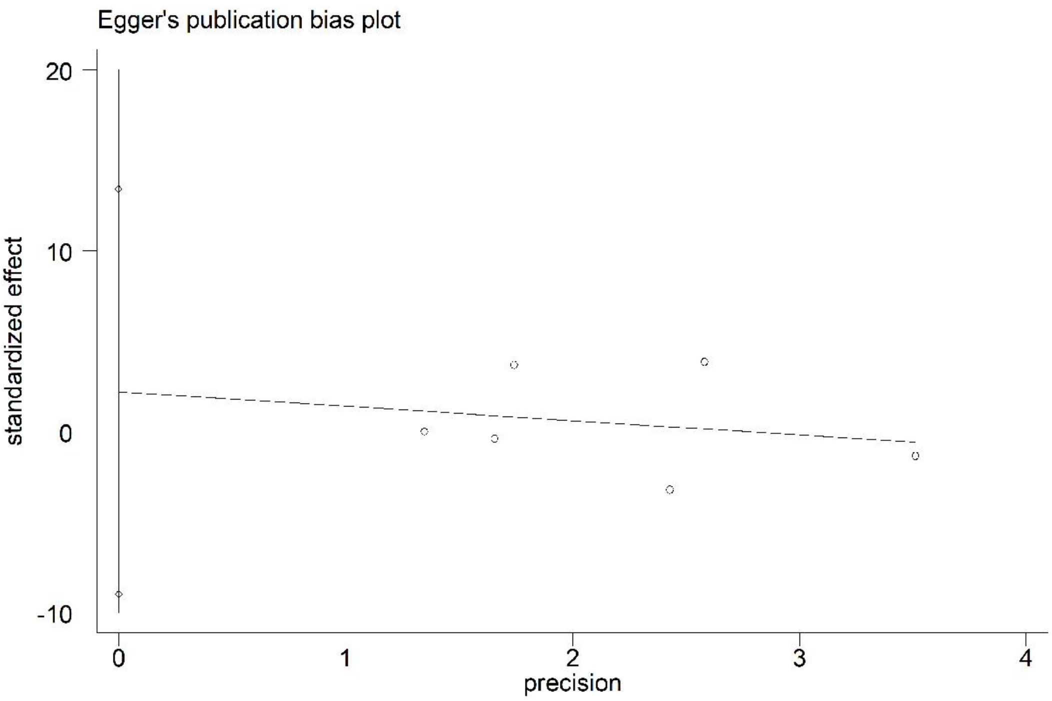

Figure S34: Egger’s publication bias plot of differences in the distribution (right lower lobe) of lung lesions on chest images between COVID-19 patients and influenza patients (p=0.613)

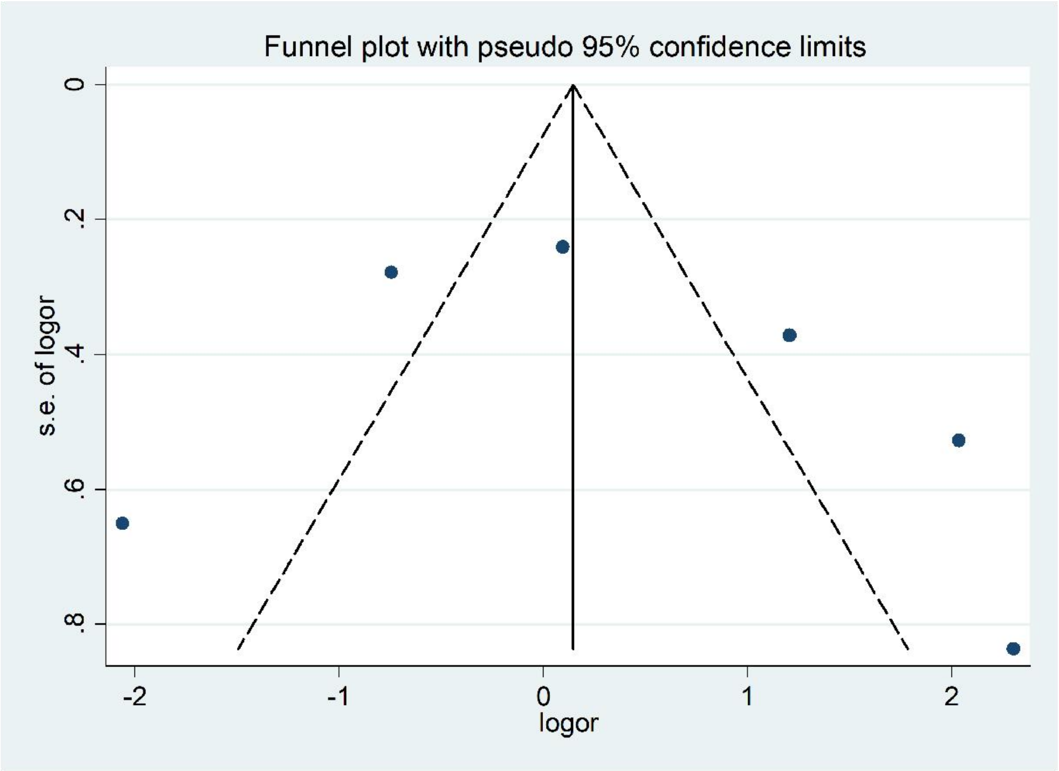

Figure S35: Funnel plot of differences in the distribution (left upper lobe) of lung lesions on chest images between COVID-19 patients and influenza patients

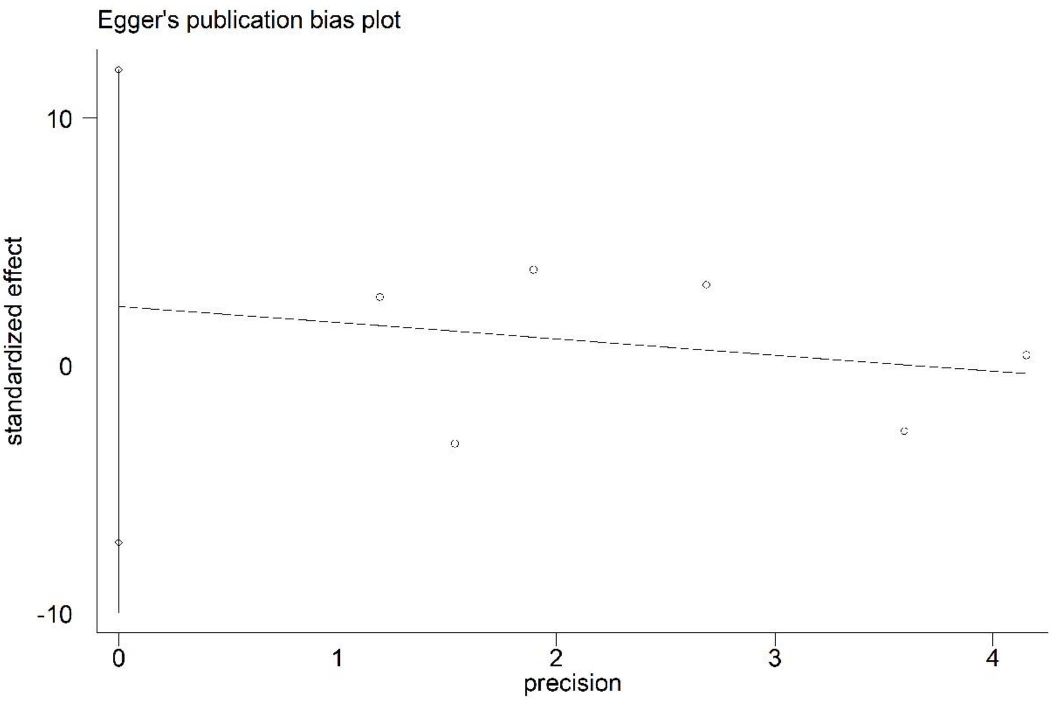

Figure S36: Egger’s publication bias plot of differences in the distribution (left upper lobe) of lung lesions on chest images between COVID-19 patients and influenza patients (p=0.526)

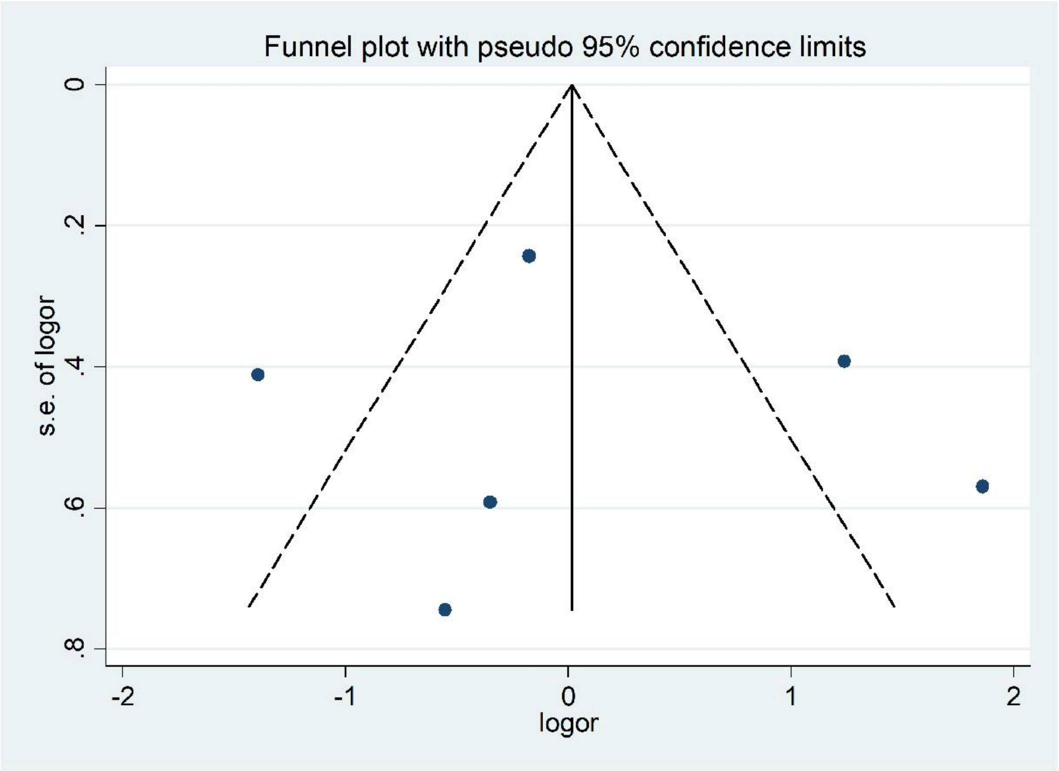

Figure S37: Funnel plot of differences in the distribution (left lower lobe) of lung lesions on chest images between COVID-19 patients and influenza patients

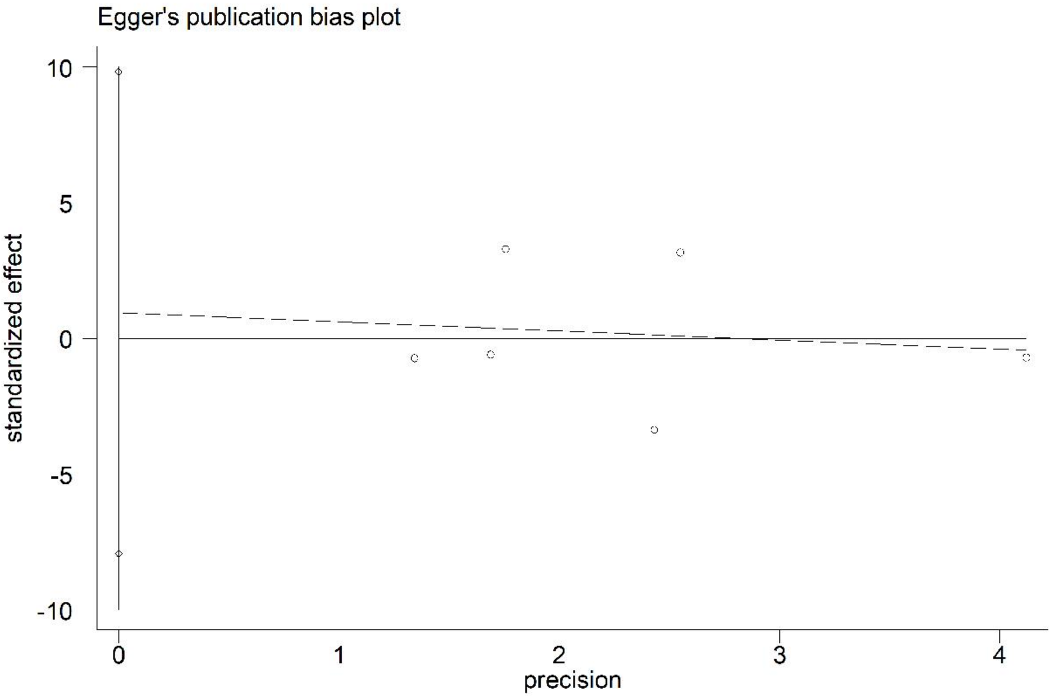

Figure S38: Egger's publication bias plot of differences in the distribution (left lower lobe) of lung lesions on chest images between COVID-19 patients and influenza patients (p=0.780)

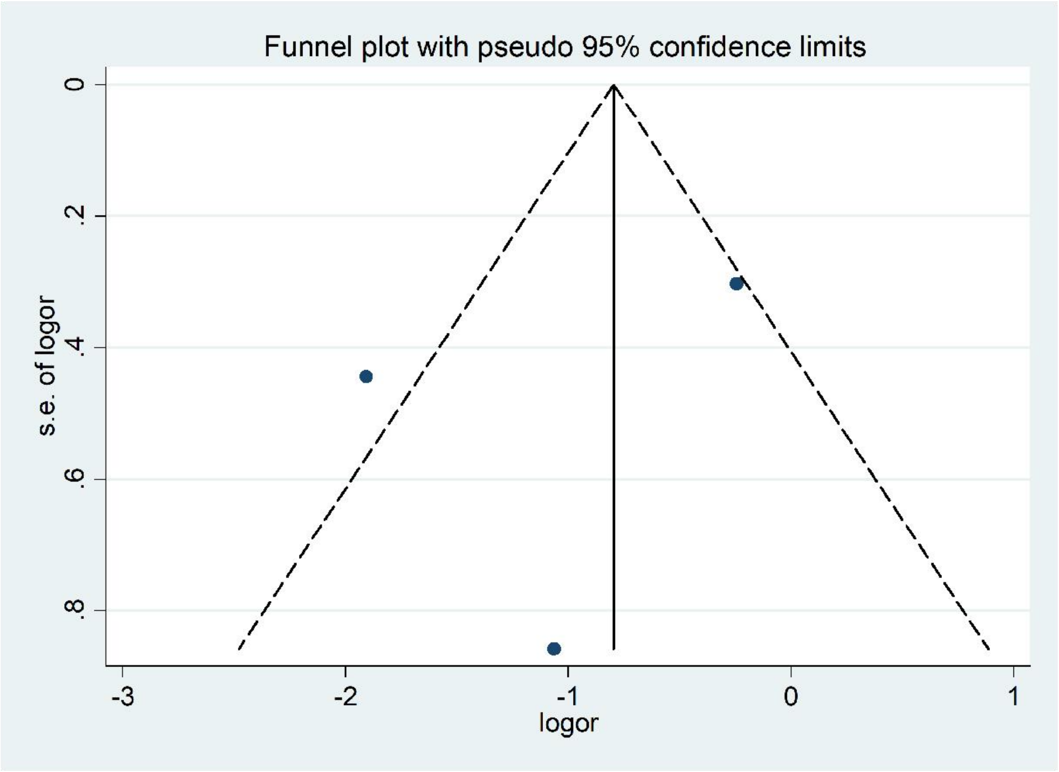

Figure S39: Funnel plot of differences in the distribution (0-1 lobes involved) of lung lesions on chest images between COVID-19 patients and influenza patients

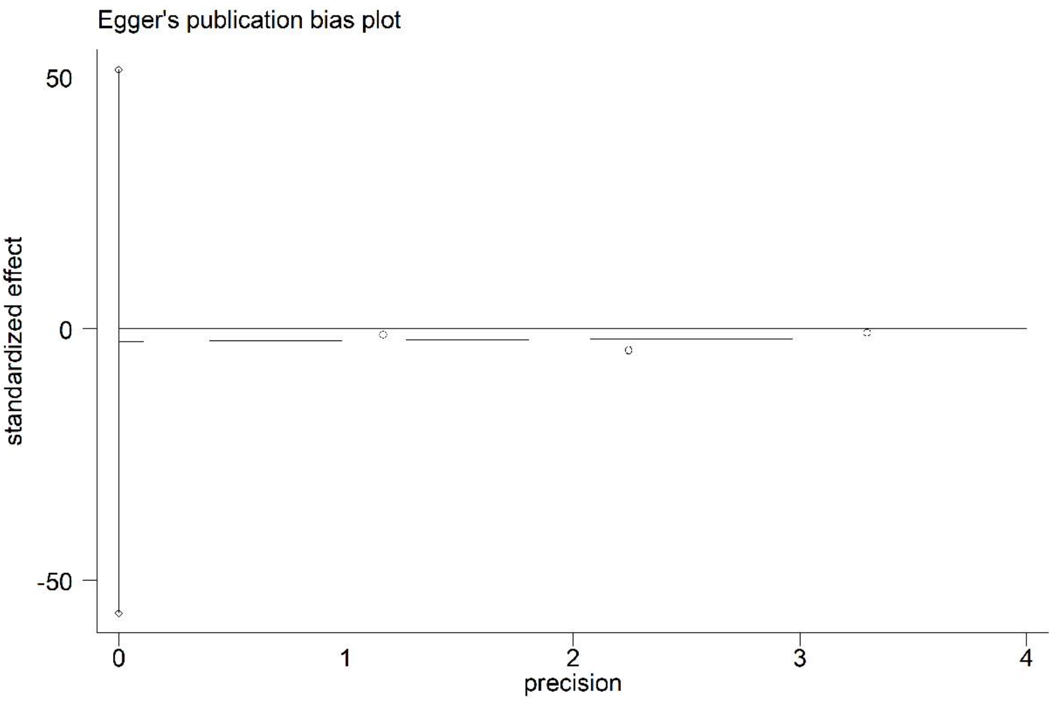

Figure S40: Egger's publication bias plot of differences in the distribution (0-1 lobes involved) of lung lesions on chest images between COVID-19 patients and influenza patients (p=0.658)

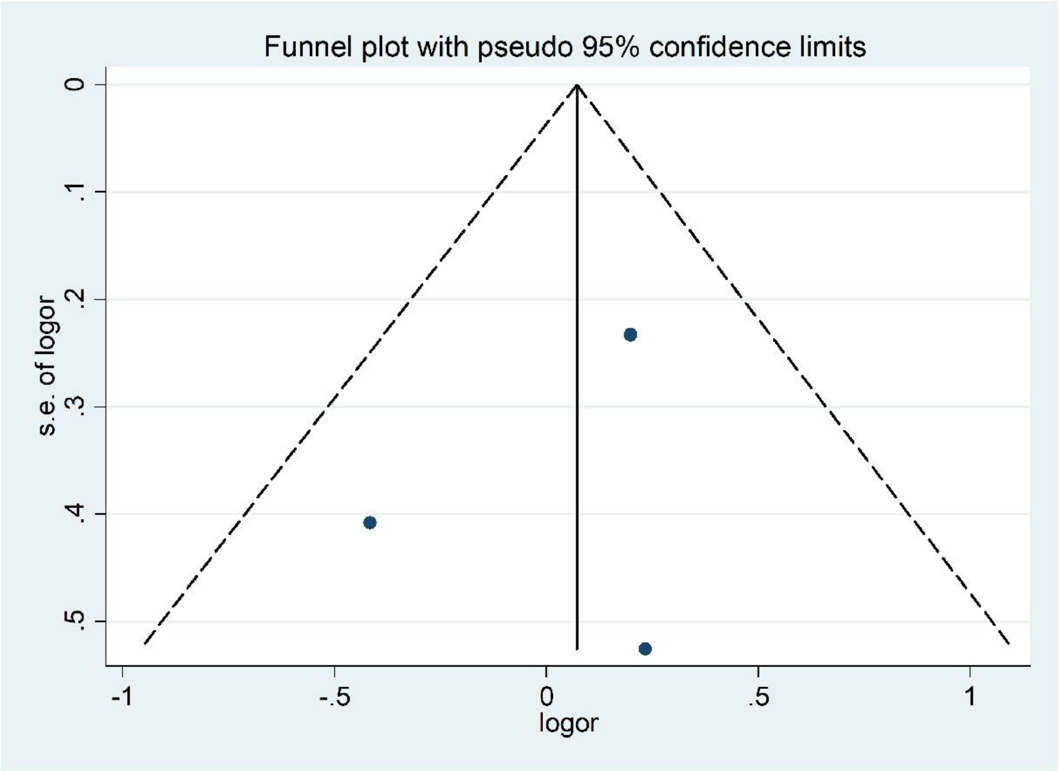

Figure S41: Funnel plot of differences in the distribution (2-3 lobes involved) of lung lesions on chest images between COVID-19 patients and influenza patients

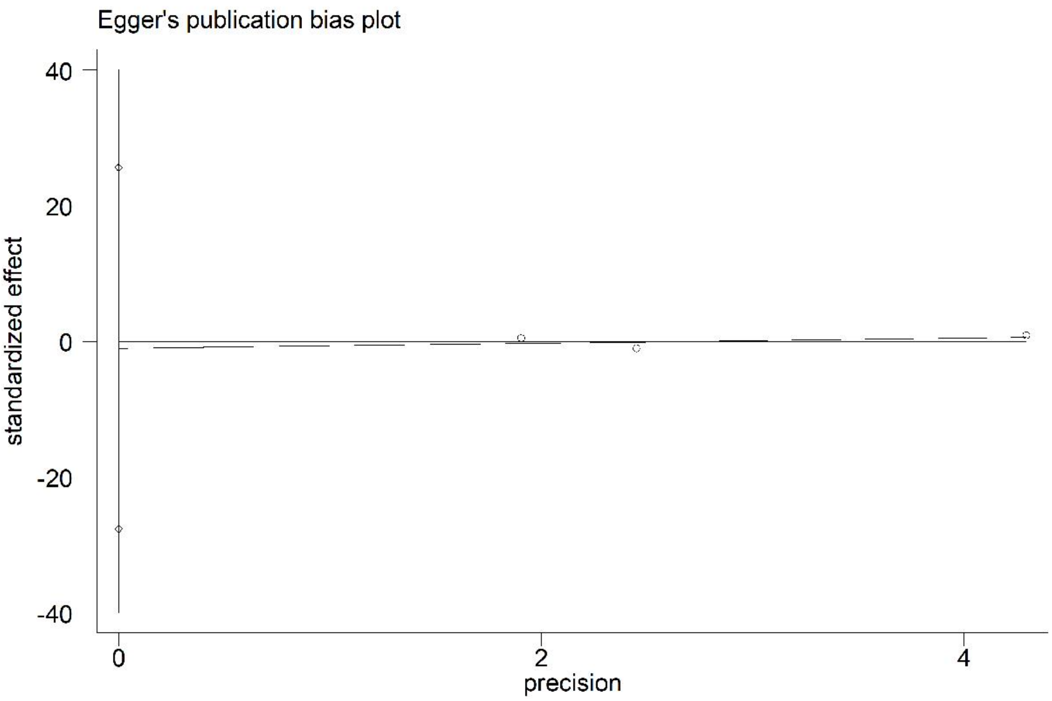

Figure S42: Egger' s publication bias plot of differences in the distribution (2-3 lobes involved) of lung lesions on chest images between COVID-19 patients and influenza patients (p=0.712)

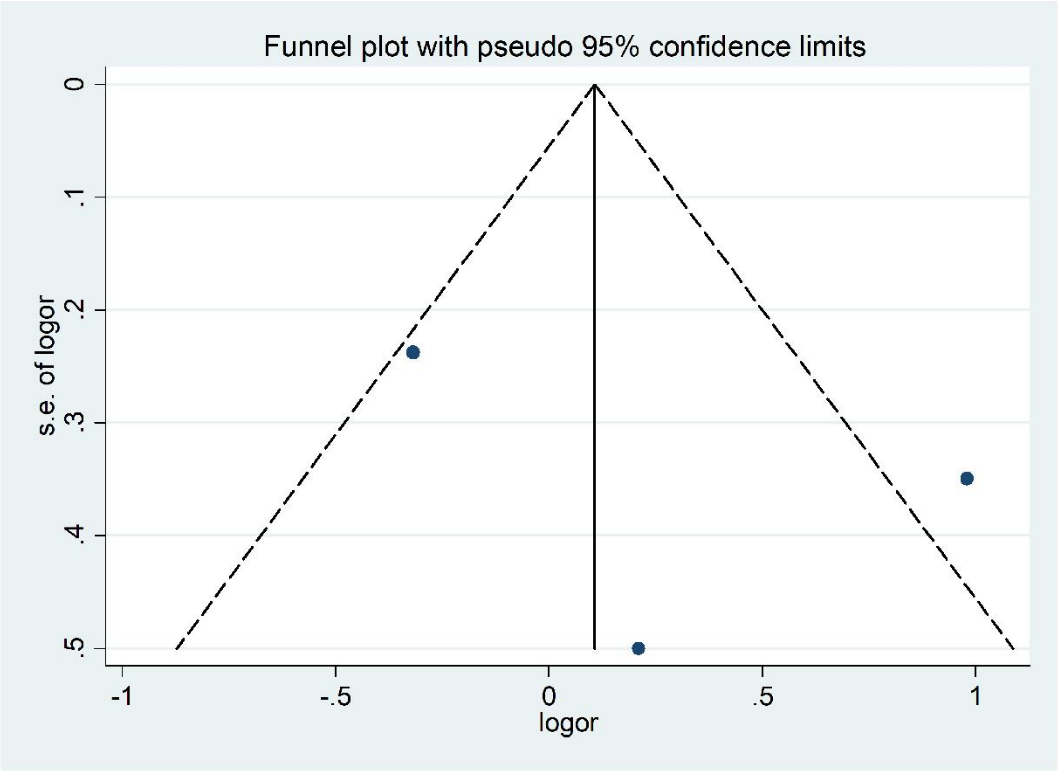

Figure S43: Funnel plot of differences in the distribution (4-5 lobes involved) of lung lesions on chest images between COVID-19 patients and influenza patients

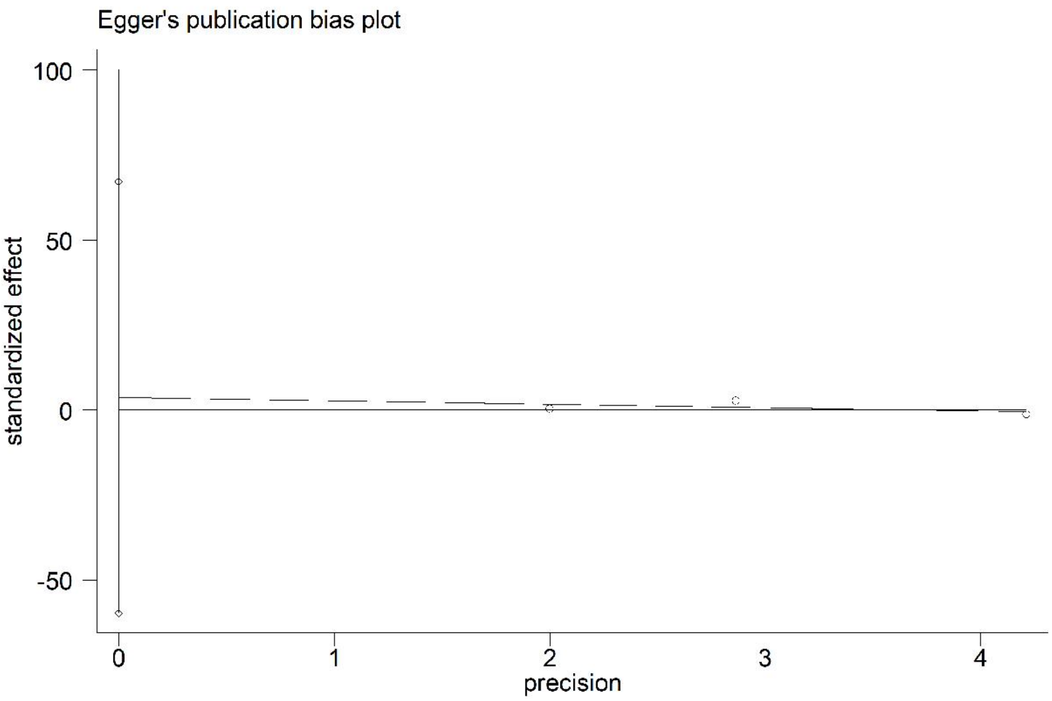

Figure S44: Egger’s publication bias plot of differences in the distribution (4-5 lobes involved) of lung lesions on chest images between COVID-19 patients and influenza patients (p=0.598)

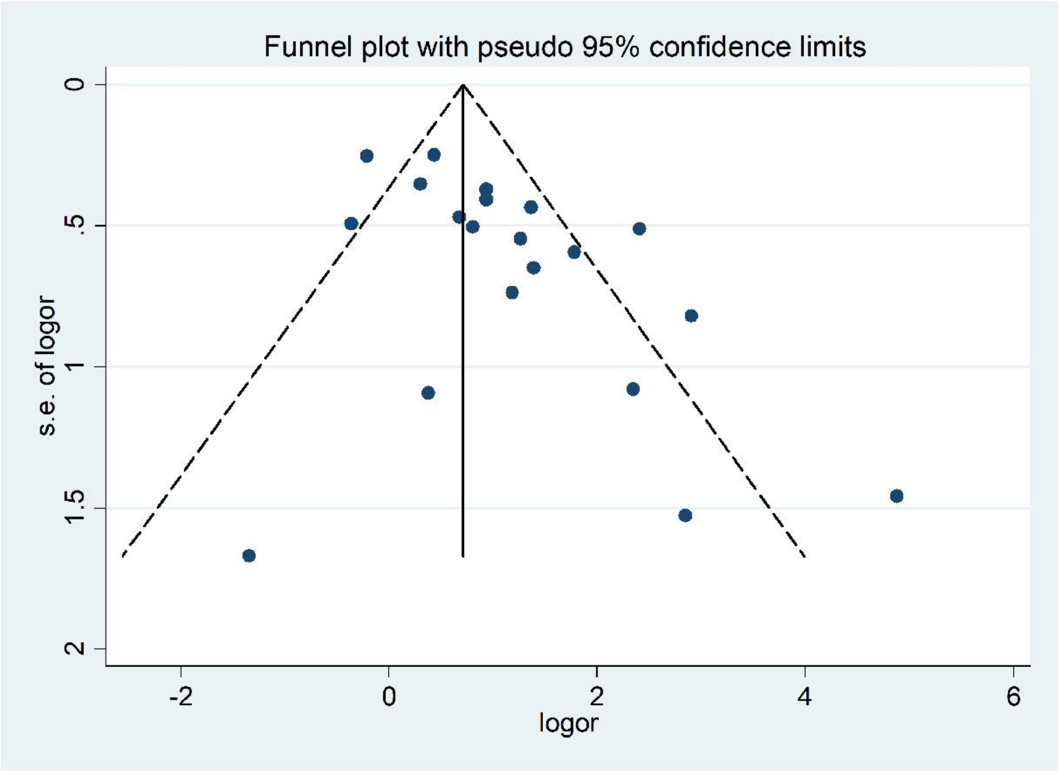

Figure S45: Funnel plot of differences in the manifestation (ground-glass opacities) of lung lesions on chest images between COVID-19 patients and influenza patients

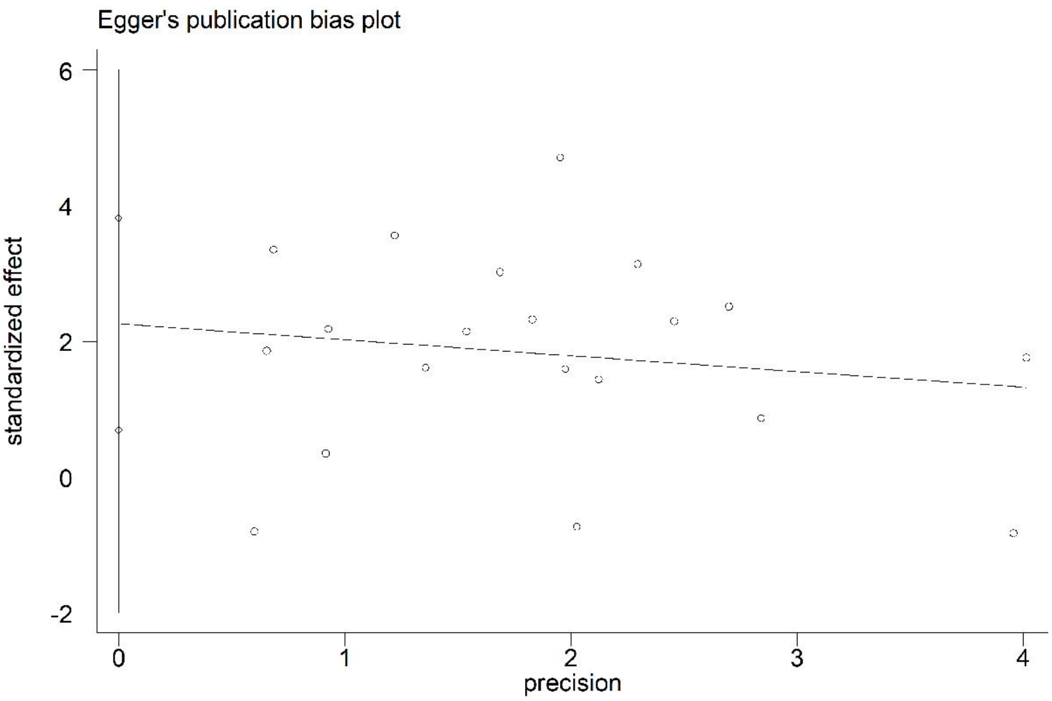

Figure S46: Egger's publication bias plot of differences in the manifestation (ground-glass opacities) of lung lesions on chest images between COVID-19 patients and influenza patients (p=0.007)

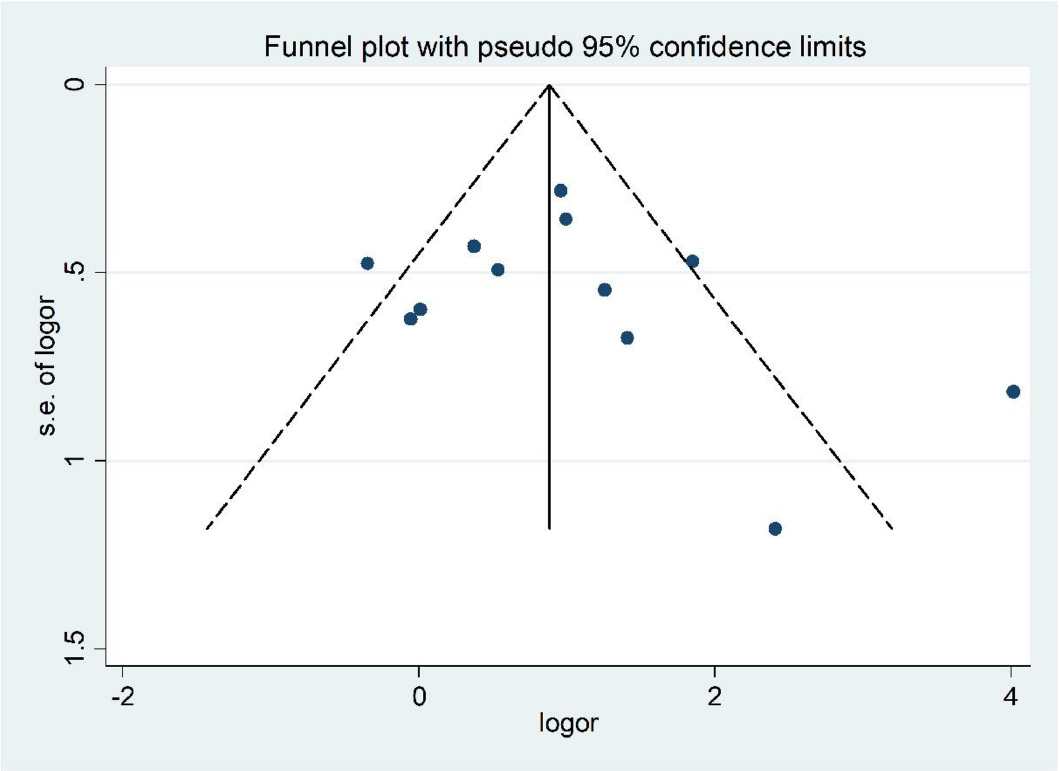

Figure S47: Funnel plot of differences in the manifestation (crazy-paving patterns) of lung lesions on chest images between COVID-19 patients and influenza patients

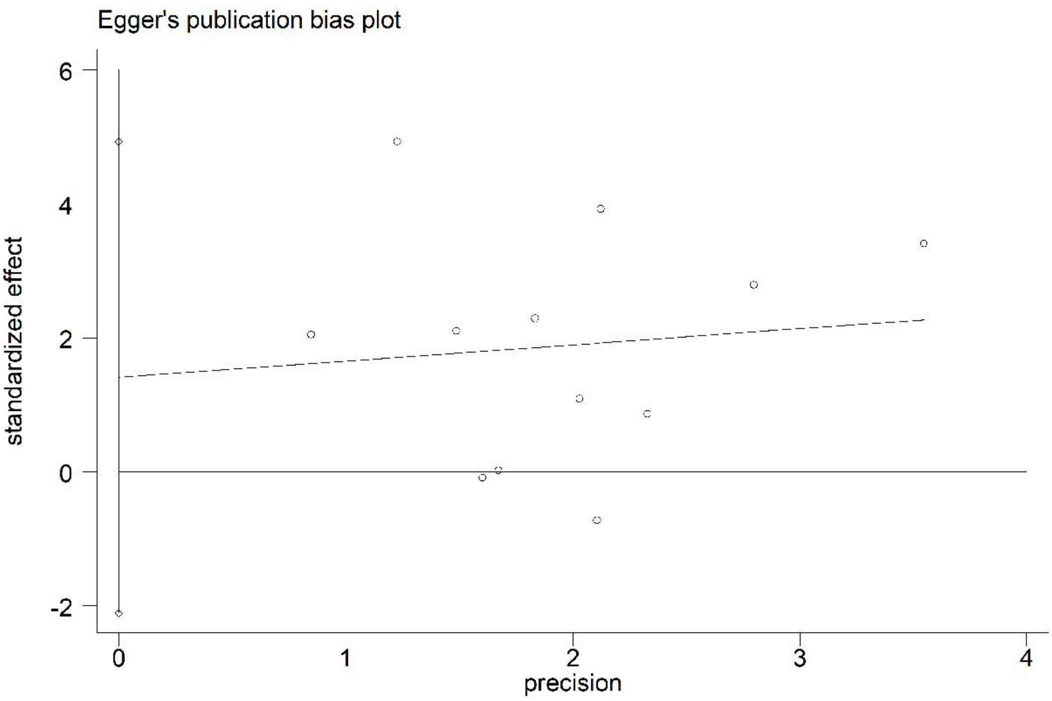

Figure S48: Egger's publication bias plot of differences in the manifestation (crazy-paving patterns) of lung lesions on chest images between COVID-19 patients and influenza patients (p=0.392)

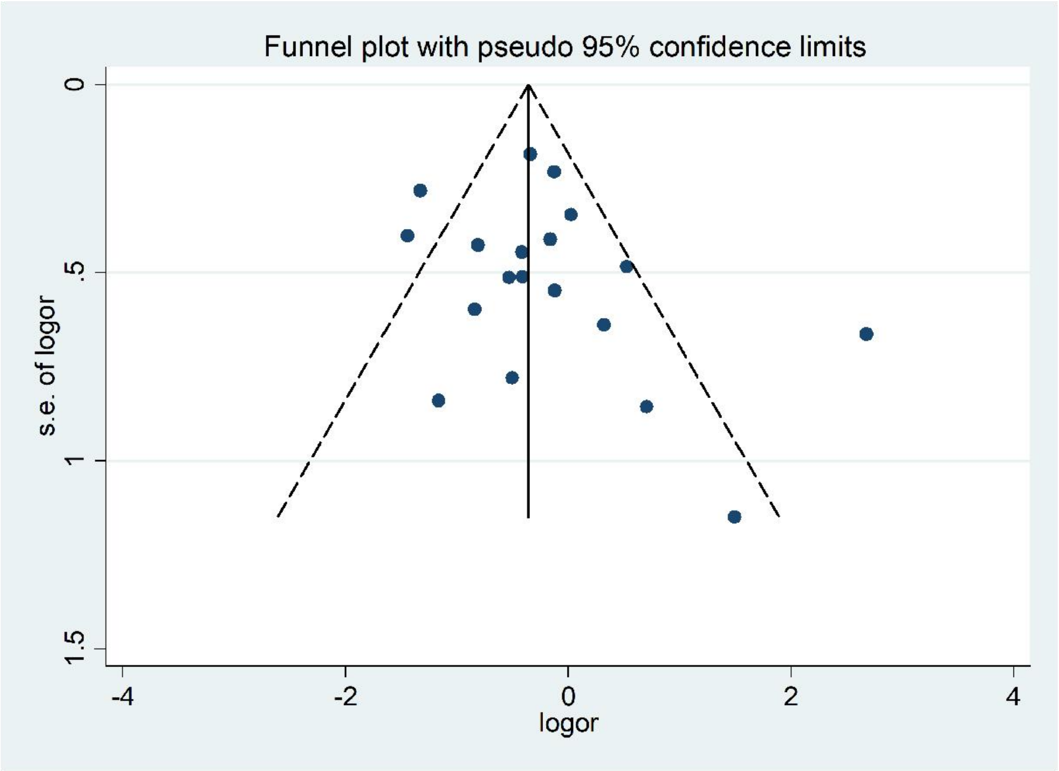

Figure S49: Funnel plot of differences in the manifestation (consolidation) of lung lesions on chest images between COVID-19 patients and influenza patients

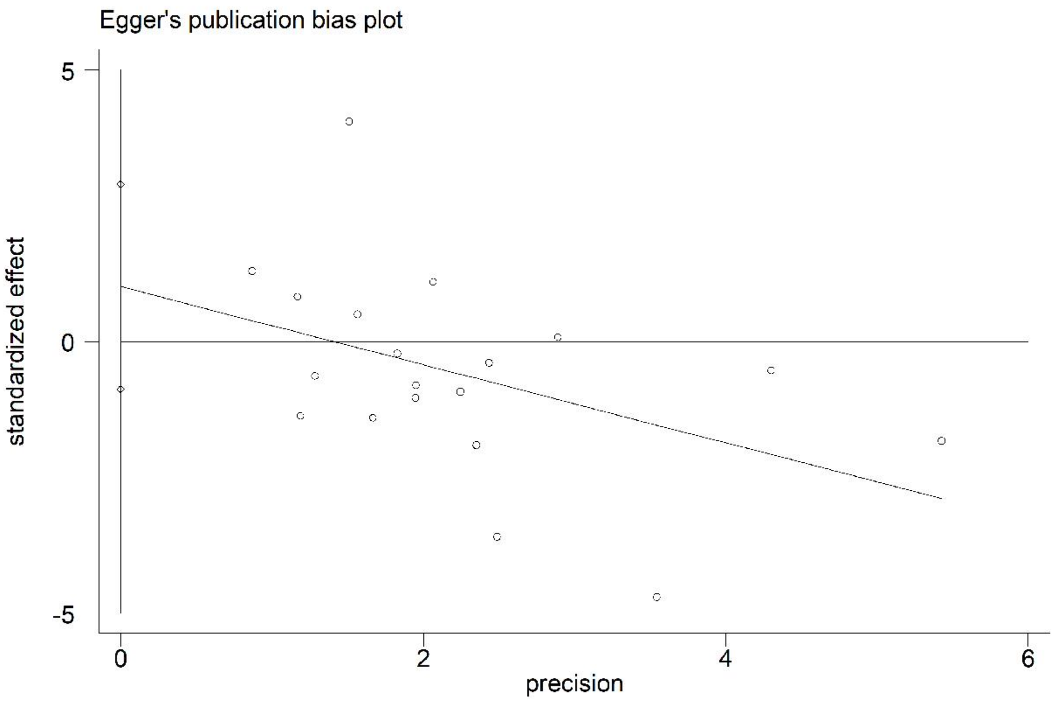

Figure S50: Egger's publication bias plot of differences in the manifestation (consolidation) of lung lesions on chest images between COVID-19 patients and influenza patients (p=0.274)

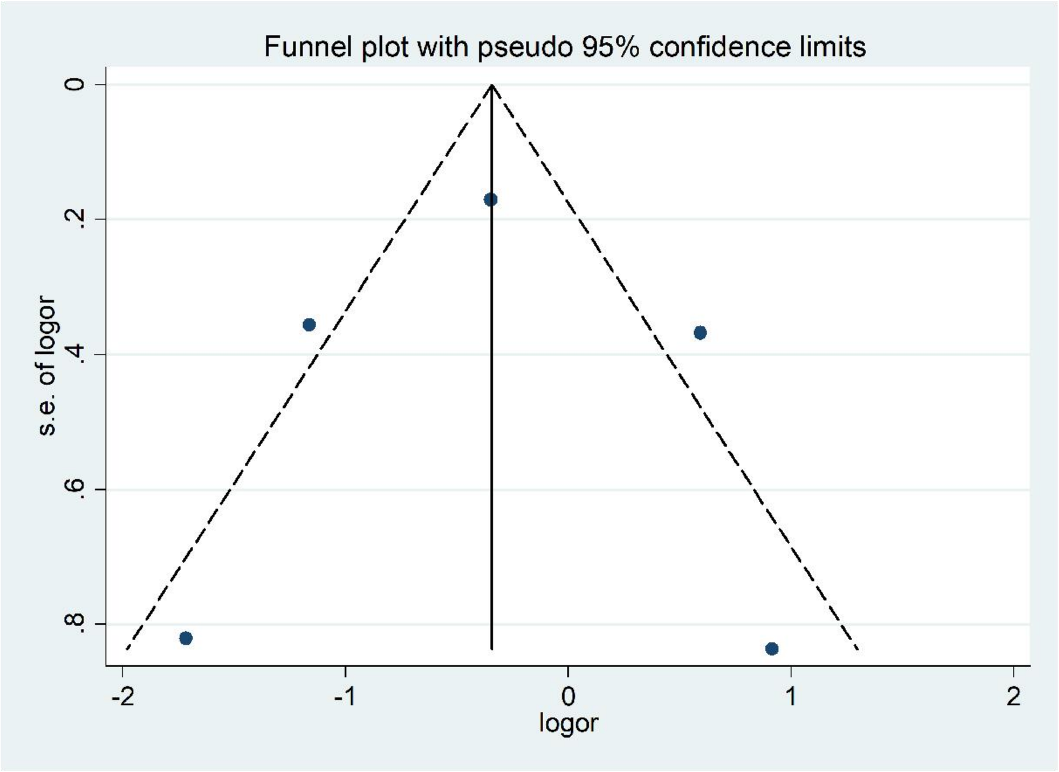

Figure S51: Funnel plot of differences in the manifestation (nodules with non-tree-in-bud) of lung lesions on chest images between COVID-19 patients and influenza patients

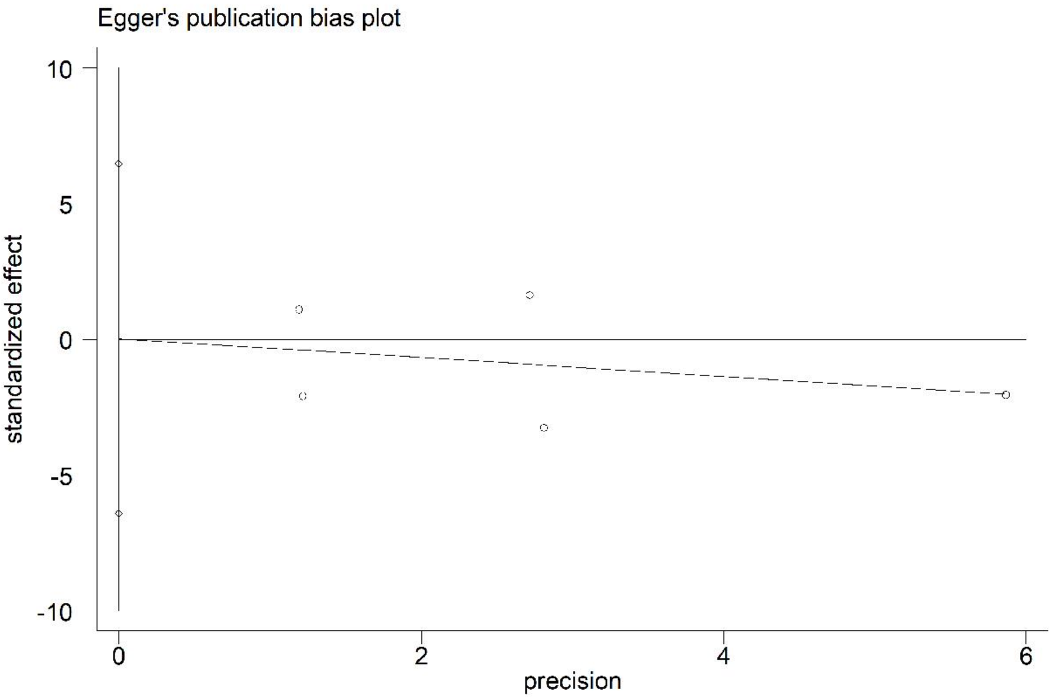

Figure S52: Egger's publication bias plot of differences in the manifestation (nodules with non-tree-in-bud) of lung lesions on chest images between COVID-19 patients and influenza patients (p=0.990)

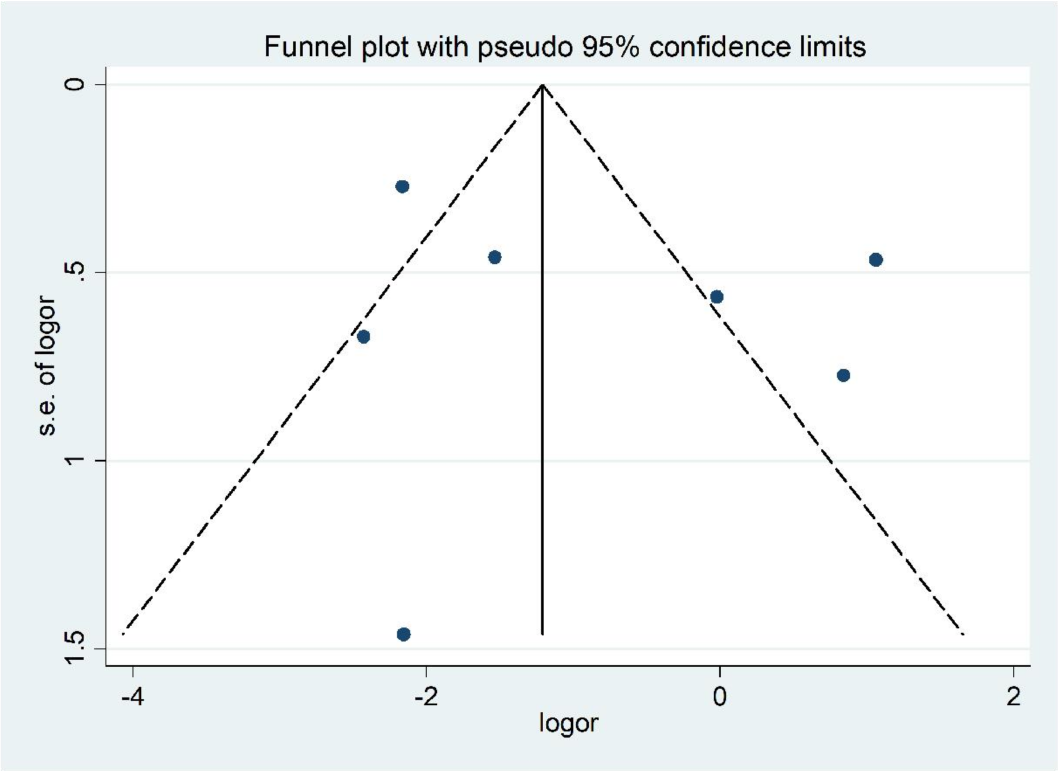

Figure S53: Funnel plot of differences in the manifestation (nodules with tree-in-bud) of lung lesions on chest images between COVID-19 patients and influenza patients

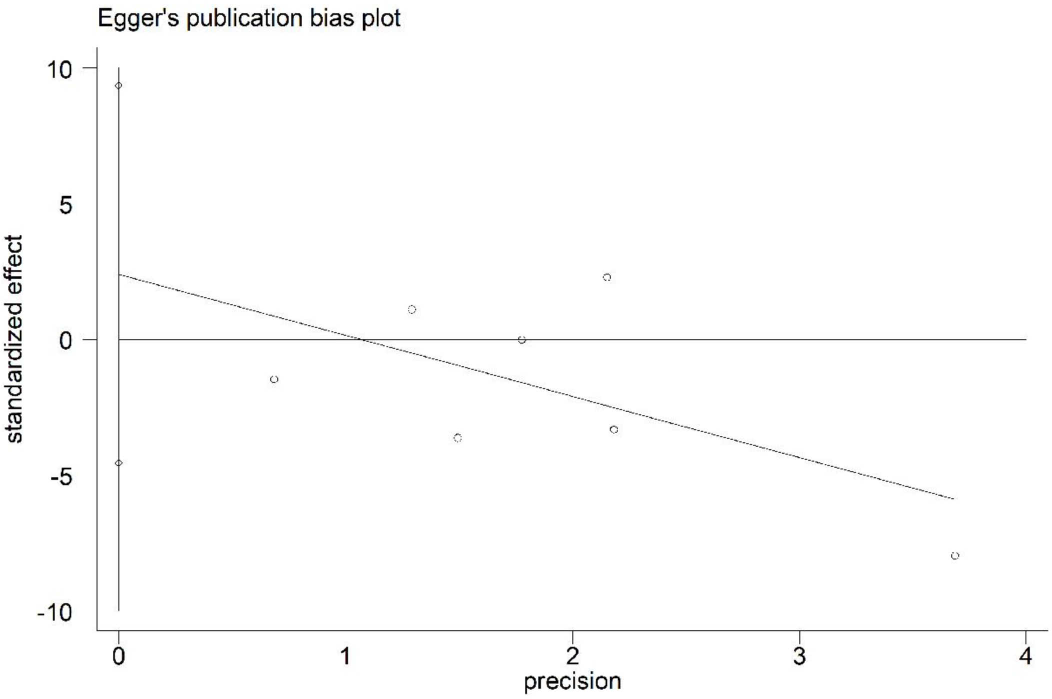

Figure S54: Egger ’ s publication bias plot of differences in the manifestation (nodules with tree-in-bud) of lung lesions on chest images between COVID-19 patients and influenza patients (p=0.416)

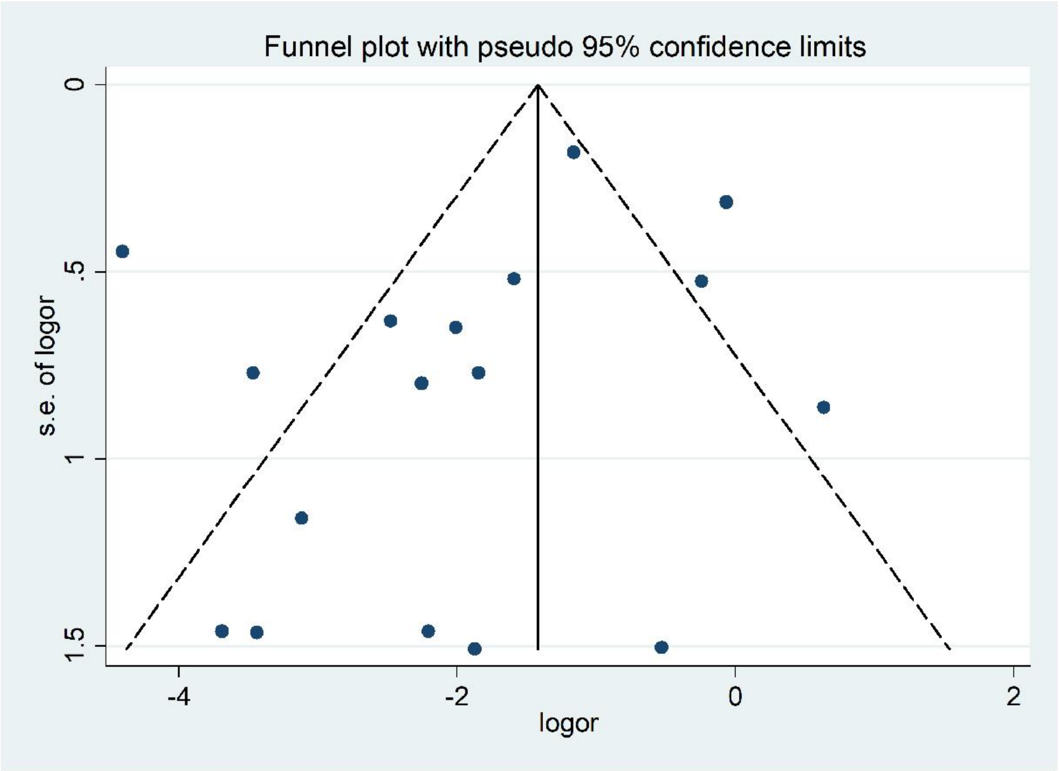

Figure S55: Funnel plot of differences in the manifestation (pleural effusion) of lung lesions on chest images between COVID-19 patients and influenza patients

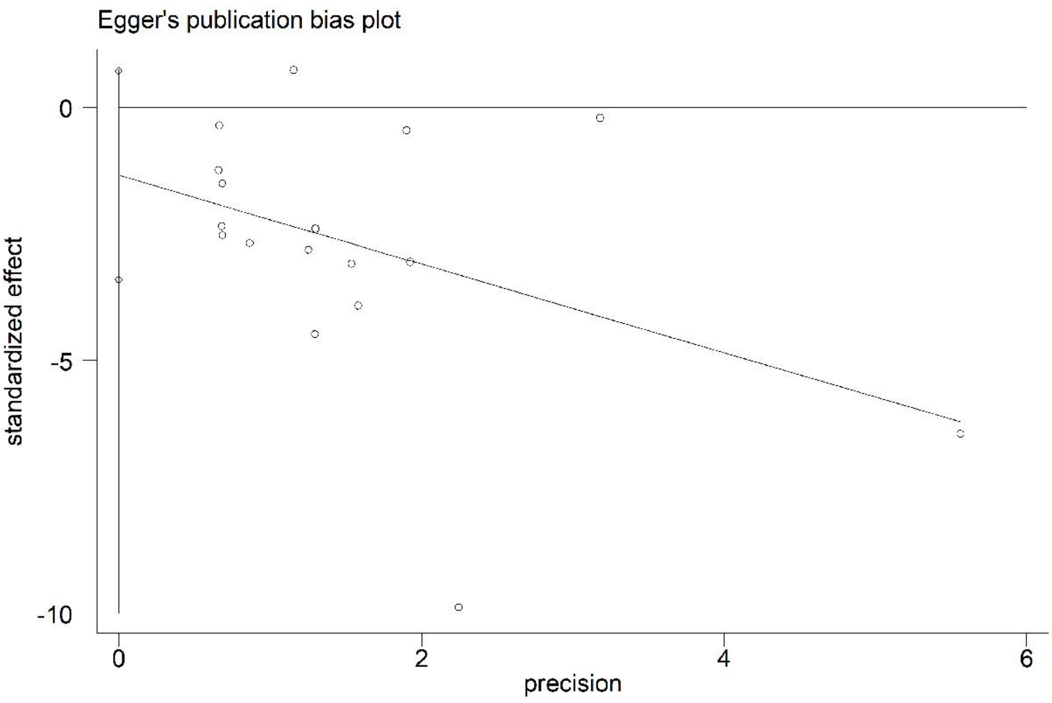

Figure S56: Egger's publication bias plot of differences in the manifestation (pleural effusion) of lung lesions on chest images between COVID-19 patients and influenza patients (p=0.183)

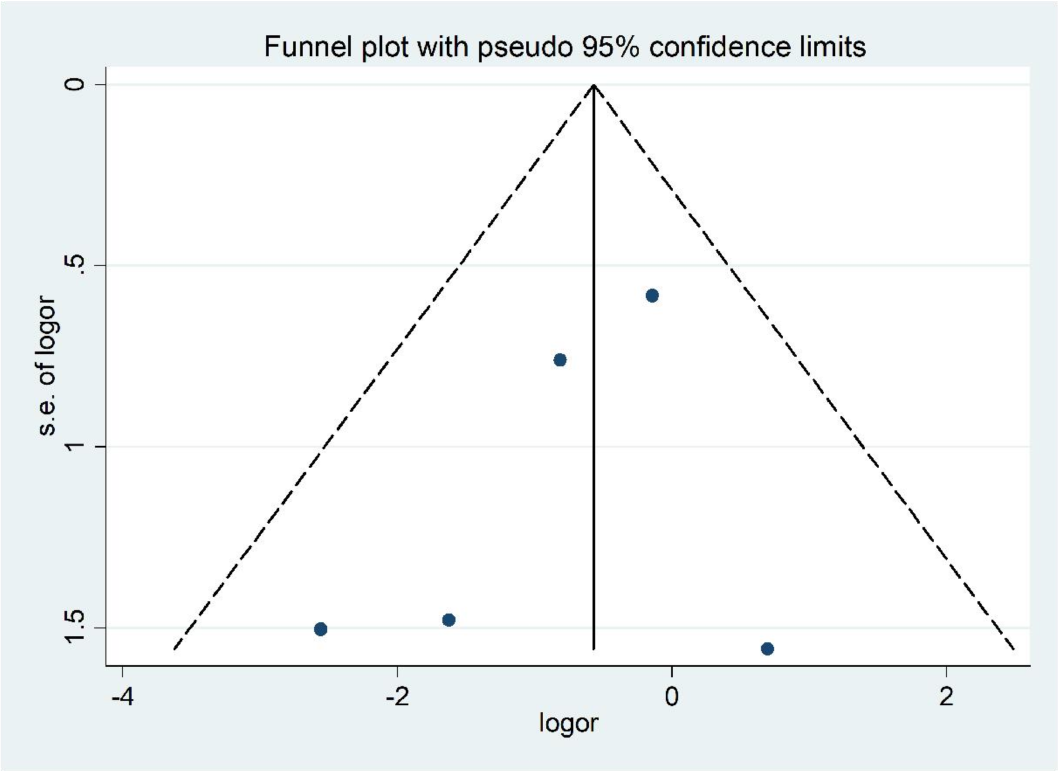

Figure S57: Funnel plot of differences in the manifestation (pericardial effusion) of lung lesions on chest images between COVID-19 patients and influenza patients

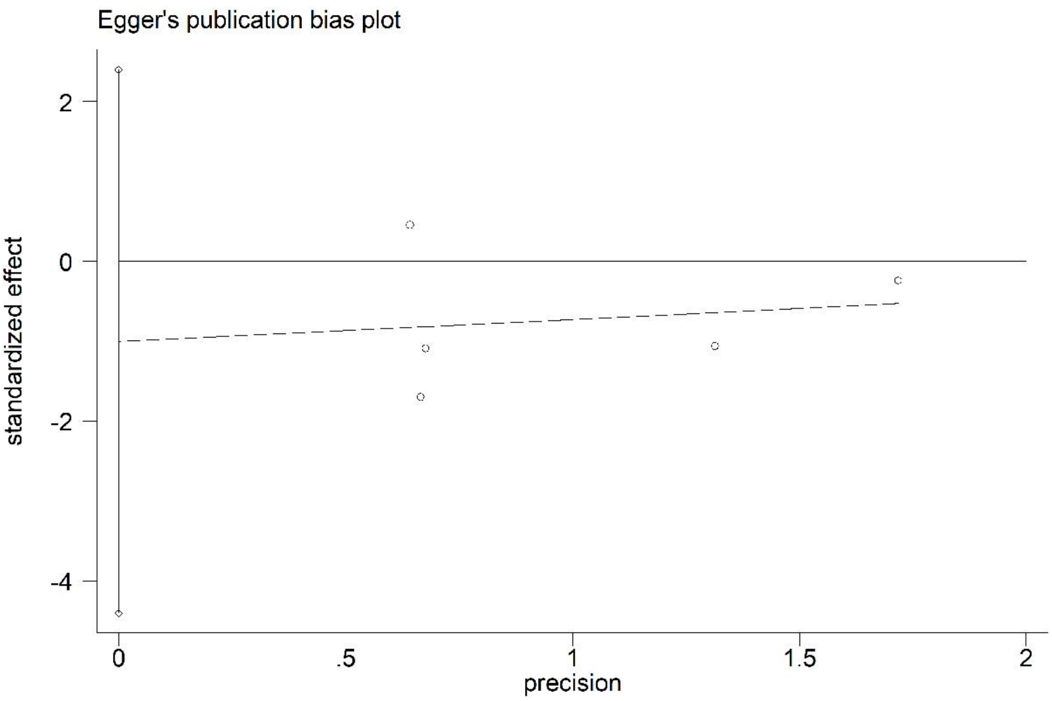

Figure S58: Egger’s publication bias plot of differences in the manifestation (pericardial effusion) of lung lesions on chest images between COVID-19 patients and influenza patients (p=0.415)

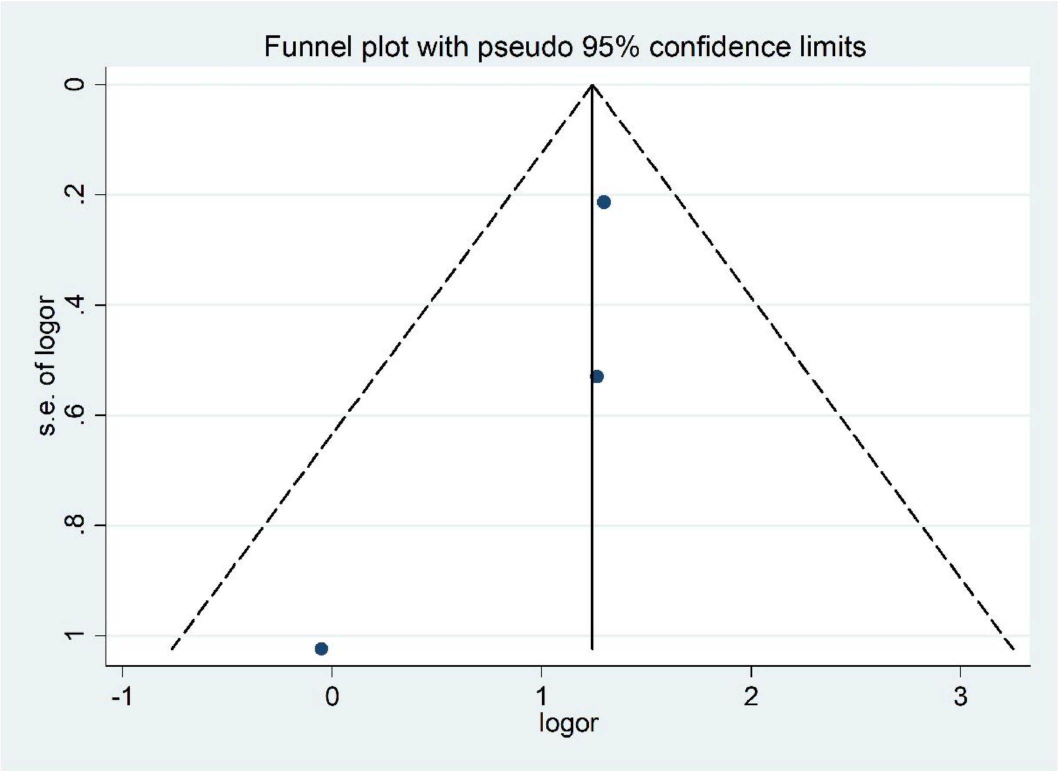

Figure S59: Funnel plot of differences in the manifestation (reverse halo signs) of lung lesions on chest images between COVID-19 patients and influenza patients

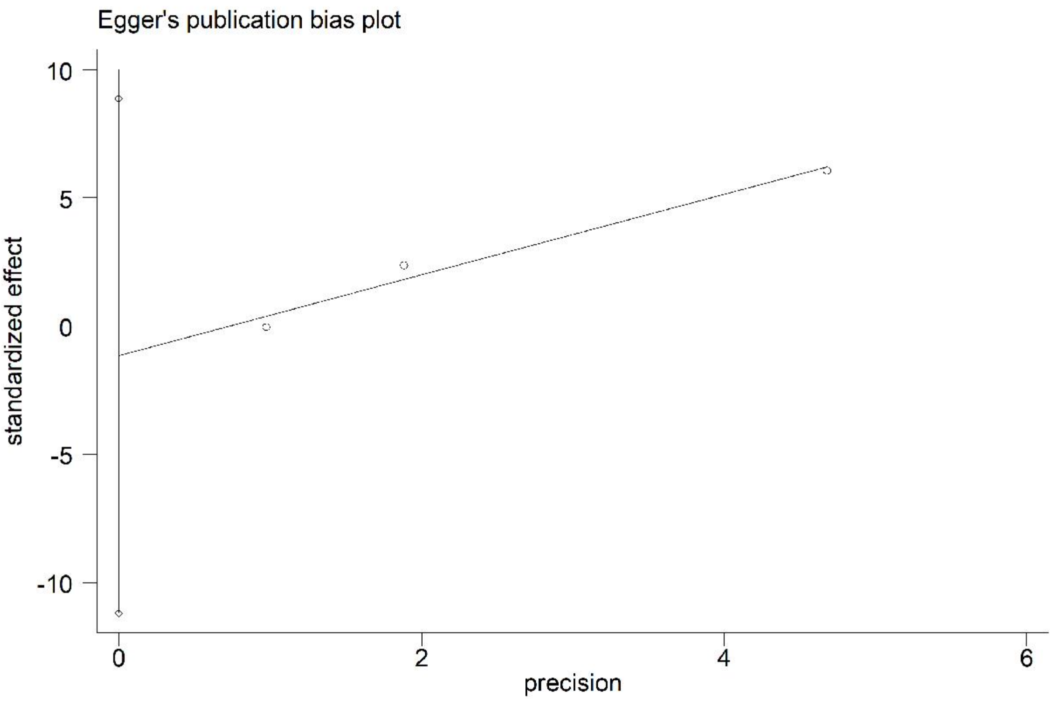

Figure S60: Egger's publication bias plot of differences in the manifestation (reverse halo signs) of lung lesions on chest images between COVID-19 patients and influenza patients (p=0.381)

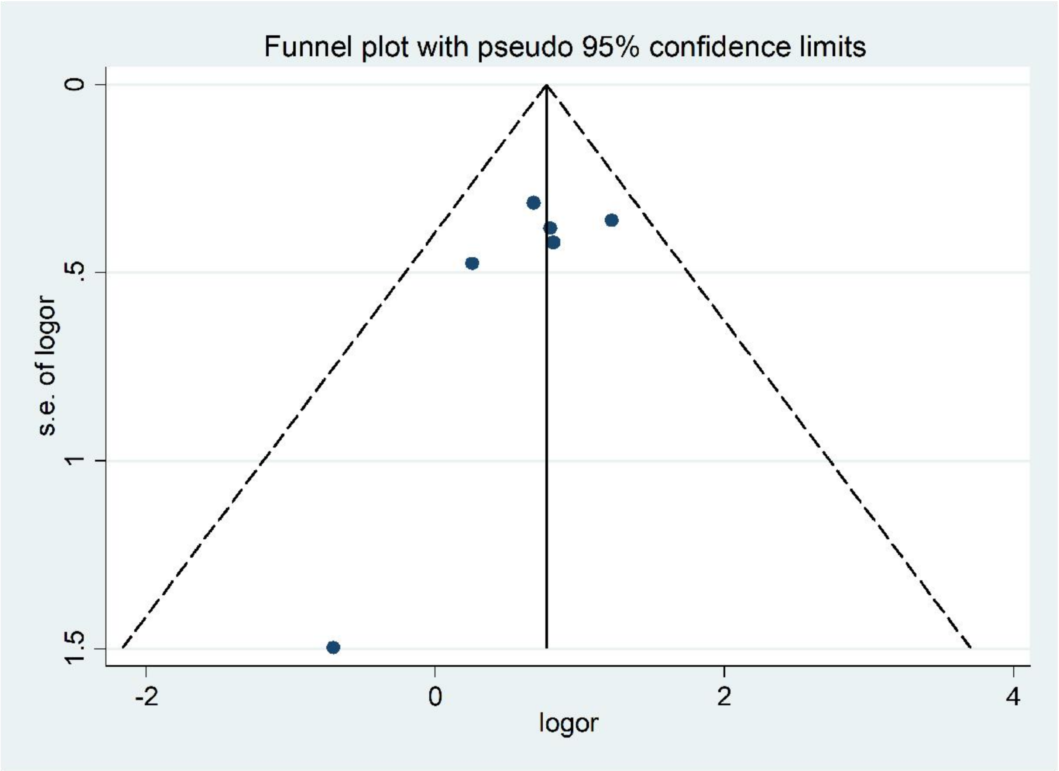

Figure S61: Funnel plot of differences in the manifestation (interlobular septal thickening) of lung lesions on chest images between COVID-19 patients and influenza patients

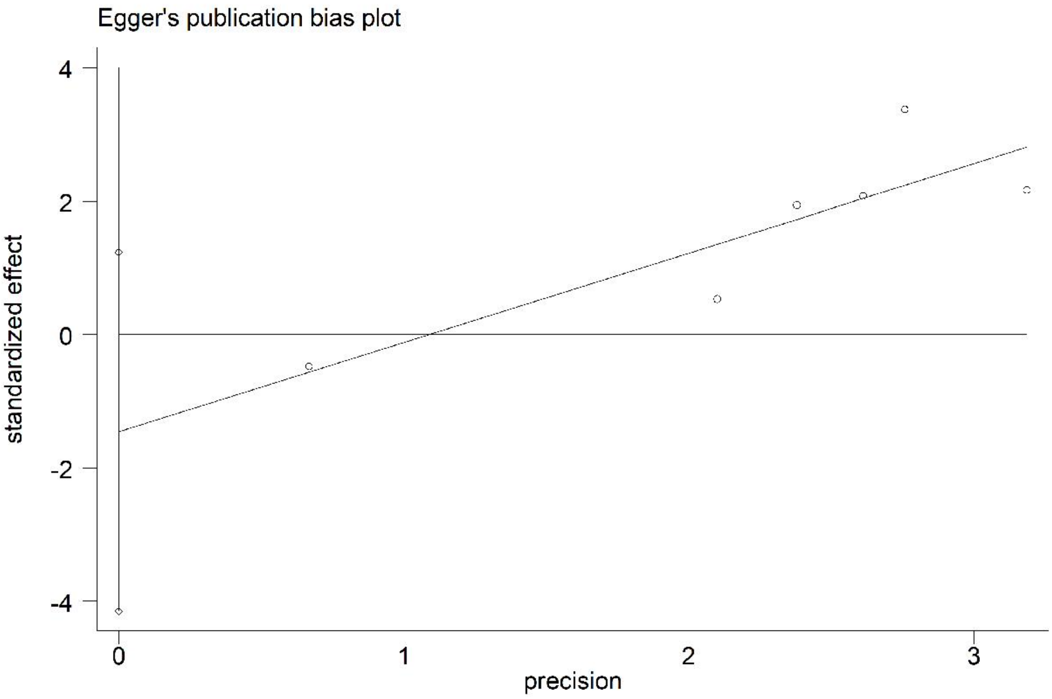

Figure S62: Egger' s publication bias plot of differences in the manifestation (interlobular septal thickening) of lung lesions on chest images between COVID-19 patients and influenza patients (p=0.208)

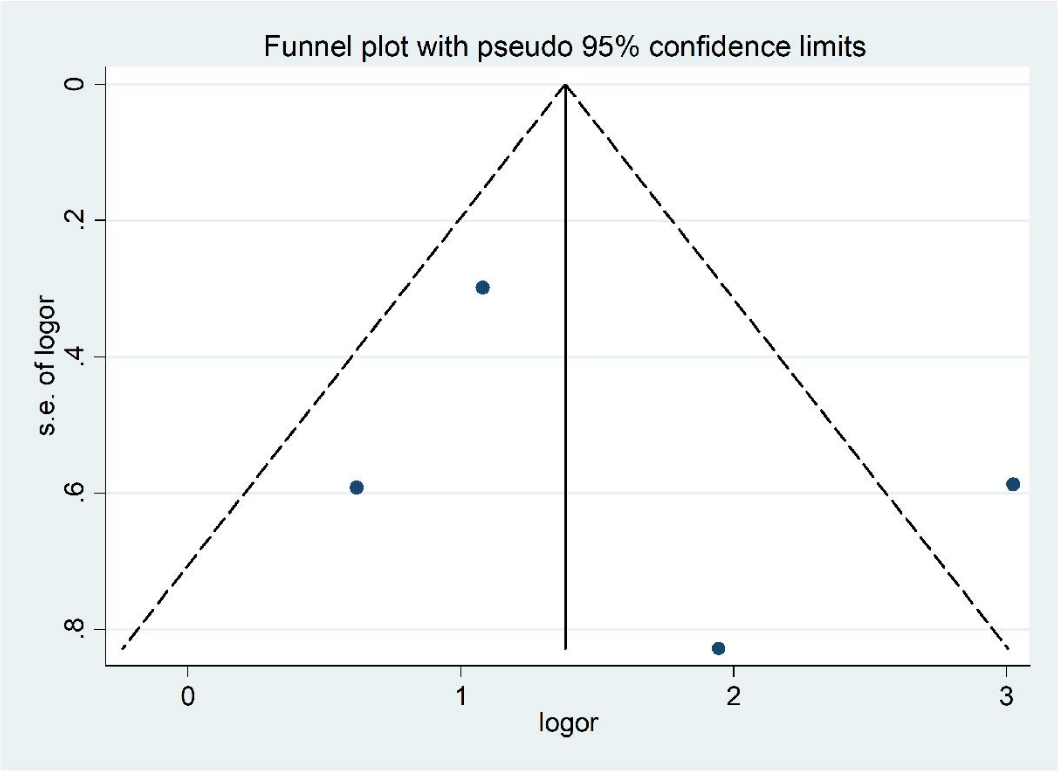

Figure S63: Funnel plot of differences in the manifestation (vascular enlargement) of lung lesions on chest images between COVID-19 patients and influenza patients

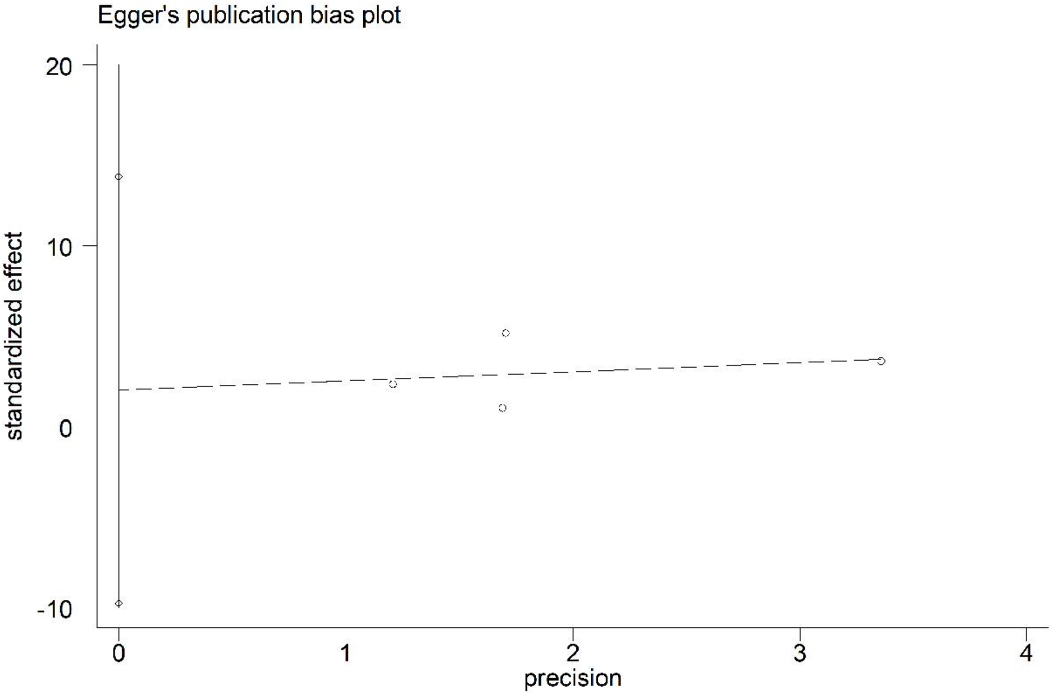

Figure S64: Egger’s publication bias plot of differences in the manifestation (vascular enlargement) of lung lesions on chest images between COVID-19 patients and influenza patients (p=0.533)

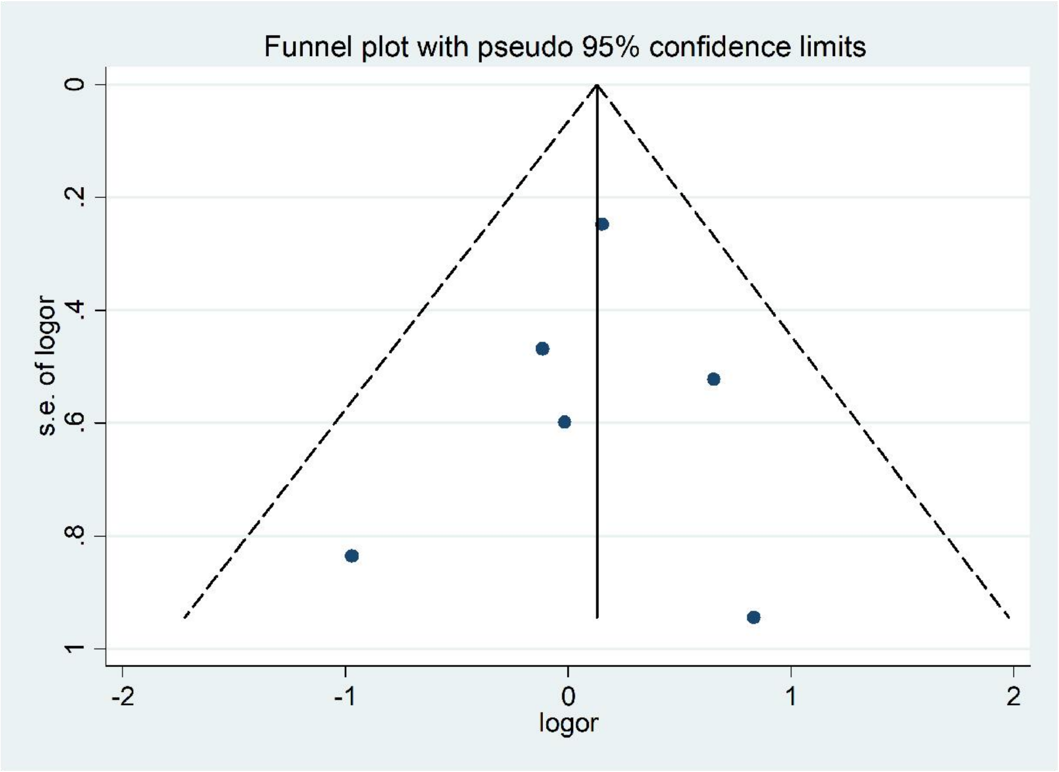

Figure S65: Funnel plot of differences in the manifestation (halo signs) of lung lesions on chest images between COVID-19 patients and influenza patients

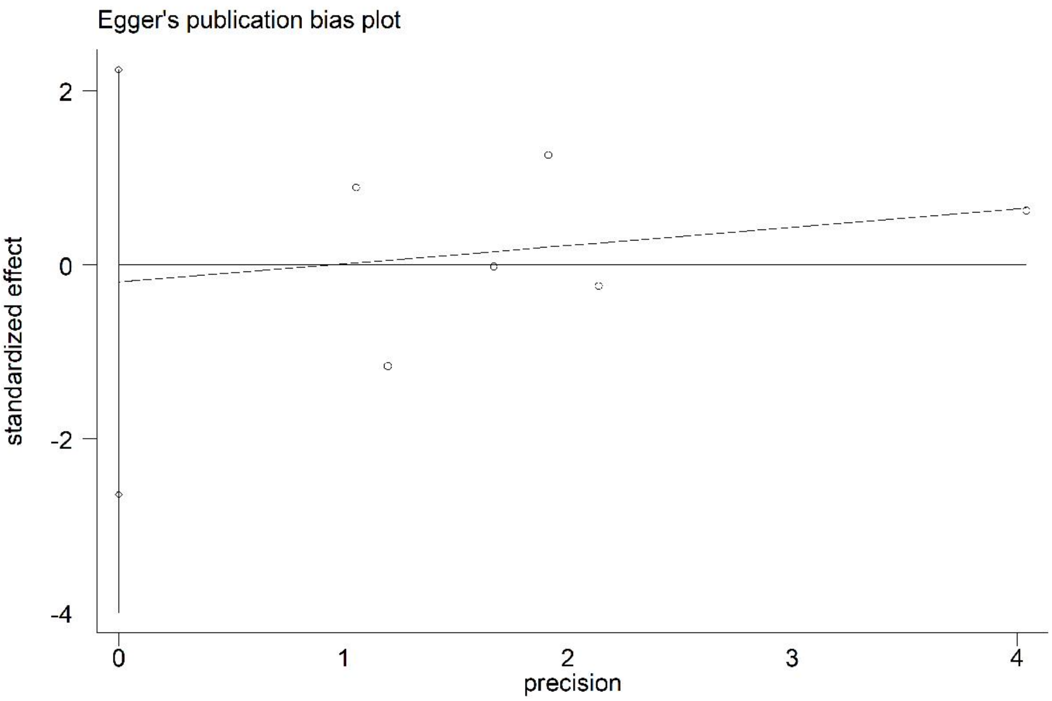

Figure S66: Egger's publication bias plot of differences in the manifestation (halo signs) of lung lesions on chest images between COVID-19 patients and influenza patients (p=0.831)

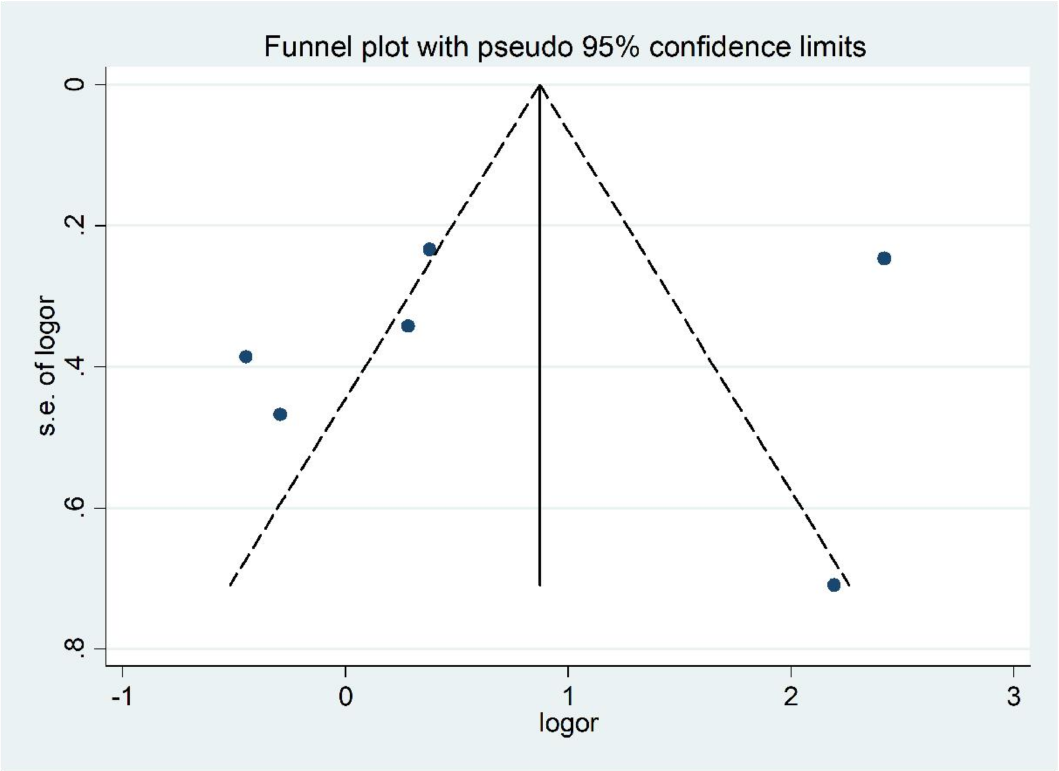

Figure S67: Funnel plot of differences in the manifestation (linear opacities) of lung lesions on chest images between COVID-19 patients and influenza patients

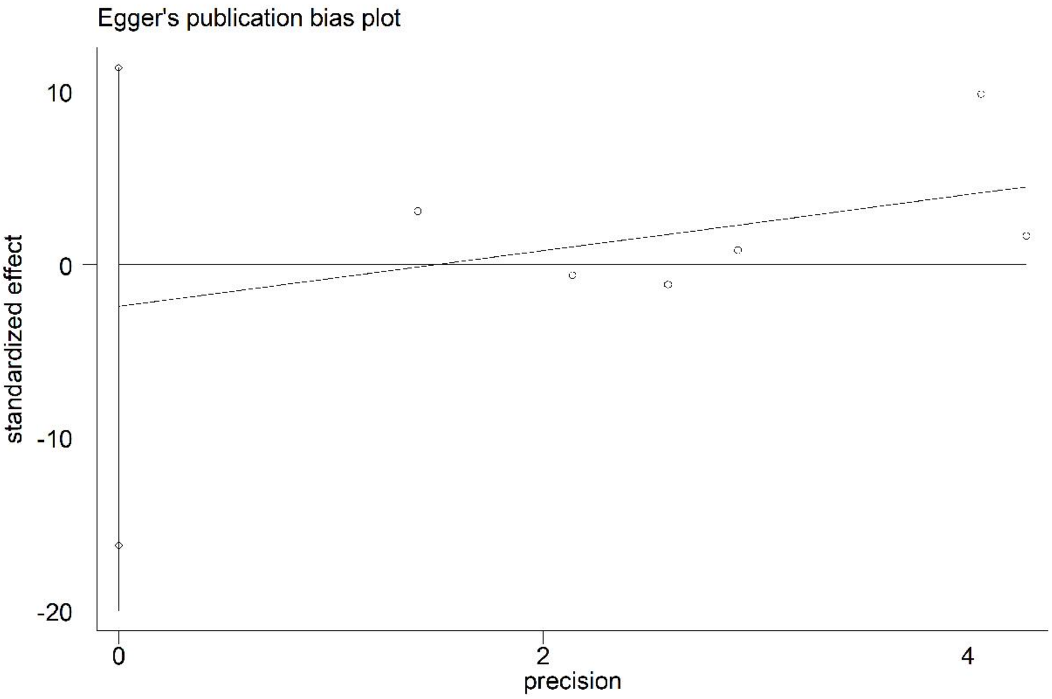

Figure S68: Egger's publication bias plot of differences in the manifestation (linear opacities) of lung lesions on chest images between COVID-19 patients and influenza patients (p=0.652)

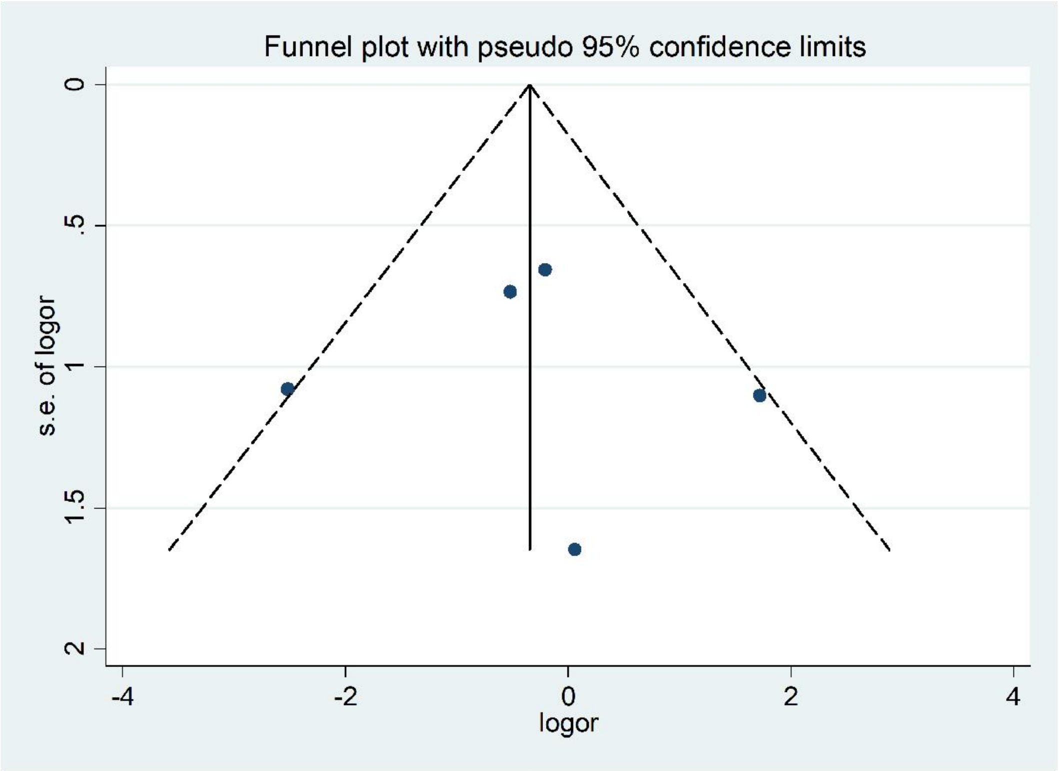

Figure S69: Funnel plot of differences in the manifestation (cavitation) of lung lesions on chest images between COVID-19 patients and influenza patients

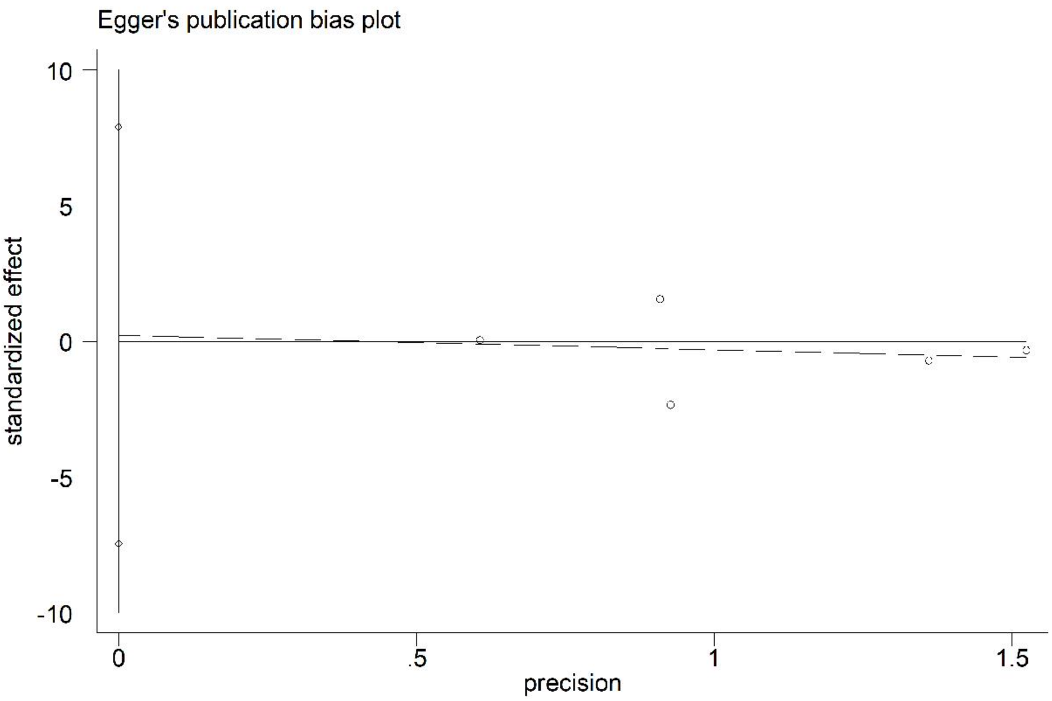

Figure S70: Egger' s publication bias plot of differences in the manifestation (cavitation) of lung lesions on chest images between COVID-19 patients and influenza patients (p=0.933)

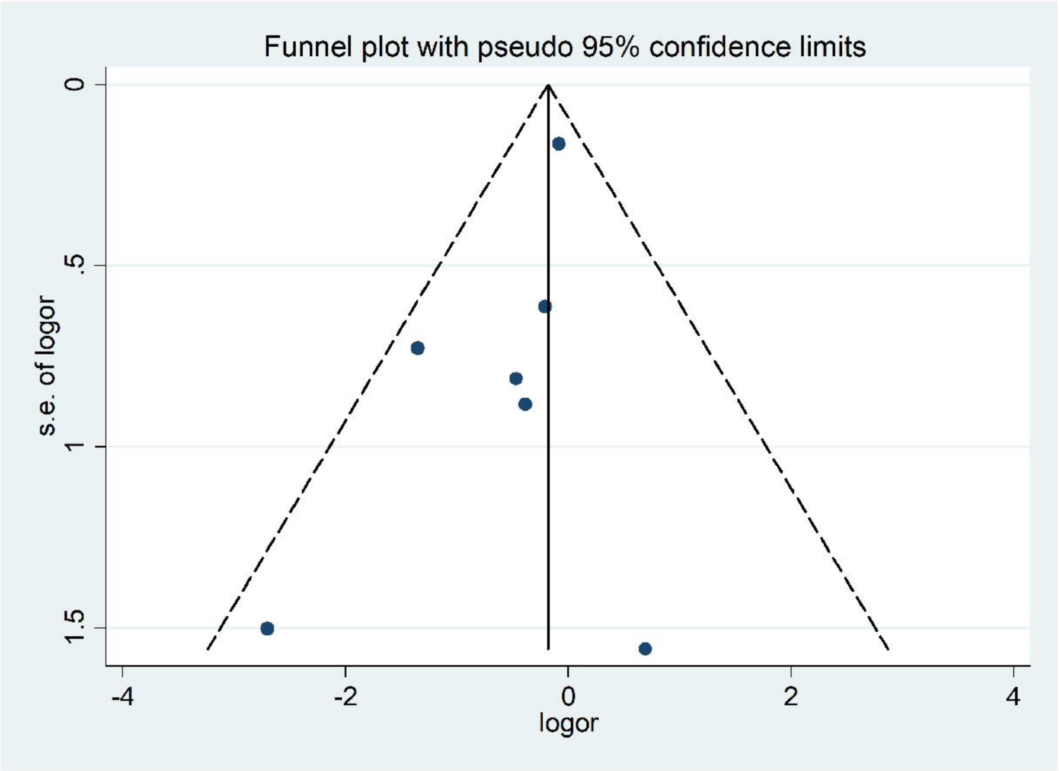

Figure S71: Funnel plot of differences in the manifestation (lymphadenopathy) of lung lesions on chest images between COVID-19 patients and influenza patients

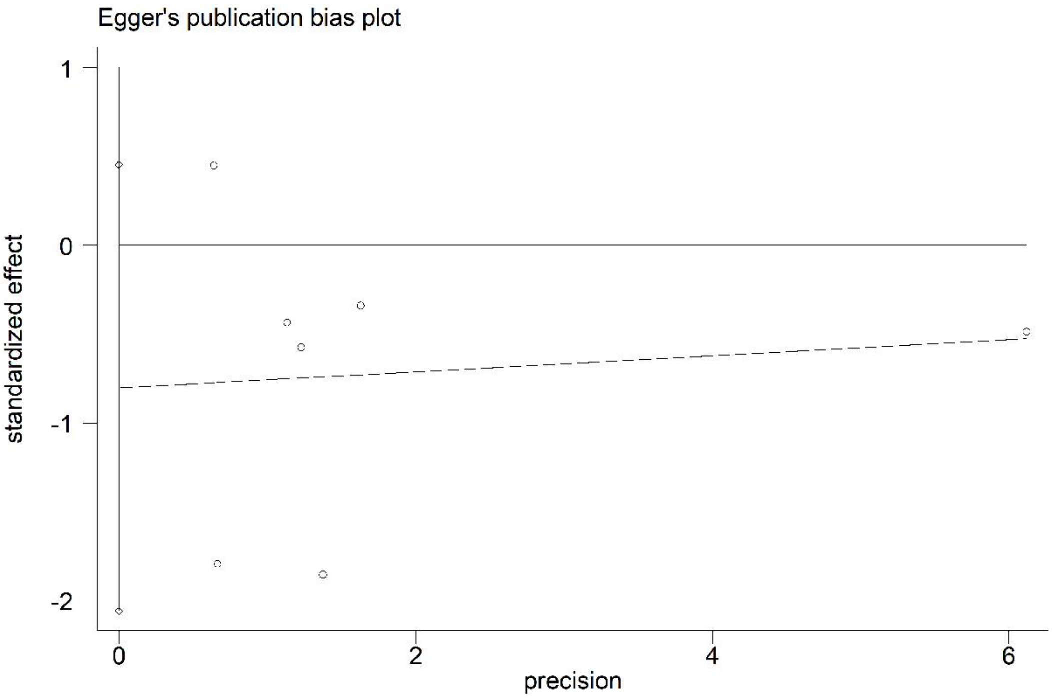

Figure S72: Egger's publication bias plot of differences in the manifestation (lymphadenopathy) of lung lesions on chest images between COVID-19 patients and influenza patients (p=0.161)

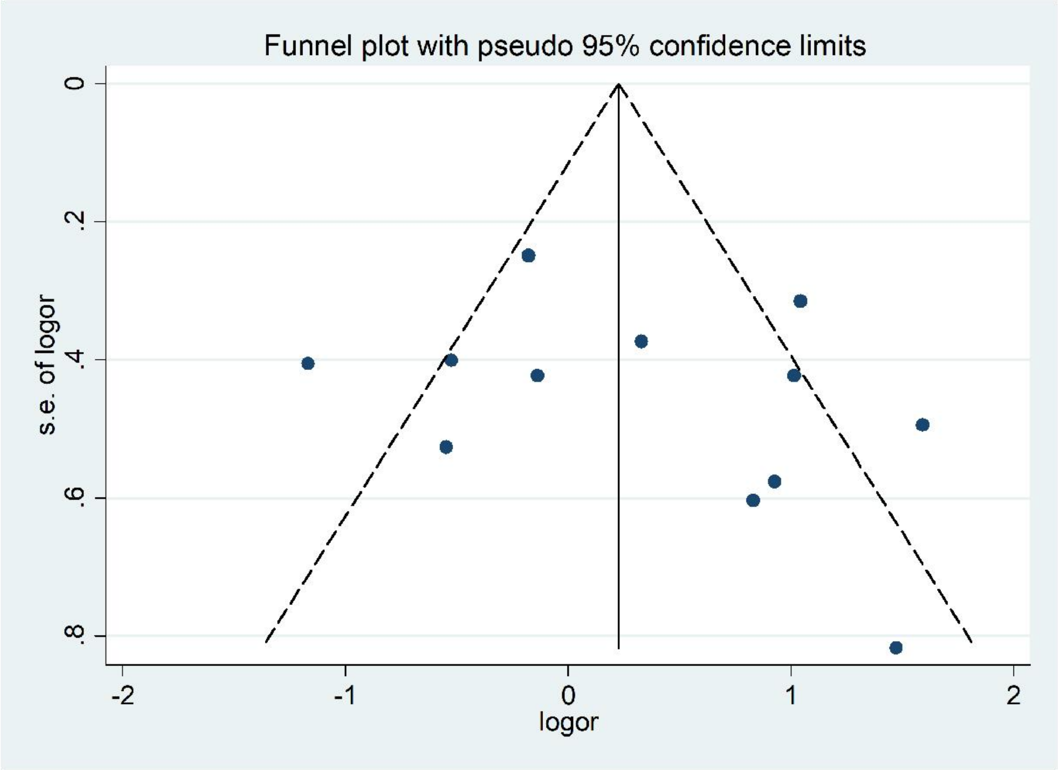

Figure S73: Funnel plot of differences in the manifestation (air bronchogram) of lung lesions on chest images between COVID-19 patients and influenza patients

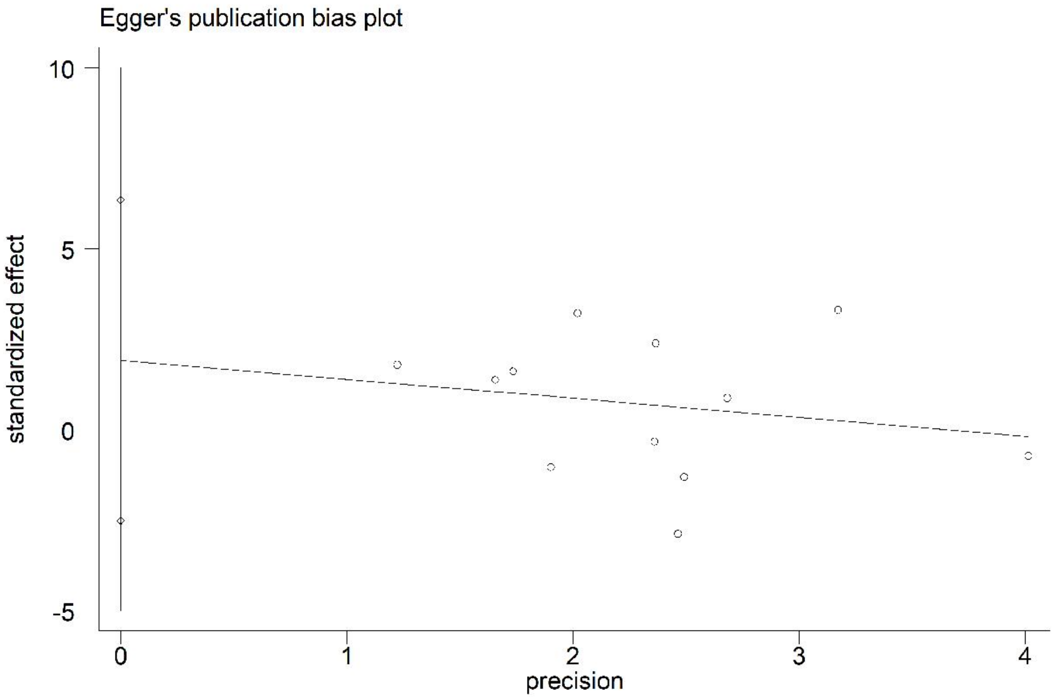

Figure S74: Egger’s publication bias plot of differences in the manifestation (air bronchogram) of lung lesions on chest images between COVID-19 patients and influenza patients (p=0.357)

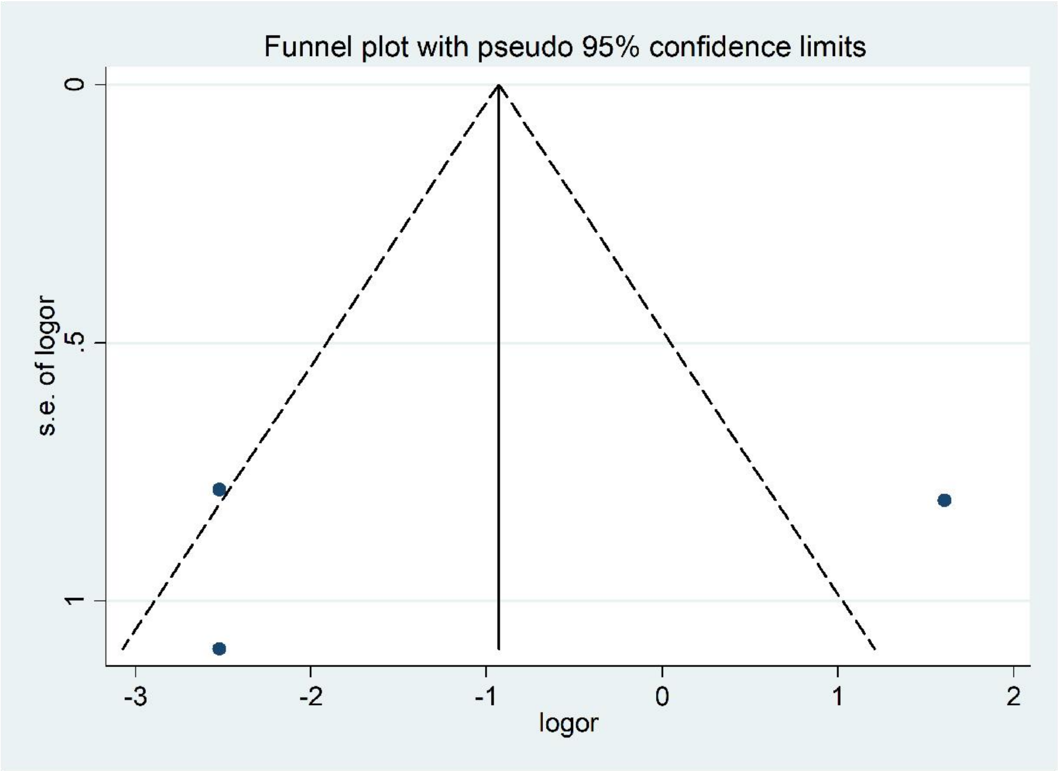

Figure S75: Funnel plot of differences in the manifestation (bronchiectasis) of lung lesions on chest images between COVID-19 patients and influenza patients

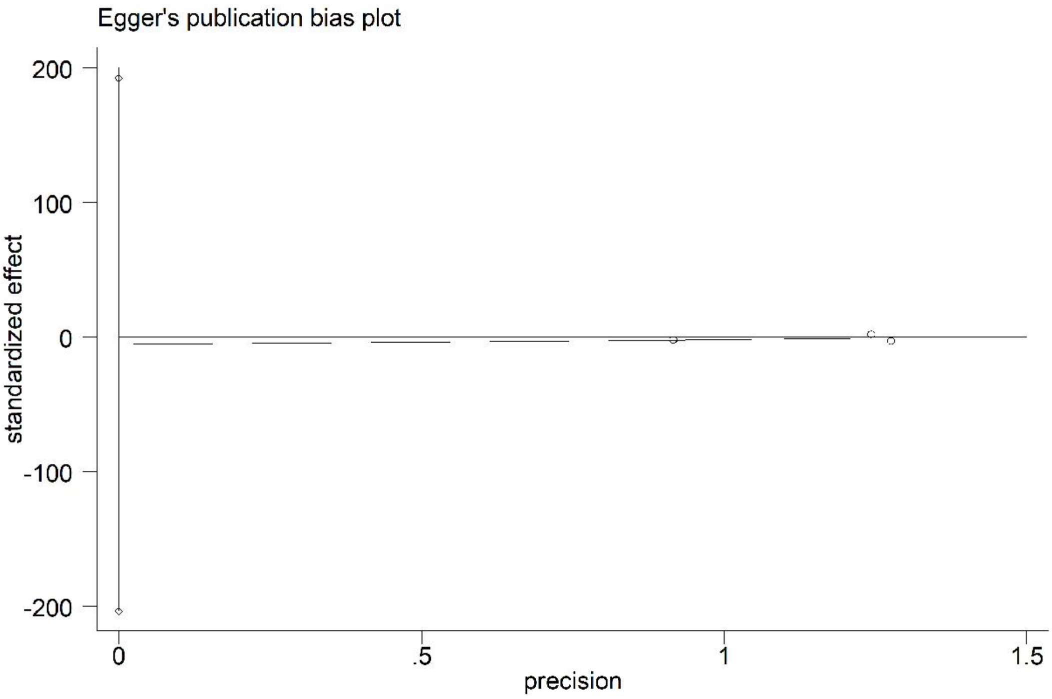

Figure S76: Egger's publication bias plot of differences in the manifestation (bronchiectasis) of lung lesions on chest images between COVID-19 patients and influenza patients (p=0.781)

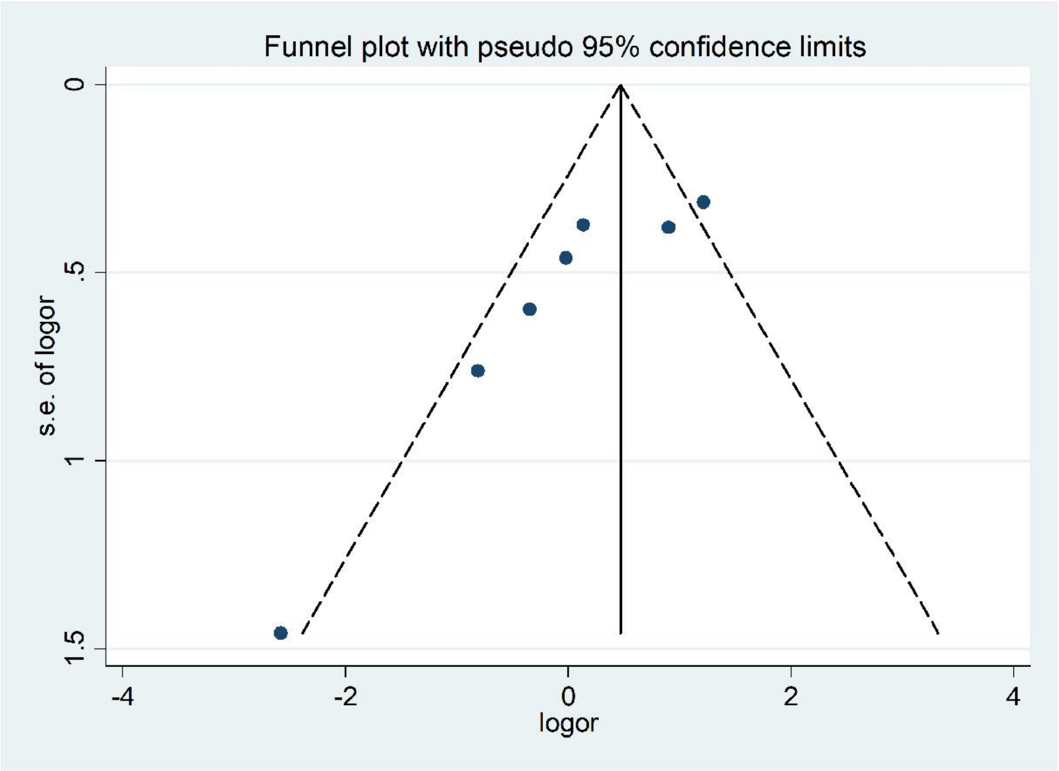

Figure S77: Funnel plot of differences in the manifestation (bronchial wall thickening) of lung lesions on chest images between COVID-19 patients and influenza patients

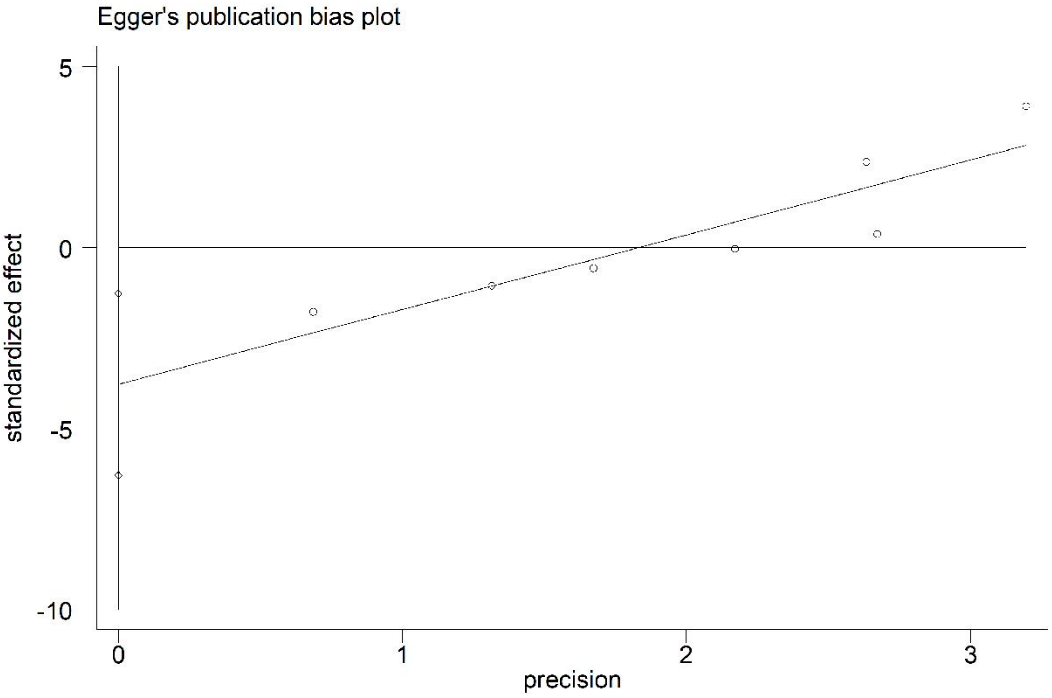

Figure S78: Egger's publication bias plot of differences in the manifestation (bronchial wall thickening) of lung lesions on chest images between COVID-19 patients and influenza patients (p=0.012)

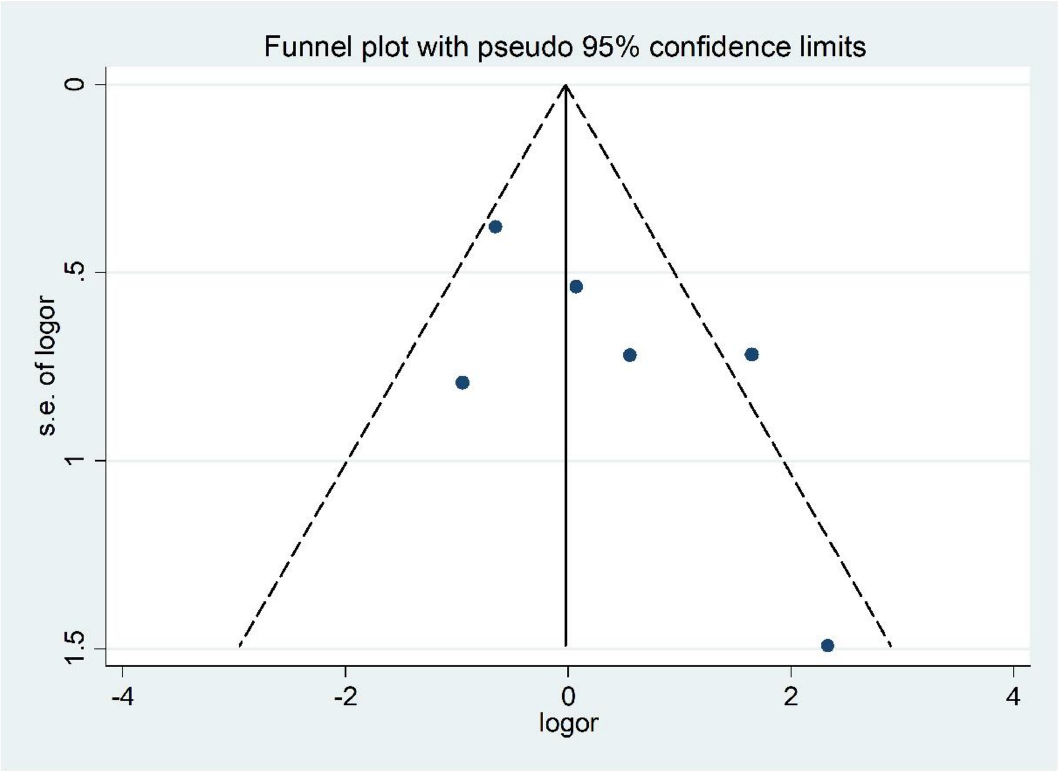

Figure S79: Funnel plot of differences in the manifestation (pleural thickening) of lung lesions on chest images between COVID-19 patients and influenza patients

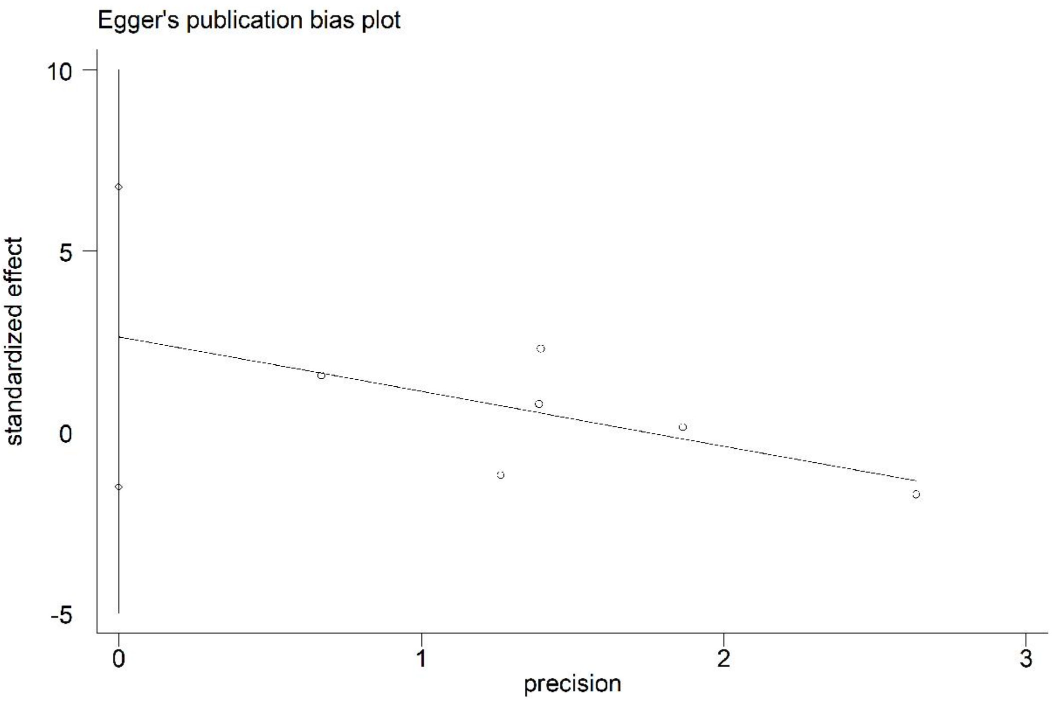

Figure S80: Egger's publication bias plot of differences in the manifestation (pleural thickening) of lung lesions on chest images between COVID-19 patients and influenza patients (p=0.152)
